# Mitigating the impact of image processing variations on tumour [^18^F]-FDG-PET radiomic feature robustness

**DOI:** 10.1038/s41598-024-67239-8

**Published:** 2024-07-15

**Authors:** Syafiq Ramlee, Roido Manavaki, Luigi Aloj, Lorena Escudero Sanchez

**Affiliations:** 1 Department of Radiology, School of Clinical Medicine, University of Cambridge, Cambridge Biomedical Campus, Cambridge, UK; 2 Department of Radiology, Cambridge University Hospitals NHS Foundation Trust, Cambridge, UK; 3 Cancer Research UK Cambridge Centre, University of Cambridge, Cambridge, UK

**Keywords:** Cancer imaging, Computational science, Biomarkers, Molecular imaging

## Abstract

Radiomics analysis of [^18^F]-fluorodeoxyglucose ([^18^F]-FDG) PET images could be leveraged for personalised cancer medicine. However, the inherent sensitivity of radiomic features to intensity discretisation and voxel interpolation complicates its clinical translation. In this work, we evaluated the robustness of tumour [^18^F]-FDG-PET radiomic features to 174 different variations in intensity resolution or voxel size, and determined whether implementing parameter range conditions or dependency corrections could improve their robustness. Using 485 patient images spanning three cancer types: non-small cell lung cancer (NSCLC), melanoma, and lymphoma, we observed features were more sensitive to intensity discretisation than voxel interpolation, especially texture features. In most of our investigations, the majority of non-robust features could be made robust by applying parameter range conditions. Correctable features, which were generally fewer than conditionally robust, showed systematic dependence on bin configuration or voxel size that could be minimised by applying corrections based on simple mathematical equations. Melanoma images exhibited limited robustness and correctability relative to NSCLC and lymphoma. Our study provides an in-depth characterisation of the sensitivity of [^18^F]-FDG-PET features to image processing variations and reinforces the need for careful selection of imaging biomarkers prior to any clinical application.

## Introduction

Radiological images harbour clinically imperceptible information about disease, which can be revealed through radiomics, the high-throughput extraction of quantitative features from these images^1^. Radiomics research involving positron emission tomography (PET), in particular with fluorine-18-labelled fluorodeoxyglucose ([^18^F]-FDG) as the imaging agent, is rapidly growing^2^ and holds promise in the prediction of disease progression^3^, metastasis^4^, survival^5,6^, and treatment response^7^—for a wide variety of cancer patients^2^. However, radiomic features can be sensitive to variations in imaging protocol and processing pipeline within and across institutions, ultimately challenging its translation into the clinic^8,9^.

The lack of reproducibility underscores the importance of feature robustness testing to develop reliable imaging biomarkers. Various parameters can affect radiomic measurements, including intensity resolution and voxel size, which are typically modified through discretisation and interpolation, respectively, to serve different purposes: noise reduction^10^, data compression^11^, image fusion with other modalities^12,13^, and facilitating multi-centre comparisons^14^. Several reports have interrogated the robustness of [^18^F]-FDG-PET radiomic features as a function of these two image processing parameters^10,15–18^. However, many of these investigations fall short of testing strategies to mitigate feature sensitivity to variations in these parameters, which would help enhance our understanding on whether datasets can be corrected and pooled for quantitative analyses^19^.

Non-robustness of features can be addressed via image-based solutions, which are applied directly to the images before the radiomics extraction process, or feature-based methods, applied to the downstream extracted feature values^20^. Previous reports contend that feature dependencies on voxel size or intensity bin configuration can be minimised either by modifying heterogeneously acquired images to utilise a fixed value for these parameters^16,21,22^, or through correction of radiomic features using pre-defined equations that account for these dependencies^21–24^. A correction factor based on the percentage change of feature values between interpolated and reconstructed PET images has also been suggested^17^.

However, improving feature robustness by introducing a shared imaging parameter across datasets poses the question of what parameter value(s) would be suitable for different sets of features, images, or investigations. There are currently no guidelines to help choose optimal intensity bin configurations and voxel sizes, and a comprehensive characterisation of the behaviour of radiomic features against these two parameters is necessary to help establish this^25^. Second, knowledge of feature definitions is usually required to reap suitable equations for correction, which is challenging as definitions vary between radiomics extraction software platforms^26^. While work to standardise feature definitions by the Imaging Biomarker Standardisation Initiative (IBSI) is ongoing^27^, a recent study contests that these efforts still do not guarantee feature reproducibility between softwares^28^. Third, use of reconstructed voxel size as the ground truth^17^ cannot be easily generalised to other images where the reconstructed voxel sizes may be different. Thus, a correction technique that is generalisable across platforms and does not require any form of ground truth is desirable.

The objectives of this work were four-fold: (i) to characterise the robustness of [^18^F]-FDG-PET radiomic features against varying intensity discretisation and voxel interpolation parameters; (ii) to investigate whether feature robustness could be improved by a novel solution based on imposing range conditions on these parameters; (iii) to test a software-agnostic feature correction method that minimises the sensitivity of radiomic features to variations of these parameters; and (iv) to explore feature robustness across different cancer types, i.e., non-small cell lung cancer (NSCLC), lymphoma, and melanoma.

## Materials and methods

**Figure 1:**
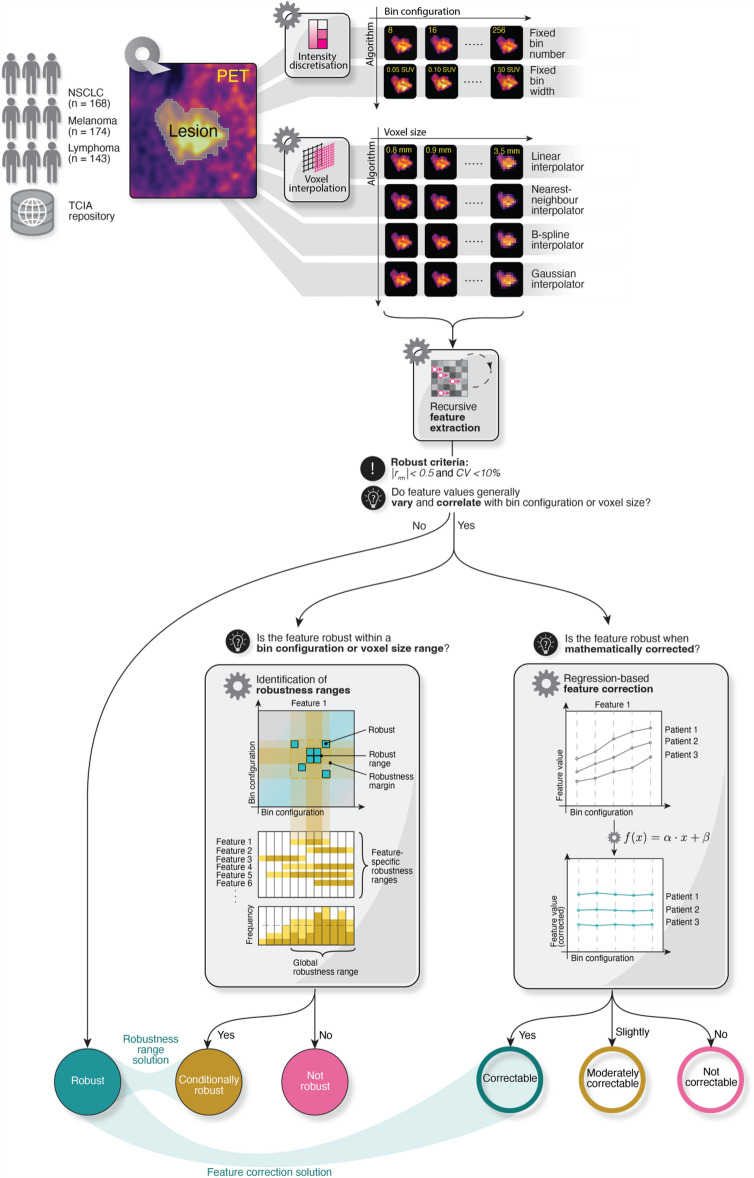
Schematic representation of the study workflow.

A graphic summary of the study design is illustrated in Fig. 1.

### Dataset

Nine hundred anonymised whole-body [^18^F]-FDG PET/CT images together with manually segmented tumour volumes were obtained from a highly curated imaging dataset publicly available at The Cancer Imaging Archive (TCIA)^29–31^. Patients without annotated tumour volumes, or with volumes too small (< 15 voxels) to extract valid radiomic features or too large (> 200,000 voxels) to overcome computational memory limitations were excluded. We considered one examination per patient and treated each patient segmentation mask as a single volume. After exclusions, a total of 485 patients with [^18^F]-FDG-avid lesions were included in this study, which spanned three cancer types: NSCLC (*n* = 168), melanoma (*n* = 174), and lymphoma (*n* = 143). The mean (range) of analysed tumour voxels was 21,189 (58–178,944), 8926 (31–128,920), and 24,238 (98–199,437), respectively. Corresponding tumour volumes were 264.5 (0.72–2234.1) cm^3^, 111.4 (0.44–1609.5) cm^3^, and 302.6 (1.22–2489.9) cm^3^.

Acquisition and reconstruction protocols for this dataset have previously been described^30^. In brief, images were acquired on a *Biograph mCT* scanner (*Siemens Healthineers*) at 60 min post [^18^F]-FDG injection, and reconstructed into a 400 × 400 matrix with 2.04 × 2.04 × 3 mm^3^ voxels, using ordered-subset expectation maximisation (21 subsets, 2 iterations), and a 2-mm FWHM Gaussian filter post-reconstruction. As a pre-processing step, PET images were converted from Digital Imaging and Communications in Medicine (DICOM) to Neuroimaging Informatics Technology Initiative (NIfTI) format, and subsequently normalised for injected activity and body weight to generate standardised uptake value (SUV) maps using scripts provided with the dataset^29^.

### Image processing

Radiomic features were tested for robustness against parameter variations in two image processing schemes: intensity discretisation and voxel interpolation. Intensity discretisation was studied either by fixing bin widths (FBW) or fixing bin numbers (FBN). FBW discretisation utilised intensity bin widths ranging from 0.05 to 1.50 SUV at 0.05 SUV step intervals, while the number of bins for FBN discretisation ranged from 8 to 256 bins at 8-bin step intervals—thus, yielding 30 and 32 parameters for each discretisation scheme, respectively. All other image parameters were kept at the original values.

The effect of voxel interpolation on radiomic features was tested by employing four common interpolators: linear, nearest-neighbour, B-spline, and Gaussian. Voxels were resampled to isotropic sizes ranging from 0.8 to 3.5 mm at step intervals of 0.1 mm, yielding 28 values to test for each interpolation method. Image intensities were discretised at 64 bins, as commonly used in the literature, while all other parameters were kept at the original values.

In total, 174 image processing parameters were considered in this paper.

### Recursive feature extractions

Using *PyRadiomics*^32^*,* version 3.0.1*,* a total of 107 IBSI-compliant radiomic features from the following families were extracted: shape-based (*n* = 14), first-order statistics (*n* = 18), grey-level co-occurrence matrix (GLCM) (*n* = 24), grey-level dependence matrix (GLDM) (*n* = 14), grey-level run-length matrix (GLRLM) (*n* = 16), grey-level size zone matrix (GLSZM) (*n* = 16), and neighbouring grey-tone dependence matrix (NGTDM) (*n* = 5). GLCM and GLRLM features were calculated by computing the corresponding matrices over 13 spatial directions in 3D (26-connectivity), with a single voxel offset for the former, after which the values were averaged to make them rotationally invariant. Feature families pre-processed with mathematical filters (higher-order features) were not evaluated. Following a recursive extraction of radiomic features from each of the 485 patient images across the 174 image processing parameters, a total of 9,029,730 feature values were obtained and analysed. Extractions were performed on Python, version 3.10.8 (Python Software Foundation, https://www.python.org).

### Assessment of robustness of features

Robustness of radiomic features was assessed in terms of both the agreement and reliability of measurements^33,34^. The agreement of feature values against parameter variations (i.e., *feature variability*) was measured using the *within-subject percentage coefficient of variation,* which was subsequently averaged across patients to generate a mean value (*CV*_*mean*_) per feature. Feature reliability was assessed by means of *feature correlation,* using the magnitude of the *repeated measures correlation coefficient* (|*r*_*rm*_|). This correlation coefficient was chosen over other measures (e.g., Pearson, Spearman) as it addresses the non-independence of feature values across parameters^35^. Empirical thresholds of *CV*_*mean*_ < 10% and |*r*_*rm*_|< 0.5 were used to define the robust criteria^22,23,36–38^ and, in turn, determine *robust* features.

### Derivation of feature-specific robustness ranges

For features not meeting the robust criteria, *CV*_*mean*_ and |*r*_*rm*_| were systematically evaluated for all possible parameter ranges. For each interpolator and a range of voxel sizes between 0.8 and 3.5 mm (i.e., 28 voxel sizes), (^*28*^*C*_*2*_ =) 378 distinct ranges could be interrogated for conditional robustness (e.g., 0.8 to 0.9 mm, 0.8 to 1.0 mm, etc.). This number was (^*30*^*C*_*2*_ =) 435 and (^*32*^*C*_*2*_ =) 496 for FBW and FBN discretisation, respectively. Parameter ranges were represented as tiles on a square correlogram or matrix (Fig. 1), and a robustness matrix was generated for each feature under different scenarios (image processing algorithm, cancer type). Excluding those on the diagonal, robust tiles, i.e., matrix elements for which the robust criteria were met (*CV*_*mean*_ < 10% and |*r*_*rm*_|< 0.5), defined a range for which a feature was deemed *conditionally robust.* Features for which no robust tiles could be identified were categorised as *non-robust*.

To determine range conditions from the robustness matrix for conditionally robust features, two nested square regions, symmetrical about the diagonal, were delineated on the matrix, defining the *robust range* and *robustness margin*, respectively. The robust range, representing the limits of the largest closed square region containing a contiguous cluster of robust tiles, was defined via run length encoding in the horizontal and vertical directions. The contiguity constraint for the robust range was imposed to exclude tiles with spurious *CV*_*mean*_ or |*r*_*rm*_| values. The robustness margin was determined by creating a non-contiguous square region using the two outermost parameter values where at least one robust tile exists, excluding tiles on the diagonal. The robust range and robustness margin, together, make up the *robustness range* for each feature.

### Derivation of global robustness ranges

Given that a typical radiomics study would use the same discretisation and interpolation settings for the collective extraction of radiomic features, a “global” parameter range was determined by aggregating robustness ranges of conditionally robust features into a histogram, and applying a cut-off recovering at least half of these features per scenario.

### Identification of correctable features

As an alternative to imposing range conditions, we tested radiomic feature correction as a potential strategy to improve the robustness of features initially not meeting the robust criteria. The relationship between feature values and bin configuration or interpolated voxel size was modelled using eight regression functions, *f(x)*, as first utilised in^23^: *f(x)* = *α · x* + *β, f(x)* = *α · x*^*2*^ + *β, f(x)* = *α · x*^*3*^ + *β, f(x)* = *α/x* + *β, f(x)* = *α/x*^*2*^ + *β, f(x)* = *α/x*^*3*^ + *β, f(x)* = *α · log(x)* + *β*, and *f(x)* = *α/log(x)* + *β* where *α* and *β* are fit parameters respectively, and *x* is the parameter under investigation (i.e., bin configuration or voxel size).

Functions were fitted using robust regression with an iteratively reweighted least squares algorithm, applying intrinsic weights in the form of the reciprocal of feature variance values computed across patients. The function achieving the lowest value for the Akaike information criterion (AIC) amongst the ones tested was selected as best describing the dependencies of feature values on bin configuration or voxel size. Feature corrections for each patient were implemented by using a rearranged form of the best-fitting function^23^, e.g., *f(x)* = *α · log(x)* + *β, f*_*corrected*_*(x)* = *(f(x) − β)/log(x).* When computing regressions for logarithmic functions, bin widths and voxel sizes were multiplied by 100 and 10, respectively, to circumvent division by zero errors when the x-variable equalled 1.

Wilcoxon signed-rank test was employed to compare *CV* values before and after correction, whereas the overlap between the 95% confidence intervals (CI) of the original and corrected |*r*_*rm*_| was examined. Corrections were deemed statistically significant when the p-value was less than 0.05 for the Wilcoxon signed-rank test, and the 95% CI of the |*r*_*rm*_| between original and corrected did not overlap. Features with a statistically significant reduction in *CV*_*mean*_ and |*r*_*rm*_| upon correction were segregated into two categories: *correctable*, if the robust criteria (*CV*_*mean, corrected*_ < 10 and |*r*_*rm, corrected*_|< 0.5) were satisfied; and *moderately correctable* if these criteria were not met. Features were otherwise classified as *not correctable.*

### Statistical analysis

The effect of discretisation scheme, interpolator, or cancer type on feature robustness or correctability was investigated using mixed-effects logistic regression with per-feature random intercepts, and results were reported as odds ratios (OR) with 95% CI, where appropriate. p-values from post-hoc analyses were adjusted for multiple comparisons using the Bonferroni method. Statistical significance was defined as p < 0.05. All statistical analyses were performed using R, version 4.2.2 (R Foundation for Statistical Computing, Vienna, Austria) (https://www.R-project.org/) or jamovi version 2.3 (https://www.jamovi.org)^39^.

## Results

### Robust features

**Figure 2:**
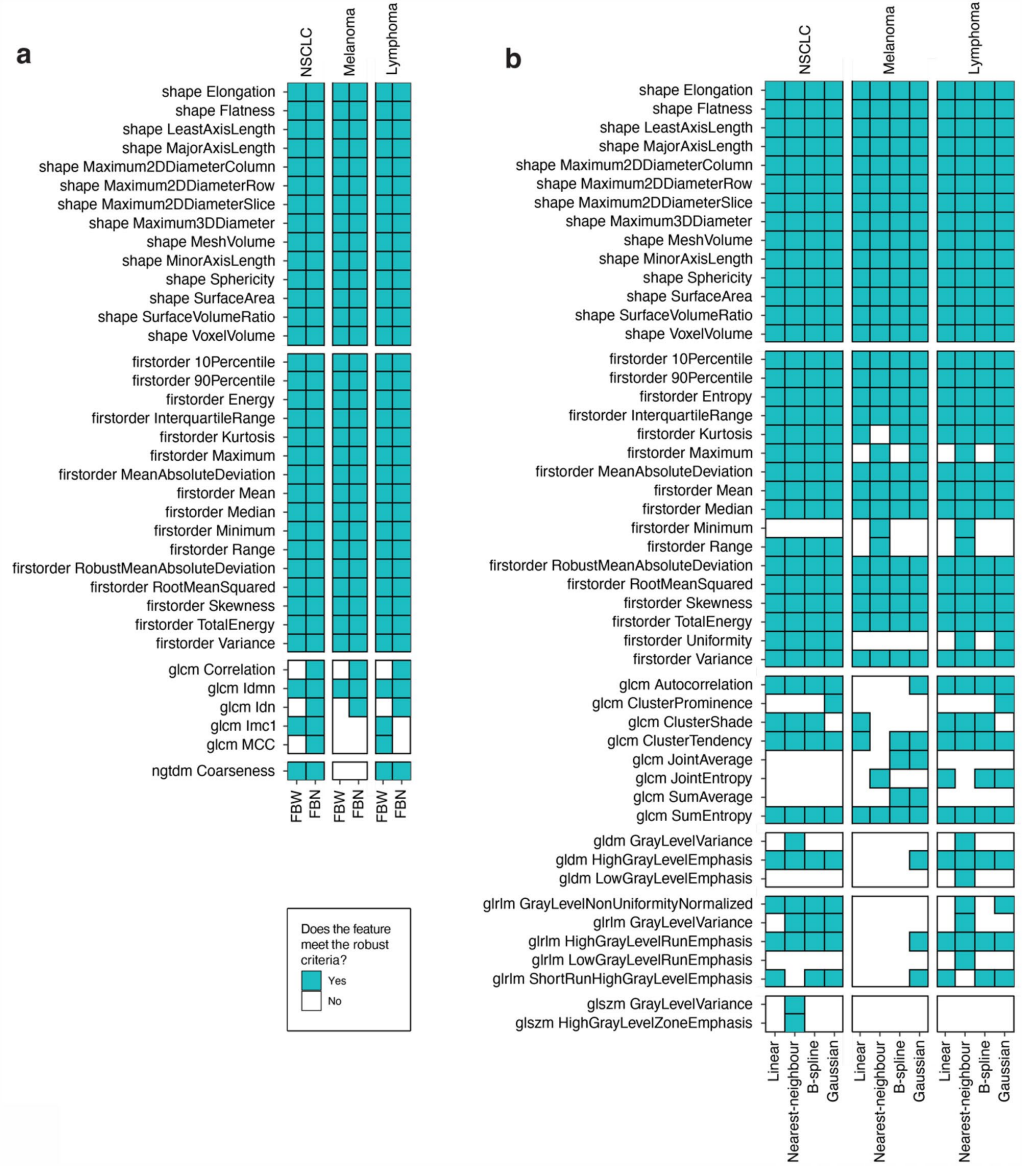
[^18^F]-FDG-PET radiomic features robust to intensity discretisation (**a**) or voxel interpolation (**b**) stratified by image processing algorithm used and cancer type.

Figure 2 illustrates [^18^F]-FDG-PET radiomic features that meet the robust criteria (*CV*_*mean*_ < 10 and |*r*_*rm*_|< 0.5), stratified by image processing scheme, algorithm, and cancer type.

Overall, in mixed effects logistic regression models including cancer type and image processing scheme as fixed effects with per-feature random intercepts, radiomic features were more sensitive to intensity discretisation than voxel interpolation (OR 0.57 [0.36, 0.29], p = 0.01), as also evidenced in Fig. 2. Specifically, a median 34/107 (32%) and 36/107 (34%) features were classified as robust following discretisation and interpolation, respectively (Supplementary Table ST1).

Between families, shape and first-order features were more robust than second-order ones (p < 0.001) across discretisation and interpolation schemes. Shape features were unaffected by the image processing schemes considered irrespective of cancer type. *Entropy* and *Uniformity* for discretisation, and *Energy* for interpolation, were the only first-order features not meeting the robust criteria in all tested instances. When considering second-order families, 5/24 (21%) GLCM, 1/5 (20%) NGTDM features, and none from the remaining families satisfied the robust criteria in at least one scenario of discretisation. These proportions were generally larger for interpolation with 8/24 (33%) GLCM, 3/14 (21%) GLDM, 5/16 (31%) GLRLM, 2/16 (13%) GLSZM, and no NGTDM features identified to be robust. Of these second-order features, only GLCM *idmn* (inverse difference moment normalised) or GLCM *SumEntropy* were robust across all tested instances of discretisation or interpolation, respectively.

FBW discretisation produced less robust features than FBN (OR 0.24 [0.05, 1.20]). However, the effect of the discretisation algorithm on robustness was not statistically significant (p = 0.08). Similarly, differences in robust categorisations between cancer types were not significant (p = 0.1), with melanoma producing the least number of robust features regardless of discretisation algorithm used (Supplementary Table ST1). The largest proportion of robust features was observed for FBN discretisation in NSCLC (36/107; 34%), and the smallest for FBW discretisation in melanoma (31/107; 29%).

In contrast, the choice of interpolator significantly affected feature robustness (p = 0.03). Use of nearest-neighbour interpolation was more likely to result in robust features than linear (OR 3.24 [1.30, 8.22]), B-spline (OR 2.59 [1.03, 6.49]), or Gaussian (OR 1.11 [0.46, 2.70]). However, upon Bonferroni correction for multiple comparisons, these differences were not statistically significant (nearest-neighbour vs. linear: p = 0.08; vs. B-spline: p = 0.25; vs. Gaussian: p = 1). Robust categorisations were significantly different between cancer types (p < 0.001), with melanoma showing greater sensitivity to interpolation than lymphoma (OR 0.16 [0.07, 0.39]) or NSCLC (OR 0.1 [0.04, 0.24]). Use of nearest-neighbour interpolation in lymphoma and linear interpolation in melanoma yielded the highest (42/107; 39%) and lowest number of robust features (30/107; 28%), respectively.

### Conditionally robust features

For those features initially not fulfilling the robust criteria, we tested the possibility of making them more robust by imposing parameter range conditions. *CV*_*mean*_ and |*r*_*rm*_| were evaluated across all intensity resolutions or voxel sizes considered, in a pairwise manner, and findings were compiled into a square matrix, which were subsequently used to compute the robustness ranges for each feature.

**Figure 3:**
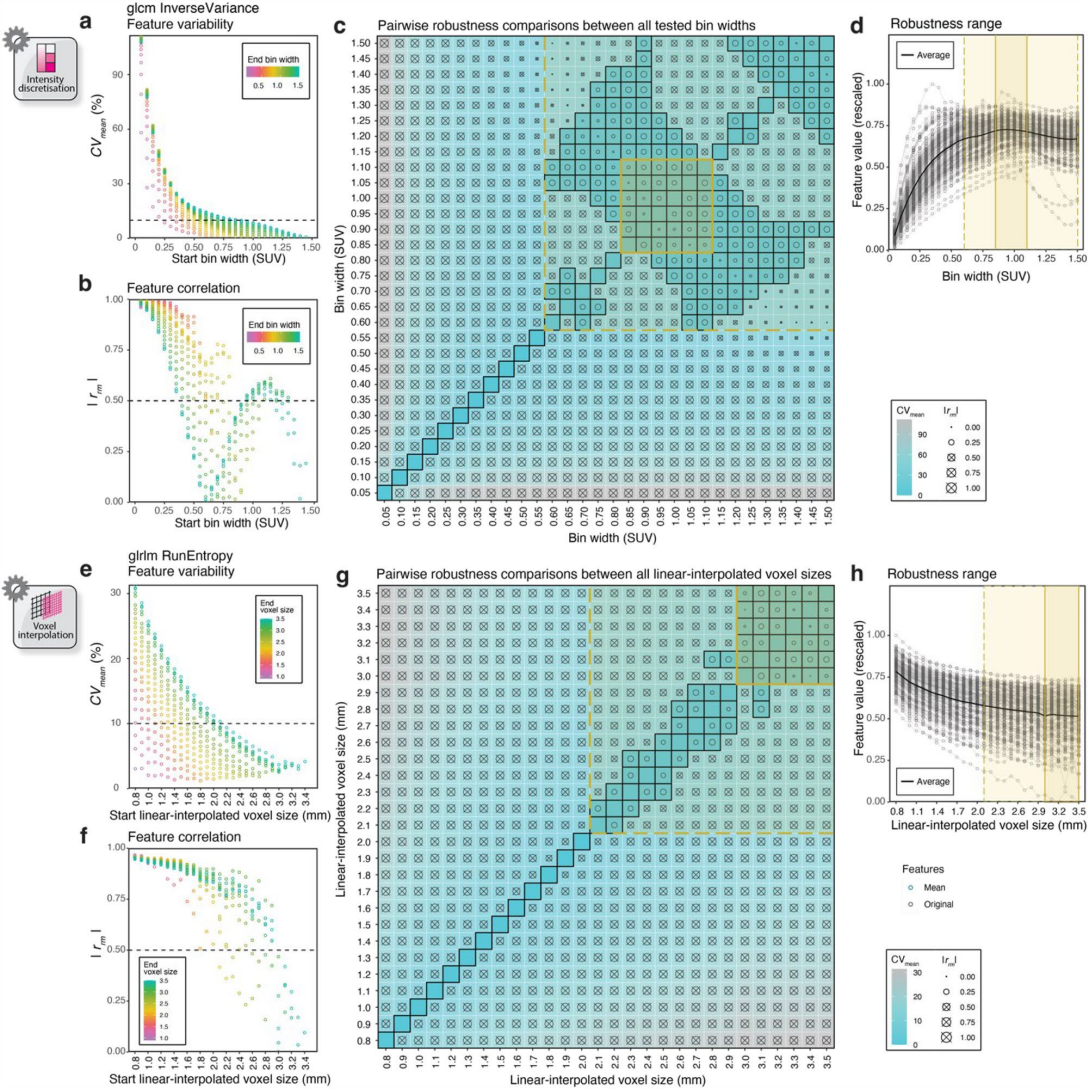
Scatter plots (left) and square correlograms (centre) for determining the robustness range conditions for GLCM *InverseVariance* as a function of bin width (**a**–**d**), and GLRLM *RunEntropy* as a function of linear-interpolated voxel size (**e**–**h**), for the NSCLC cases, as examples. In the scatter plots, *CV*_*mean*_ and |*r*_*rm*_| varied as a function of bin width (**a**,**b**) or linear-interpolated voxel size (**e**,**f**), and were dependent on the difference between the start and end bin width or linear-interpolated voxel size. By applying thresholds on these robustness metrics, start and end bin width or voxel size combinations satisfying the robust criteria were selected (represented as tiles with a black border on the square correlograms) as seen in (**c**) and (**g**). Two nested square regions that correspond to a contiguous and non-contiguous cluster of robust tiles were delineated on each correlogram, the limits of which indicate the robust range and robustness margin (outlined in yellow), respectively. Care was taken to exclude tiles on the diagonal, denoting combinations of either bin width or voxel size values with itself. The robustness ranges, wherein feature values were relatively stable (robustness margin) or stable (robust region), were overlaid onto plots showing feature response to bin width (**d**) or voxel size (**h**) variations.

Examples of two conditionally robust features from NSCLC cases are given in Fig. 3, which examine the response to FBW discretisation for GLCM *InverseVariance* (Fig. 3a–d) and linear interpolation for GLRLM *RunEntropy* (Fig. 3e–h). The process described in the examples was repeated to identify conditionally robust features for all discretisation schemes and interpolators across cancer types. Where no robustness range could be identified, features were classified as non-robust. Scatter and correlogram plots for both conditionally robust and non-robust features have been made publicly available in https://github.com/syafiqramlee/robust-radiomics-img-processing. For each of the 107 features, robustness categorisations throughout the scenarios considered in this study are presented in Supplementary Fig. SF1, with their corresponding proportions tabulated in Supplementary Table ST1.

**Figure 4:**
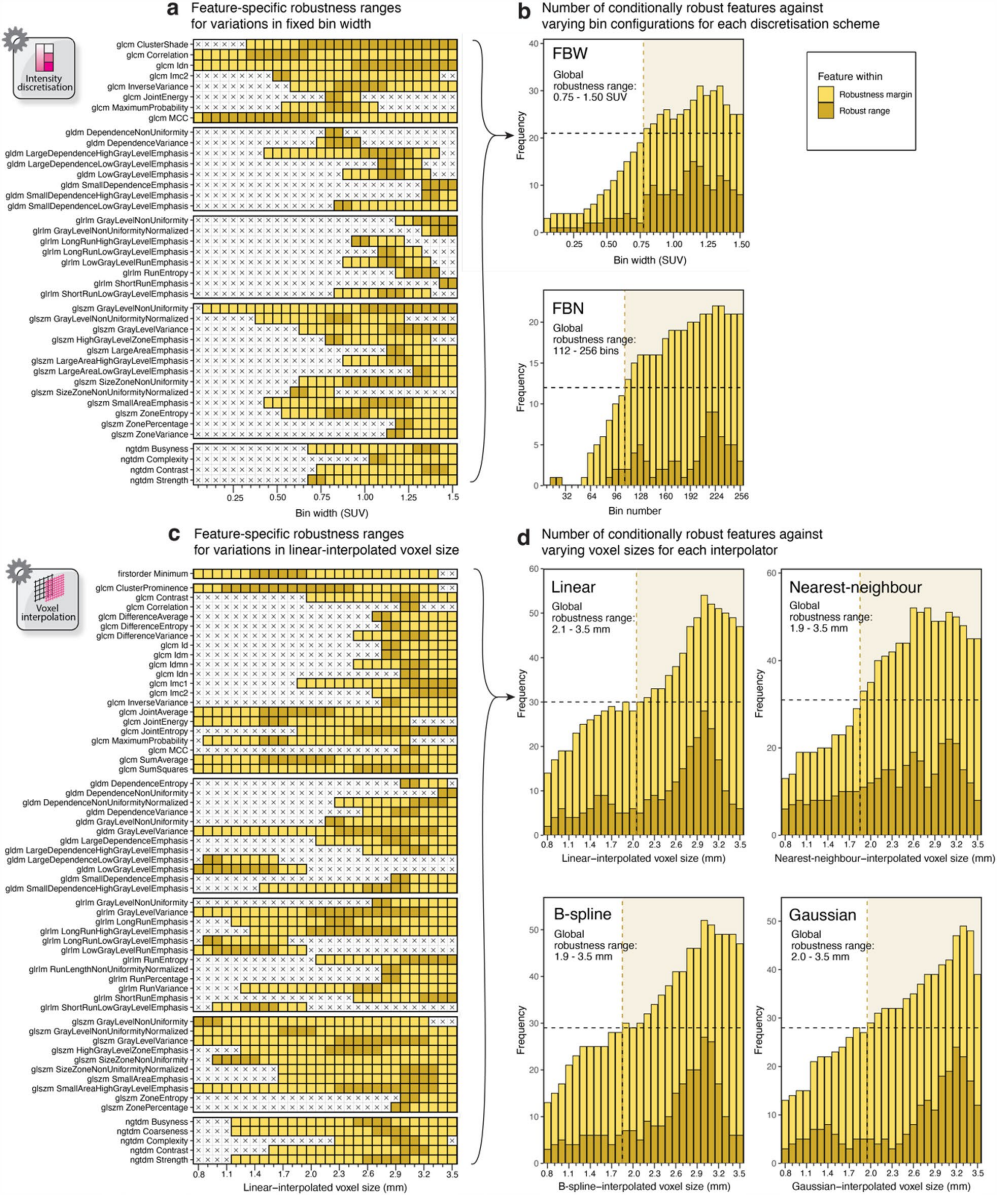
Feature-specific robustness ranges when applying FBW discretisation (**a**), and histograms showing the number of conditionally robust features across different bin configurations for FBW and FBN discretisation (**b**), when NSCLC cases were analysed. Similarly, feature-specific robustness ranges for linear interpolation (**c**), and histograms showing the distribution of conditionally robust features against interpolated voxel size for the four interpolators: linear, nearest-neighbour, B-spline, and Gaussian (**d**). A histogram cut-off recovering at least half of the conditionally robust features (indicated by the horizontal black dashed line) was applied, generating the corresponding global robustness ranges as indicated by the vertical yellow dashed line and annotated in the histograms (**b**,**d**).

Figure 4 provides a summary of the ensemble of [^18^F]-FDG-PET radiomic features determined to be conditionally robust in NSCLC cases, when FBW discretisation or linear interpolation was used. Summary figures for other methods and cancer types are given in Supplementary Fig. SF2–4.

For NSCLC, 41/107 (38%) radiomic features were found to be robust within certain intensity resolutions introduced by FBW discretisation (Fig. 4a). These comprised 8/24 (33%) GLCM, 8/14 (57%) GLDM, 8/16 (50%) GLRLM, 13/16 (81%), GLSZM, and 4/5 NGTDM features. Across families, the robustness ranges exhibited a tendency to encompass larger bin widths, as observed in the frequency histogram in Fig. 4b (top row). A cut-off recovering at least half of the conditionally robust radiomic features (≥ 21 and ≥ 12 features for FBW and FBN, respectively) from the histogram yielded a global robustness range between 0.75 and 1.50 SUV for FBW, and 112 and 256 bins for FBN discretisation (Fig. 4b). Similarly, for the linear interpolation example in Fig. 4c, 60/107 (56%) radiomic features were robust for certain voxel sizes, with more than half (≥ 30) of these features being robust between 2.1 and 3.5 mm (Fig. 4d; top-left). Figure 4d shows the histogram analyses for other interpolators.

The histogram analyses of all discretisation or interpolation schemes across the cancer types considered in this work are provided in Supplementary Fig. SF2–3, and a summary of the resulting global robustness ranges is tabulated in Table 1. In general, there was a substantial overlap in robustness range between cancer types for both discretisation and interpolation, with melanoma exhibiting the widest ranges for the latter. Across interpolators, global robustness ranges tended to encompass larger voxel sizes, with the number of robust features peaking close to the slice thickness of the original voxels (3 mm).

**Table 1:** Global robustness ranges stratified by discretisation or interpolation algorithm and cancer type. The bin configuration(s) or voxel size(s) achieving the highest number of features within the robust or robustness range are given in parentheses.

	NSCLC	Melanoma	Lymphoma
Discretisation			
Fixed bin width (SUV)	0.75–1.50 (1.15|1.20 or 1.35)	0.50–1.50 (1.05|1.05)	0.50–1.50 (1.05|1.05)
Fixed bin number (bins)	112–256 (216 or 224|224 or 232)	112–256 (224|216, 224, or 240)	112–256 (248|248)
Interpolation			
Linear (mm)	2.1–3.5 (3.0|3.0)	1.5–3.5 (3.0|2.8)	1.8–3.5 (2.4 or 3.0|3.0)
Nearest-neighbour (mm)	1.9–3.5 (3.1|2.6 or 2.8)	1.3–3.5 (2.2|3.0)	1.9–3.5 (2.2, 3.0, or 3.2|3.0 or 3.1)
B-spline (mm)	1.9–3.5 (3.0|3.0)	1.5–3.5 (3.1|2.9 or 3.0)	2.0–3.5 (3.0|2.7, 3.0 or 3.1)
Gaussian (mm)	2.0–3.5 (3.2|3.3)	1.5–3.5 (3.1 or 3.2|2.9, 3.0, or 3.1)	2.2–3.5 (3.2|3.2)

### Correctable features

We also explored the feasibility of parameter dependency correction as an alternative strategy to improve the robustness of features initially not meeting the robust criteria.

**Figure 5:**
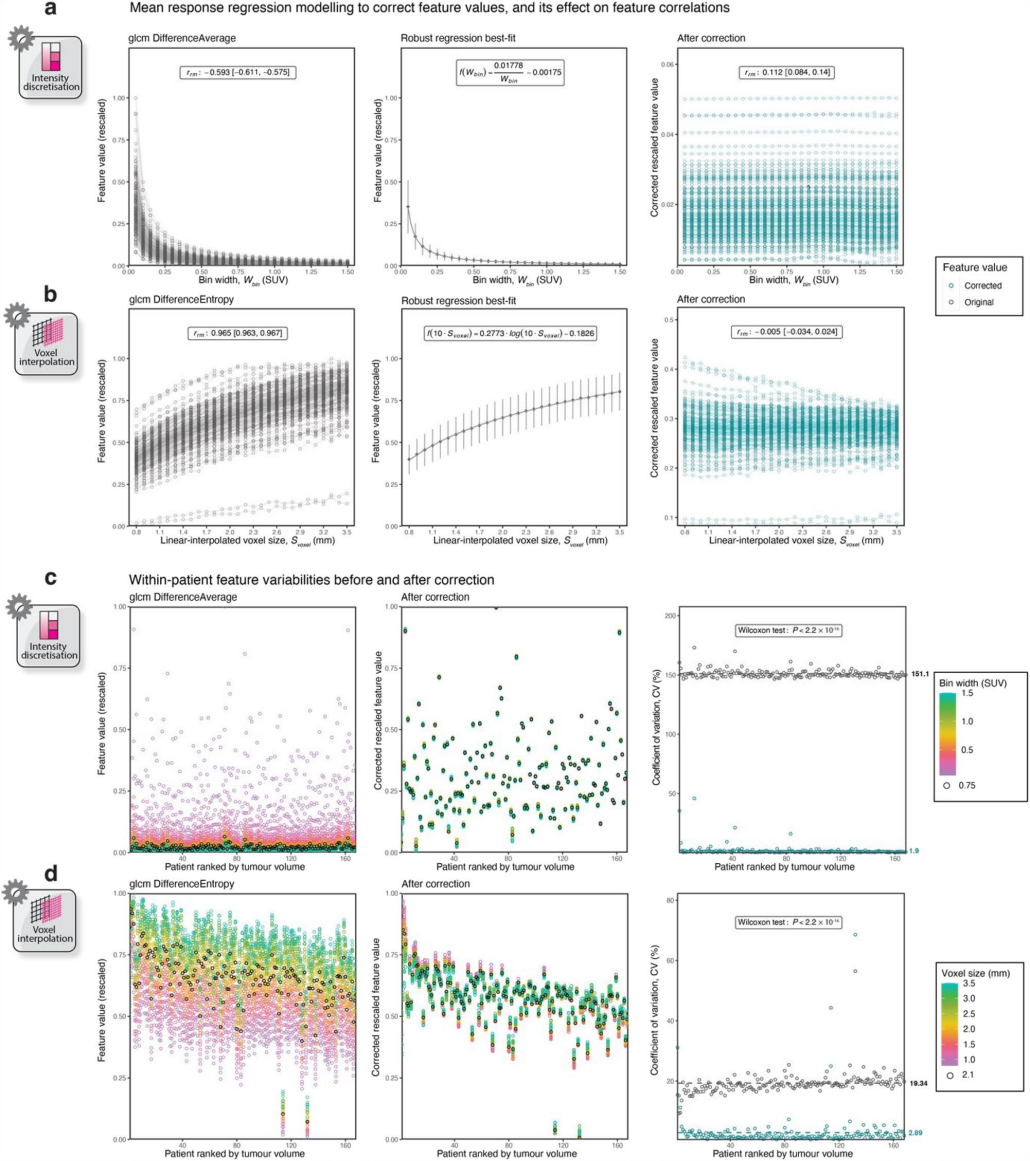
Feature values for GLCM *DifferenceAverage* and *DifferenceEntropy* against bin width (**a**) or linear-interpolated voxel size (**b**), respectively, tracked for every NSCLC patient. Here, original (uncorrected) values are plotted on the left; the best-fit model describing the relationship of the mean feature values as a function of bin width or linear-interpolation voxel size is presented in the centre; and feature values corrected using the inverse function of the best fit are given on the right. Uncorrected and corrected values for the same features are plotted from lowest to highest patient tumour volume, with coloured dots indicating variations in bin width (**c**) or linear-interpolated voxel size (**d**) used during feature extraction. The corresponding per-patient coefficient of variations before and after correction are provided on the right. All feature values have been rescaled using min–max normalisation.

As examples, Fig. 5a,b provide the response to variations in FBW-discretised intensity resolution for GLCM *DifferenceAverage* and linear-interpolated voxel size for GLCM *DifferenceEntropy*, respectively. In these instances, the effect of bin width could be modelled by an inverse function, whereas a logarithmic function was used to model variations in voxel size. These dependencies were correctable by applying the rearranged form of their respective best-fit models, leading to a significant reduction in feature correlation after correction (i.e., |*r*_*rm*_|< 0.5, as annotated in the figures on the right).

Figure 5c,d (left) show original feature values for GLCM *DifferenceAverage* and *DifferenceEntropy*, respectively, for each patient ranked by tumour volume. Following correction (Fig. 5c,d; middle), we observed a statistically significant reduction in feature variability per patient, as evidenced by a decrease in the dispersion of coloured points in the figures. Mean feature variability for the two example cases was < 10%, satisfying the robust criterion for *CV*_*mean*_. As such, these features were deemed correctable.

The original and corrected scatter plots for all features with respect to image processing method and cancer type are deposited in https://github.com/syafiqramlee/robust-radiomics-img-processing. A snapshot of the correctability categorisations has been presented in Supplementary Fig. SF5, with the proportions tabulated in Supplementary Table ST2. For correctable features, the best-fit invertible function utilised for correction in each scenario is provided in Supplementary Fig. SF6.

**Figure 6:**
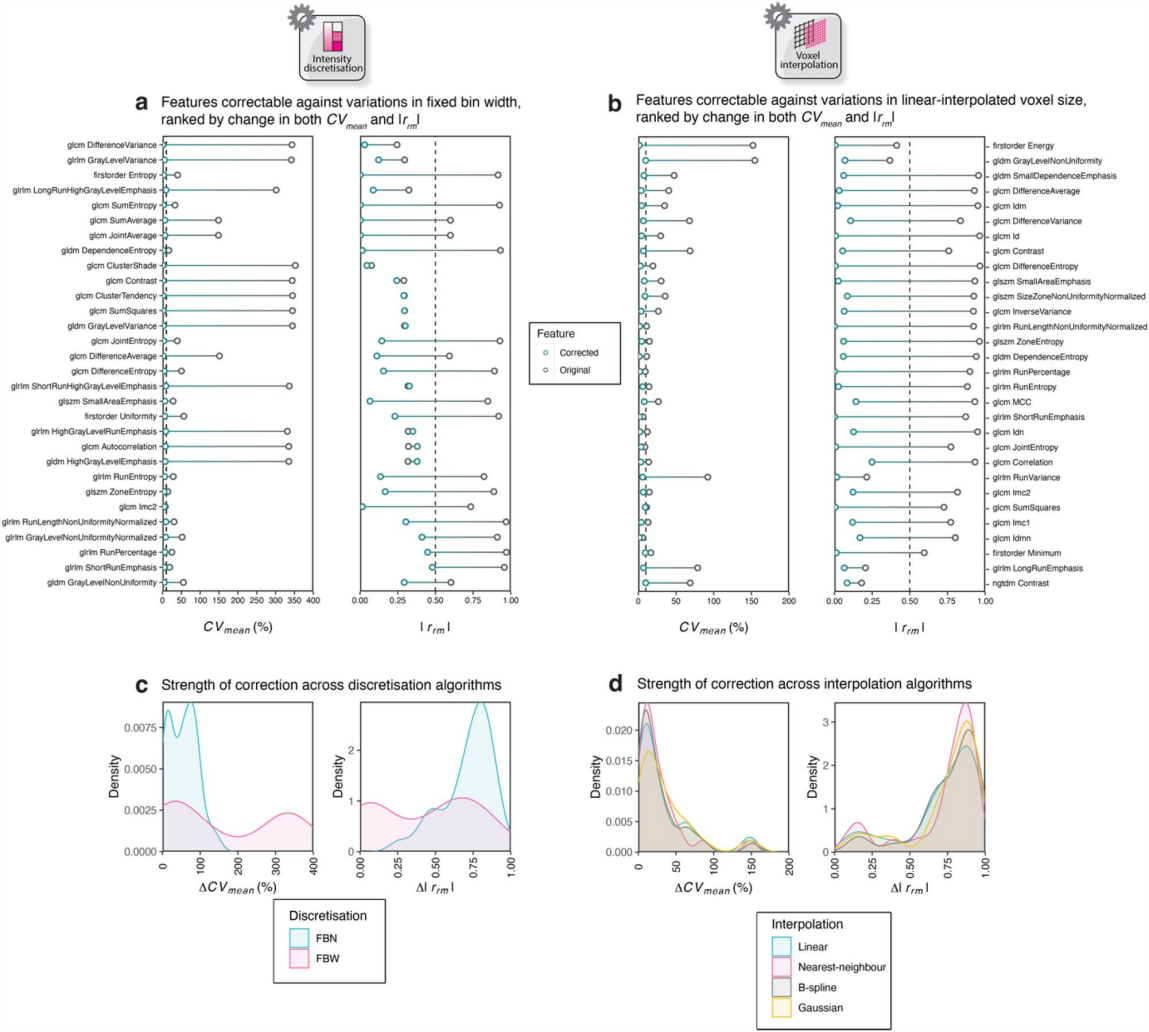
Dumbbell plots showing the change in *CV*_*mean*_ and |*r*_*rm*_| for correctable features when considering FBW discretisation (**a**) and linear interpolation (**b**) in NSCLC. Features are ranked by the change in feature variability and correlation upon correction. Density plots quantifying the overall effect of correction on feature variability and correlation for correctable features with respect to discretisation (**c**) and interpolation (**d**) algorithms.

Figure 6a,b summarise all the features that were correctable in FBW discretisation and linear interpolation scenarios for the NSCLC cohort, ranked by relative change in *CV*_*mean*_ (*ΔCV*) and |*r*_*rm*_| (*Δ|r*_*rm*_*|*). FBN discretisation resulted in a homogeneous reduction in both feature variability and correlation, with a greater reduction in the latter, than FBW (Fig. 6c). The strength of correction was similar across interpolation methods (Fig. 6d). Summary figures for other methods and cancer types can be found in Supplementary Fig. SF7–10, with the most correctable features presented in Supplementary Table ST3.

### Feature categorisations across methods and cancer types

**Figure 7:**
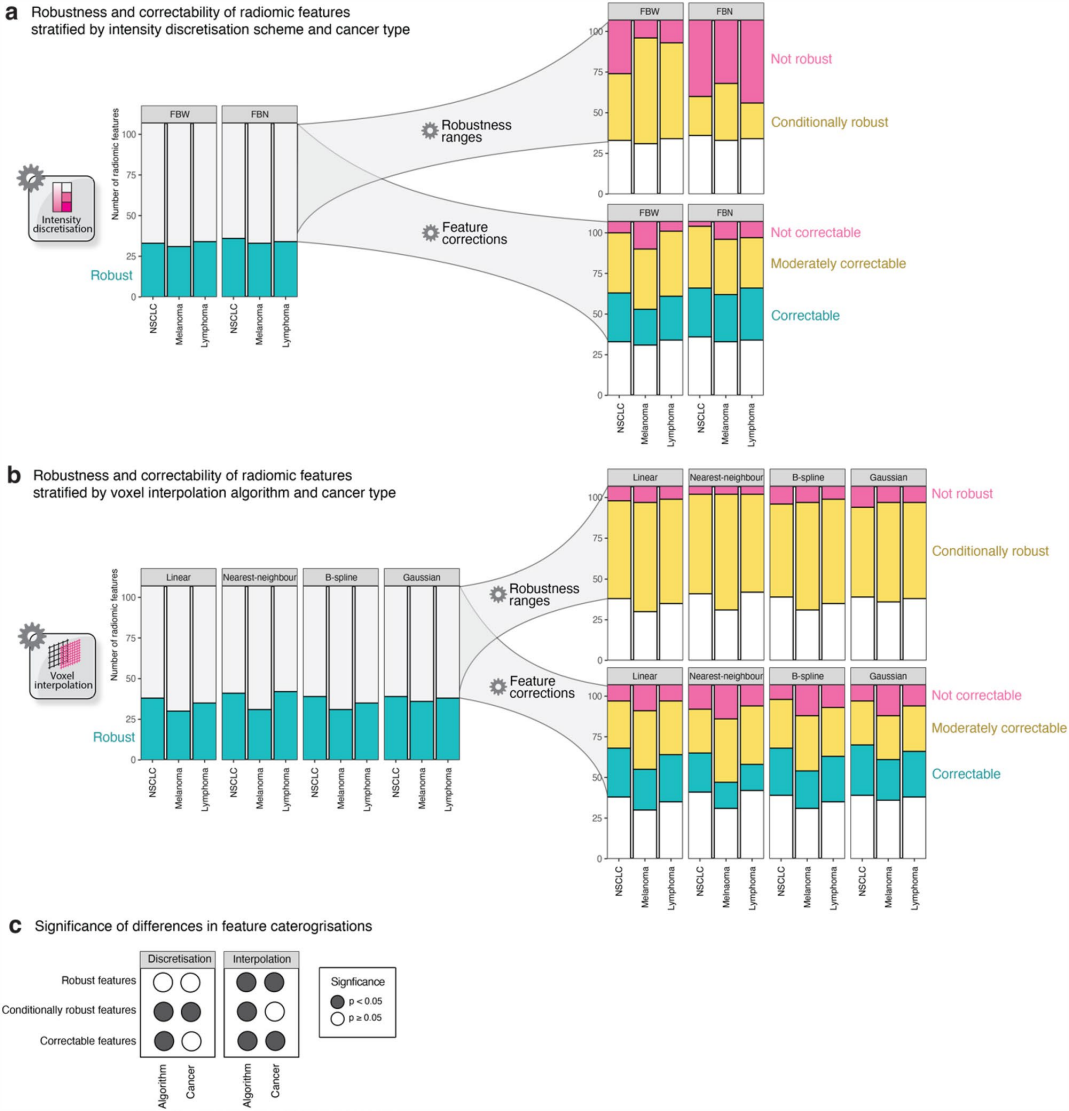
[^18^F]-FDG-PET radiomic feature robustness and correctability categorisations stratified by algorithm and cancer type for intensity discretisation (**a**) and voxel interpolation (**b**), and their corresponding significance of differences (**c**).

Figure 7 aggregates robustness and correctability categorisations of [^18^F]-FDG-PET radiomic features stratified by image processing method and cancer type.

FBW discretisation tended to produce more conditionally robust radiomic features than FBN (p < 0.001), especially for melanoma (p < 0.001), lymphoma (p < 0.001), and less so for NSCLC (p = 0.05) (Fig. 7a). The effect of cancer type on conditional feature robustness for both discretisation methods was overall significant (p < 0.001), with melanoma exhibiting the highest number of conditionally robust features (Supplementary Table ST1). Nearest-neighbour interpolation produced more conditionally robust features than linear (p = 0.015), Gaussian (p < 0.001), and B-spline (p < 0.004), after correction for multiple comparisons. The impact of cancer type on conditional feature robustness for interpolation was not statistically significant (p = 0.3), with melanoma resulting in the highest number of conditionally robust features (Supplementary Table ST1).

Feature correctability significantly depended on the discretisation algorithm (p = 0.045), with FBN resulting in more correctable features than FBW discretisation (Supplementary Table ST2). While melanoma yielded the fewest correctable features for both discretisation schemes (Supplementary Table ST2), the effect of cancer type on correctability was not significant (p = 0.09). In contrast, feature correctability was impacted by both interpolation algorithm (p < 0.001) and cancer type (p < 0.001), with nearest-neighbour interpolation or melanoma resulting in the least number of correctable features (Supplementary Table ST2).

## Discussion

Radiomic features may convey clinically meaningful information about tumours but can be susceptible to variations in factors pertaining to image acquisition, reconstruction, and processing^19,40^. In this work, we performed an in-depth characterisation of the robustness of [^18^F]-FDG-PET radiomic features to variations in bin configuration or voxel size introduced by discretisation and interpolation, respectively, across a cohort of three cancer types (NSCLC, melanoma, and lymphoma). Features not meeting our robust criteria were analysed to ascertain if their robustness could be improved by imposing parameter range conditions or dependency corrections. To our knowledge, our study is the first to collectively evaluate the sensitivity, robustness ranges, and mathematical correctability of tumour [^18^F]-FDG-PET radiomic features against variations in bin configuration or voxel size.

### Impact of intensity discretisation and voxel interpolation on [^18^F]-FDG-PET radiomic feature robustness

We showed that radiomic features were significantly more sensitive to intensity discretisation than voxel interpolation, with second-order features manifesting the least stability to variations in intensity bin configuration or voxel size. These observations concur with prior reports^9,16,41^, as these textural features take into account the spatial relationship between voxel intensities. This is further exemplified by our findings that GLCM *idmn* and GLCM *SumEntropy* were the only second-order features found to be robust across all our discretisation and interpolation investigations, respectively. Such features were also highlighted as robust in other studies^10,17,42^, potentiating their utility as suitable radiomic candidates for reliable comparison between images for which different bin configurations or voxel sizes are used.

When comparing intensity discretisation algorithms used in radiomics studies, we found feature values displayed a higher tolerance to changes in bin width than bin number, as reflected by the greater number of conditionally robust features for the former. This agrees with existing studies supporting the use of FBW discretisation in radiomic interrogations^10,27,43–45^, given this scheme preserves the relationship between SUV units and discretised intensity levels^46^. However, it should be noted that FBW discretisation may introduce variations in the number of bins between tumour volumes, and should not be advocated in all use-cases as features may become non-informative or incomparable where significant inter-lesion heterogeneity is observed^23,43^. To this end, some authors have found the use of FBN discretisation to be advantageous as it shows little dependence on the SUV, reconstruction parameters, and noise^47^.

The choice of interpolation algorithm on [^18^F]-FDG-PET radiomics analyses should be carefully considered as we observed a significant dependency of feature robustness on the type of interpolator. Images resampled using nearest-neighbour interpolation generated the greatest number of robust features than other interpolators considered in this work (i.e., linear, B-spline, and Gaussian). This observation is concordant with an earlier study^48^, and was also found when we compared the conditional robustness of features. A plausible explanation for the high tolerance of radiomic features to variable voxel sizes when considering nearest-neighbour interpolation could be that such an interpolation method preserves the intensity spectrum of the original data^49^.

### Mitigating the impact of image processing variations on radiomic features using robustness ranges

Given that features not satisfying the robust criteria may exhibit varying sensitivity across the image processing parameters tested, we hypothesised that these sensitivities could be attenuated by restricting radiomic interrogations to within certain parameter ranges. This hypothesis was confirmed for most of our investigations, where the majority of these radiomic features could be recovered by applying parameter range conditions for bin configuration or voxel size.

Robustness range conditions were derived on a per-feature or all-features (global) basis for each algorithm and cancer type. Our results indicated robustness ranges for the former varied considerably, even in the same feature family, suggesting that the feature-specific response to discretisation or interpolation is an important aspect to consider when selecting features for radiomics analyses^50,51^. Nevertheless, we envisage that global robustness ranges could be helpful in choosing an optimal bin configuration or interpolated voxel size, which are usually required as input parameters for radiomic feature computations.

Global robustness ranges for the discretisation methods tested (FBW: 0.5 to 1.50 SUV; FBN: 112 to 256 bins) both favoured the use of larger values for improved feature robustness, with the upper limits being the largest values tested. This indicates that although the width and number of bins are inversely related properties, their overall impact on robustness across tumours may not always be inversely related. For FBW discretisation, this may be explained by a reduction in image noise, increased clustering of similar intensity levels or, more severely, potential loss of information as larger bin widths are used^23,43^. Therefore, to preserve meaningful comparisons while still recovering the overall robustness of sensitive features, smaller bin widths in the global robustness range should take precedence (e.g., 0.5 SUV). On the other hand, the improvement of feature robustness as we increase the number of bins in FBN discretisation could be attributed to the large voxel sizes commonly found in PET images, where the effect of adding bins on the appearance of tumour volumes may be constrained by the limited number of voxel intensities assessed.

Across interpolators, global robustness ranges for voxel size were markedly similar, and widest for nearest-neighbour interpolation in melanoma (1.3–3.5 mm). Analysed images had an original voxel dimension of 2.03 × 2.03 × 3 mm^3^, which we resampled into a range of isotropic voxel sizes to ensure rotationally invariant feature values, as recommended by IBSI^26^. We observed that the number of features robust within certain voxel sizes peaked when voxels were interpolated to an isotropic dimension equal, or close to, the slice thickness. This may highlight the critical role of the largest voxel dimension on the interpolated value of voxels and robustness of features, and echoes previous findings in a magnetic resonance imaging study^52^.

### Improving feature robustness to image processing variations using feature correction

Aside from determining robustness ranges, which may be circumscribed by the fact that only a subset of bin configurations or voxel sizes could be used, we explored feature correction as an alternative strategy to achieve robustness. To this end, the underlying dependency of [^18^F]-FDG-PET radiomic features non-robust to variations in bin configuration and voxel sizes was modelled and corrected using a library of eight invertible functions, as utilised in previous work^23^. This approach has been shown to be software-agnostic and feature definition non-specific^23^.

While we found that not all features were correctable to discretisation and interpolation, approximately a third of second-order non-robust features could be corrected. As prime examples, *Entropy* for discretisation and *Energy* for interpolation could not meet the robust criteria in all tested instances but were ranked amongst the most correctable using basic mathematical functions in our results. In a conventional radiomics modelling study, these potentially meaningful but non-robust features would have been pruned during feature selection; yet, applying corrections with simple equations has demonstrated that these features could have easily been preserved. However, we highlight that feature corrections exhibited limited generalisability, as there was a notable dependency on the image processing method used, with a lower number of features being correctable than conditionally robust.

Still, our results encourage the possibility of incorporating bin configurations or voxel sizes into existing definitions of radiomic features^27^, as also asserted in earlier investigations^17,21,22^. An alternative to the use of explicitly defined mathematical equations, which we considered in this work, would have been to include feature-specific variations to discretisation or interpolation within the construct of a mixed-effects model. To this end, a similar approach has been proposed for reducing the variability of radiomic features before pooling data from different sites^53^.

### Effect of cancer type on feature robustness and correctability

When comparing cancer types, melanoma images consistently generated the fewest robust or correctable [^18^F]-FDG-PET radiomic features, with the effect of cancer type on robustness being significant when considering variations in voxel size. We postulate these observations could be owed to the considerably smaller analysed tumour volumes for melanoma cases, given prior evidence that radiomic features can exhibit volume dependence^23,24,54^. Nonetheless, melanoma cases demonstrated a significantly greater number of features conditionally robust to bin configurations, and the widest global robustness ranges for resampled voxel sizes. Therefore, robustness can still be achieved by using common bin configurations or resampling images to appropriate voxel sizes.

### Limitations

This study carries several limitations. First, while we investigated image processing parameters covering those found in the literature, these parameters are not exhaustive (e.g., voxel sizes smaller than 0.8 mm were not investigated). However, we used an extensive set of image processing parameter configurations (e.g., intensity bin numbers as multiples of 8 instead of powers of 2) to thoroughly explore the robustness of features extracted from [^18^F]-FDG-PET images. Second, for feature correction, we tested the fits of only eight functions, and more reliable fits and better correction could possibly be achieved by considering a larger library of functions or implementing these equations in a piecewise manner. Third, tumour segmentations for each patient were treated as a single volumetric mask, even in cases where multiple lesions were present. While this approach is not atypical for radiomics studies^55–58^, it should be noted that the goal of this work was not to correlate these features with lesion-specific clinicopathological characteristics. Furthermore, lesion-by-lesion analyses would have increased the complexity of radiomic computations because of the inherently smaller volumes. Finally, the generalisability of the findings from this work warrants further investigation using data involving other cancer types and imaging protocols, ideally acquired in a prospective manner. These studies may also consider examining the combined effect of intensity discretisation and voxel interpolation on [^18^F]-FDG-PET features, alongside variations in other factors known to influence radiomics, such as lesion segmentation (e.g., intra- and inter-reader variability) and acquisition or reconstruction settings^19,40,59^. Nevertheless, our use of a large, multi-cancer, and homogeneously acquired imaging dataset allowed us to examine the aims of this study with better statistical power.

## Conclusions

In conclusion, [^18^F]-FDG-PET radiomic features were more sensitive to intensity discretisation than voxel interpolation, especially for second-order families, with feature robustness also exhibiting a potential cancer type dependence. Non-robustness of features could be addressed by implementing range conditions or dependency corrections on bin configuration or voxel size, with both approaches significantly affected by the choice of discretisation or interpolation algorithm used. Robustness ranges were suitable for most features tested and may inform prospective study designs on which bin configurations or voxel sizes would be optimal; however, this approach could be restrictive as only parameters within this range can be selected. In contrast, feature corrections using mathematical functions work reasonably well across the entire spectrum of parameters tested and may be valuable when pooling multi-centric heterogeneous datasets, albeit this approach is appropriate for less features. Taken together, the results of this work offer a more nuanced understanding of the impact of image processing variations on [^18^F]-FDG-PET radiomic features, and underscore the importance of selecting reliable imaging biomarkers for clinical use.

### Supplementary Information

Supplementary Information.

## Data Availability

The imaging dataset used for this study are publicly available at The Cancer Imaging Archive, as referenced in the article. All other data generated and/or analysed during this study are included in this article, its supplementary materials, and a GitHub repository at https://github.com/syafiqramlee/robust-radiomics-img-processing.
