# Supplementary material for: Mitigating the impact of image processing variations on tumour [18F]-FDG-PET radiomic feature robustness
Source: Sci Rep. 2024 Jul 15;14:16294. doi: 10.1038/s41598-024-67239-8 (PMC11251269; doi:10.1038/s41598-024-67239-8)

Supplementary materials

Mitigating the impact of image processing variations on tumour [^{18}F]-FDG-PET radiomic feature robustness

Syafiq Ramlee *, Roido Manavaki, Luigi Aloj, Lorena Escudero Sanchez

* Correspondence: Syafiq Ramlee, masr4@cam.ac.uk

Supplementary Table ST1

The number of features (and corresponding percentage proportion, out of the 107 features extracted) for each robustness category, stratified by intensity discretisation or voxel interpolation scheme and cancer type.

	Robust			Conditionally robust			Not robust		
	NSCLC	Melanoma	Lymphoma	NSCLC	Melanoma	Lymphoma	NSCLC	Melanoma	Lymphoma
**Discretisation**									
FBW	33 (30%)	31 (29%)	34 (32%)	41 (38%)	65 (61%)	59 (55%)	33 (31%)	11 (10%)	14 (13%)
FBN	36 (34%)	33 (31%)	34 (32%)	24 (22%)	35 (33%)	22 (21%)	47 (44%)	39 (36%)	51 (48%)
**Interpolation**									
Linear	38 (36%)	30 (28%)	35 (33%)	60 (56%)	67 (63%)	64 (60%)	9 (8%)	10 (9%)	8 (7%)
Nearest-neighbour	41 (38%)	31 (29%)	42 (39%)	61 (57%)	71 (66%)	60 (56%)	5 (5%)	5 (5%)	5 (5%)
B-spline	39 (36%)	31 (29%)	35 (33%)	57 (53%)	66 (62%)	64 (60%)	11 (10%)	10 (9%)	8 (7%)
Gaussian	39 (36%)	36 (34%)	38 (36%)	55 (51%)	61 (57%)	59 (55%)	13 (12%)	10 (9%)	10 (9%)

Supplementary Table ST2

The number of features (and corresponding percentage proportion, out of the 107 features extracted) for each correctability category, stratified by intensity discretisation or voxel interpolation scheme and cancer type.

	Correctable			Moderately correctable			Not correctable		
	**NSCLC**	**Melanoma**	**Lymphoma**	**NSCLC**	**Melanoma**	**Lymphoma**	**NSCLC**	**Melanoma**	**Lymphoma**
**Discretisation**									
FBW	30 (28%)	22 (20%)	27 (25%)	37 (35%)	37 (35%)	40 (37%)	7 (7%)	17 (16%)	6 (6%)
FBN	30 (28%)	29 (27%)	32 (30%)	38 (36%)	34 (32%)	31 (29%)	3 (3%)	11 (10%)	10 (9%)
**Interpolation**									
Linear	30 (28%)	25 (23%)	29 (27%)	29 (27%)	36 (34%)	33 (30%)	10 (9%)	16 (15%)	10 (9%)
Nearest-neighbour	24 (22%)	16 (15%)	16 (15%)	27 (25%)	39 (36%)	36 (34%)	15 (14%)	21 (20%)	13 (12%)
B-spline	29 (27%)	23 (22%)	28 (26%)	30 (28%)	34 (32%)	30 (28%)	9 (8%)	19 (18%)	14 (13%)
Gaussian	31 (29%)	25 (23%)	28 (26%)	27 (25%)	27 (25%)	28 (26%)	10 (9%)	19 (18%)	13 (12%)

Supplementary Table ST3

The most correctable feature for each intensity discretisation or voxel interpolation scheme, across the cancers investigated.

	NSCLC	Melanoma	Lymphoma
**Discretisation**			
FBW	GLCM DifferenceVariance	GLCM Contrast	GLCM Contrast
FBN	First-order Uniformity	GLCM ClusterTendency	First-order Uniformity
**Interpolation**			
Linear	First-order Energy	GLSZM ZonePercentage	First-order Energy
Nearest-neighbour	First-order Energy	First-order Energy	First-order Energy
B-spline	First-order Energy	GLSZM ZonePercentage	First-order Energy
Gaussian	First-order Energy	GLSZM ZonePercentage	First-order Energy

Supplementary Figure SF1

Robustness categorisations mosaic for the 107 radiomic features tested, stratified by image processing algorithm and cancer type.

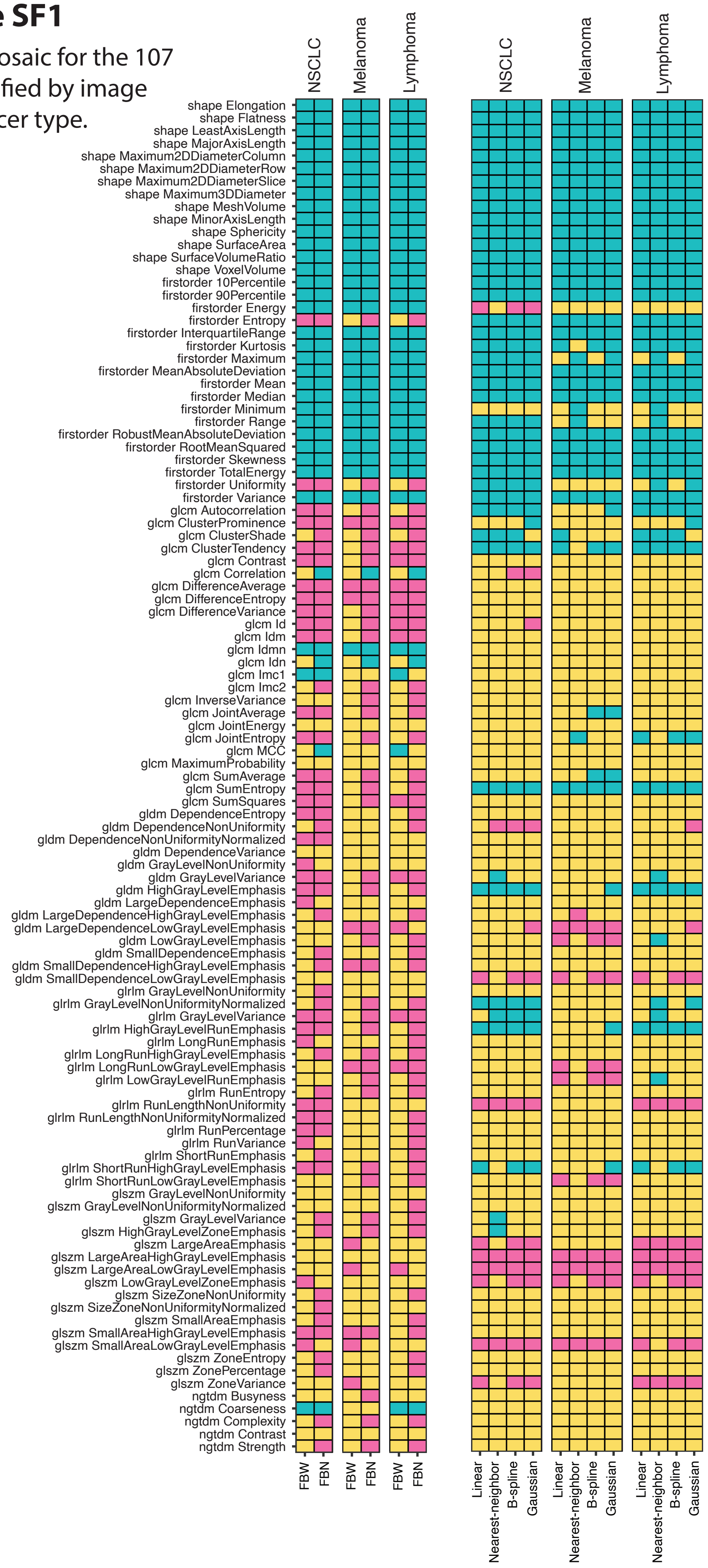

Supplementary Figure SF2

Global and feature-specific robustness ranges of conditionally robust features in NSCLC, for each discretisation (a-b) or interpolation (c-f) algorithm.

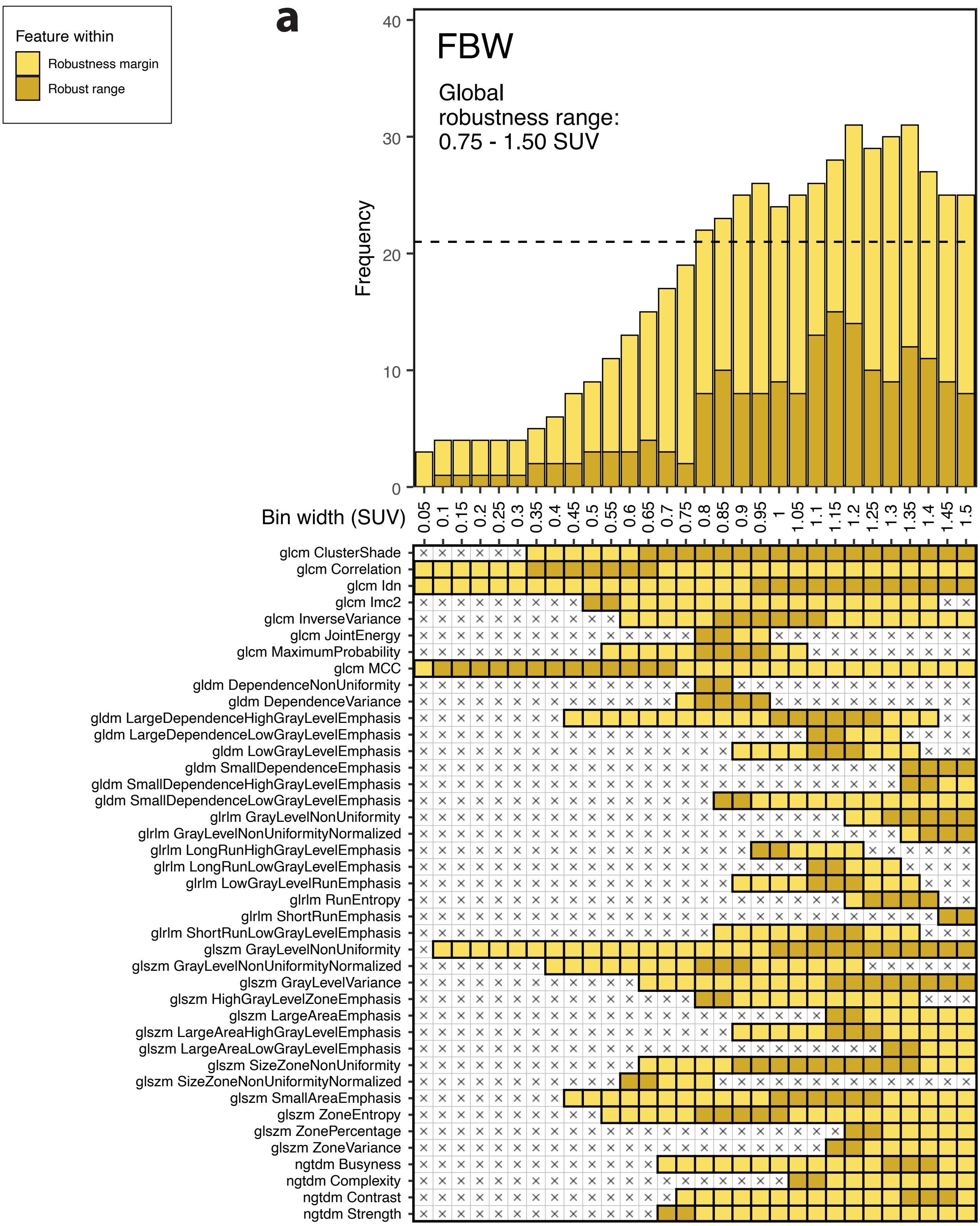

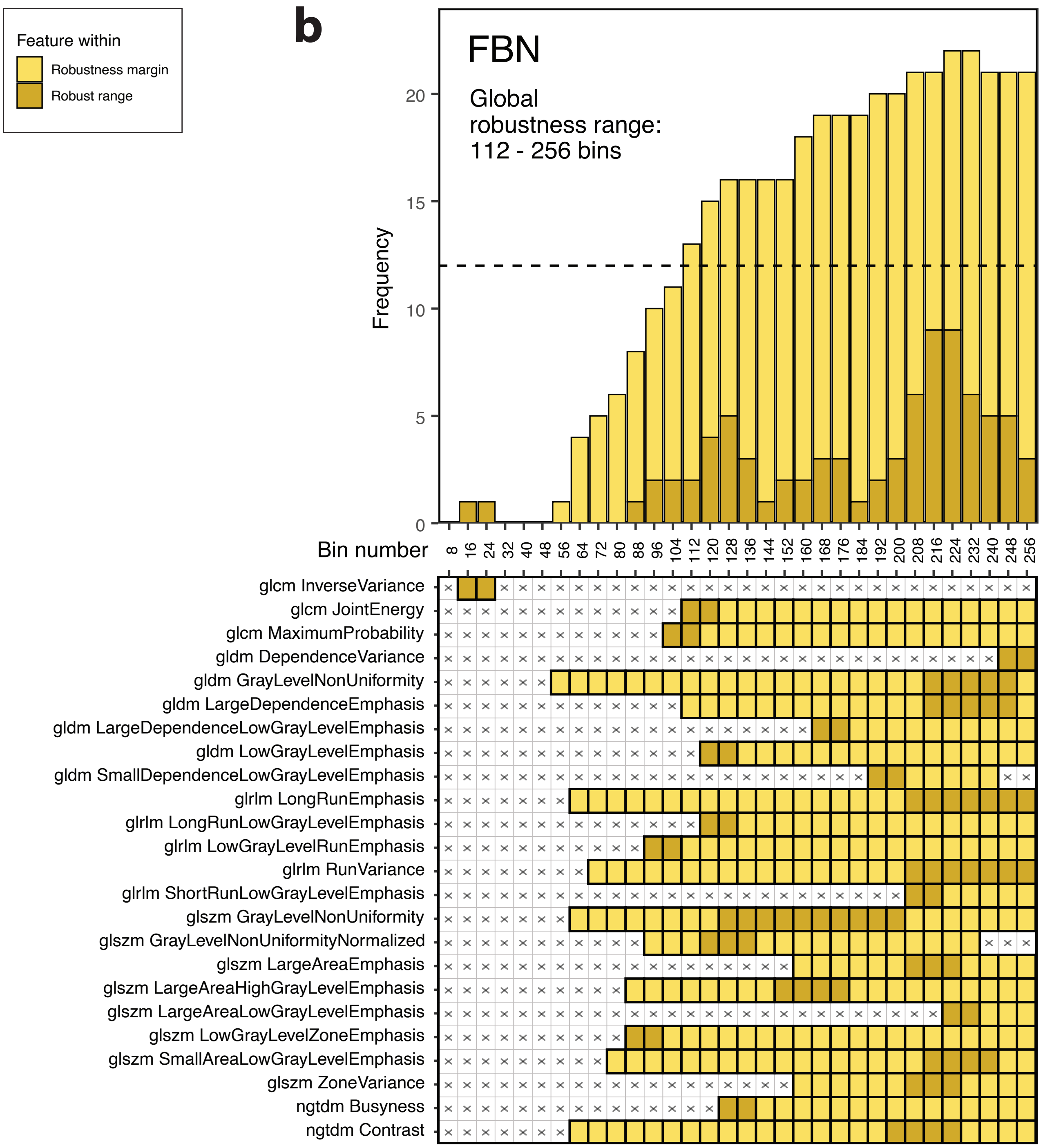

Feature within
Robustness margin
Robust range
b
FBN
Global
robustness range:
112 - 256 bins
Frequency
Bin number
glcm InverseVariance
glcm JointEnergy
glcm MaximumProbability
gldm DependenceVariance
gldm GrayLevelNonUniformity
gldm LargeDependenceEmphasis
gldm LargeDependenceLowGrayLevelEmphasis
gldm LowGrayLevelEmphasis
gldm SmallDependenceLowGrayLevelEmphasis
glrlm LongRunEmphasis
glrlm LongRunLowGrayLevelEmphasis
glrlm LowGrayLevelRunEmphasis
glrlm RunVariance
glrlm ShortRunLowGrayLevelEmphasis
glszm GrayLevelNonUniformity
glszm GrayLevelNonUniformityNormalized
glszm LargeAreaEmphasis
glszm LargeAreaHighGrayLevelEmphasis
glszm LargeAreaLowGrayLevelEmphasis
glszm LowGrayLevelZoneEmphasis
glszm SmallAreaLowGrayLevelEmphasis
glszm ZoneVariance
ngtdm Busyness
ngtdm Contrast

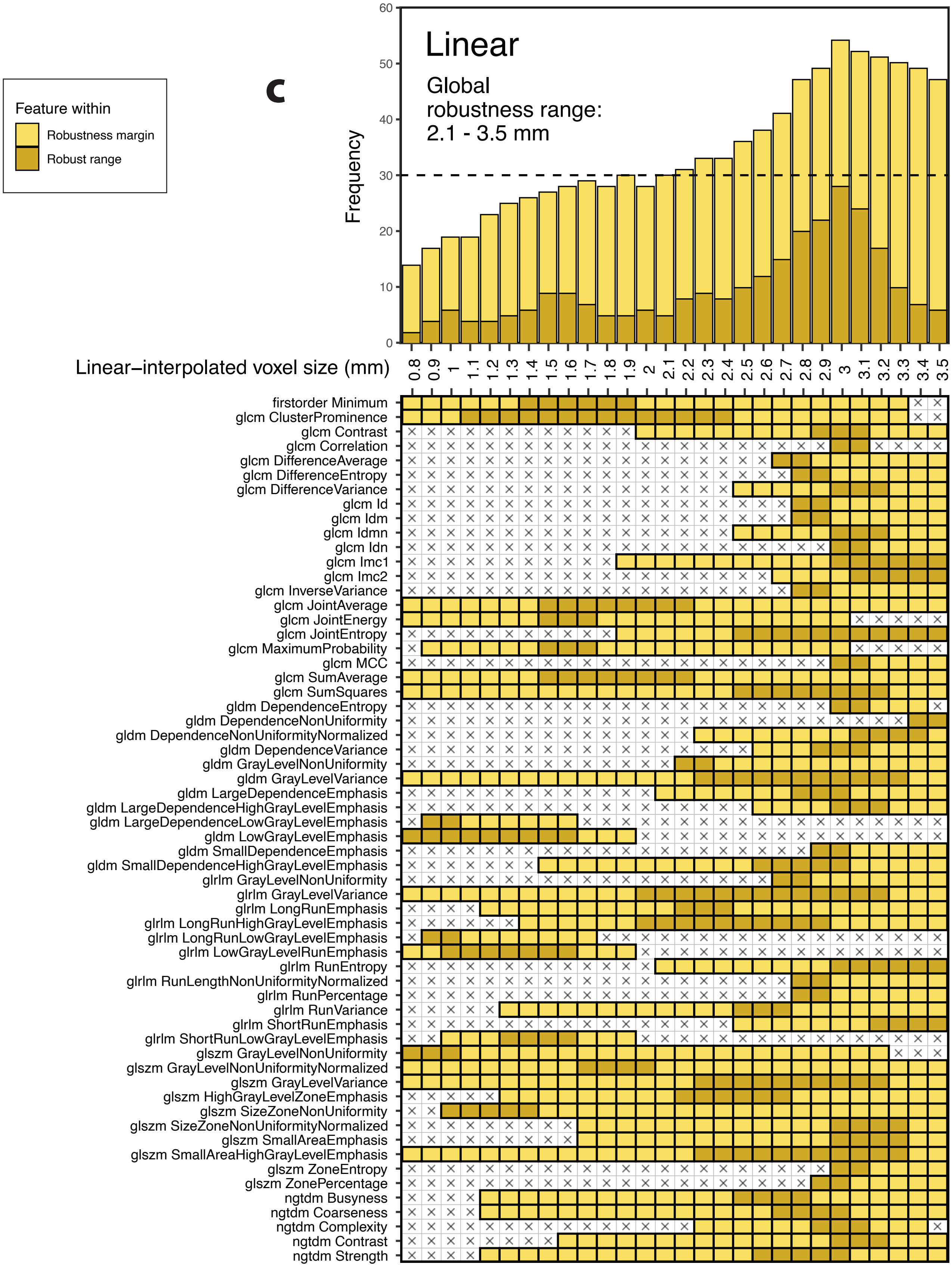
c
Feature within
Robustness margin
Robust range
Linear
Global
robustness range:
2.1 - 3.5 mm
Frequency
0
10
20
30
40
50
60
Linear–interpolated voxel size (mm)
0.8
0.9
1
1.1
1.2
1.3
1.4
1.5
1.6
1.7
1.8
1.9
2
2.1
2.2
2.3
2.4
2.5
2.6
2.7
2.8
2.9
3
3.1
3.2
3.3
3.4
3.5
firstorder Minimum
glcm ClusterProminence
glcm Contrast
glcm Correlation
glcm DifferenceAverage
glcm DifferenceEntropy
glcm DifferenceVariance
glcm Id
glcm Idm
glcm Idmn
glcm Idn
glcm Imc1
glcm Imc2
glcm InverseVariance
glcm JointAverage
glcm JointEnergy
glcm JointEntropy
glcm MaximumProbability
glcm MCC
glcm SumAverage
glcm SumSquares
gldm DependenceEntropy
gldm DependenceNonUniformity
gldm DependenceNonUniformityNormalized
gldm DependenceVariance
gldm GrayLevelNonUniformity
gldm GrayLevelVariance
gldm LargeDependenceEmphasis
gldm LargeDependenceHighGrayLevelEmphasis
gldm LargeDependenceLowGrayLevelEmphasis
gldm LowGrayLevelEmphasis
gldm SmallDependenceEmphasis
gldm SmallDependenceHighGrayLevelEmphasis
glrlm GrayLevelNonUniformity
glrlm GrayLevelVariance
glrlm LongRunEmphasis
glrlm LongRunHighGrayLevelEmphasis
glrlm LongRunLowGrayLevelEmphasis
glrlm LowGrayLevelRunEmphasis
glrlm RunEntropy
glrlm RunLengthNonUniformityNormalized
glrlm RunPercentage
glrlm RunVariance
glrlm ShortRunEmphasis
glrlm ShortRunLowGrayLevelEmphasis
glszm GrayLevelNonUniformity
glszm GrayLevelNonUniformityNormalized
glszm GrayLevelVariance
glszm HighGrayLevelZoneEmphasis
glszm SizeZoneNonUniformity
glszm SizeZoneNonUniformityNormalized
glszm SmallAreaEmphasis
glszm SmallAreaHighGrayLevelEmphasis
glszm ZoneEntropy
glszm ZonePercentage
ngtdm Busyness
ngtdm Coarseness
ngtdm Complexity
ngtdm Contrast
ngtdm Strength

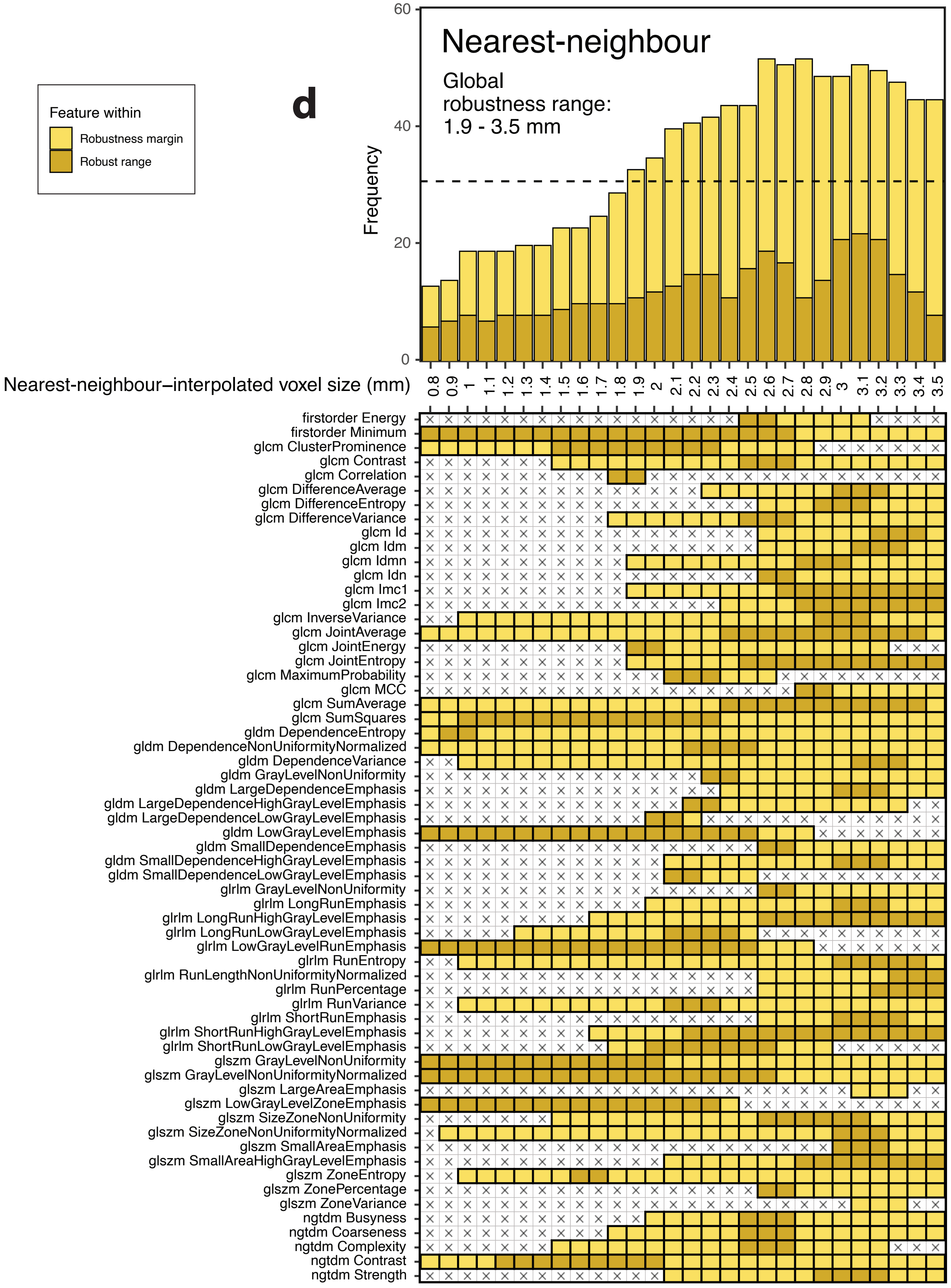
Feature within
Robustness margin
Robust range
d
Nearest-neighbour
Global
robustness range:
1.9 - 3.5 mm
Frequency
0
20
40
60
Nearest-neighbour–interpolated voxel size (mm)
0.8
0.9
1
1.1
1.2
1.3
1.4
1.5
1.6
1.7
1.8
1.9
2
2.1
2.2
2.3
2.4
2.5
2.6
2.7
2.8
2.9
3
3.1
3.2
3.3
3.4
3.5
firstorder Energy
firstorder Minimum
glcm ClusterProminence
glcm Contrast
glcm Correlation
glcm DifferenceAverage
glcm DifferenceEntropy
glcm DifferenceVariance
glcm Id
glcm Idm
glcm Idmn
glcm Idn
glcm Imc1
glcm Imc2
glcm InverseVariance
glcm JointAverage
glcm JointEnergy
glcm JointEntropy
glcm MaximumProbability
glcm MCC
glcm SumAverage
glcm SumSquares
gldm DependenceEntropy
gldm DependenceNonUniformityNormalized
gldm DependenceVariance
gldm GrayLevelNonUniformity
gldm LargeDependenceEmphasis
gldm LargeDependenceHighGrayLevelEmphasis
gldm LargeDependenceLowGrayLevelEmphasis
gldm LowGrayLevelEmphasis
gldm SmallDependenceEmphasis
gldm SmallDependenceHighGrayLevelEmphasis
gldm SmallDependenceLowGrayLevelEmphasis
glrlm GrayLevelNonUniformity
glrlm LongRunEmphasis
glrlm LongRunHighGrayLevelEmphasis
glrlm LongRunLowGrayLevelEmphasis
glrlm LowGrayLevelRunEmphasis
glrlm RunEntropy
glrlm RunLengthNonUniformityNormalized
glrlm RunPercentage
glrlm RunVariance
glrlm ShortRunEmphasis
glrlm ShortRunHighGrayLevelEmphasis
glrlm ShortRunLowGrayLevelEmphasis
glszm GrayLevelNonUniformity
glszm GrayLevelNonUniformityNormalized
glszm LargeAreaEmphasis
glszm LowGrayLevelZoneEmphasis
glszm SizeZoneNonUniformity
glszm SizeZoneNonUniformityNormalized
glszm SmallAreaEmphasis
glszm SmallAreaHighGrayLevelEmphasis
glszm ZoneEntropy
glszm ZonePercentage
glszm ZoneVariance
ngtdm Busyness
ngtdm Coarseness
ngtdm Complexity
ngtdm Contrast
ngtdm Strength

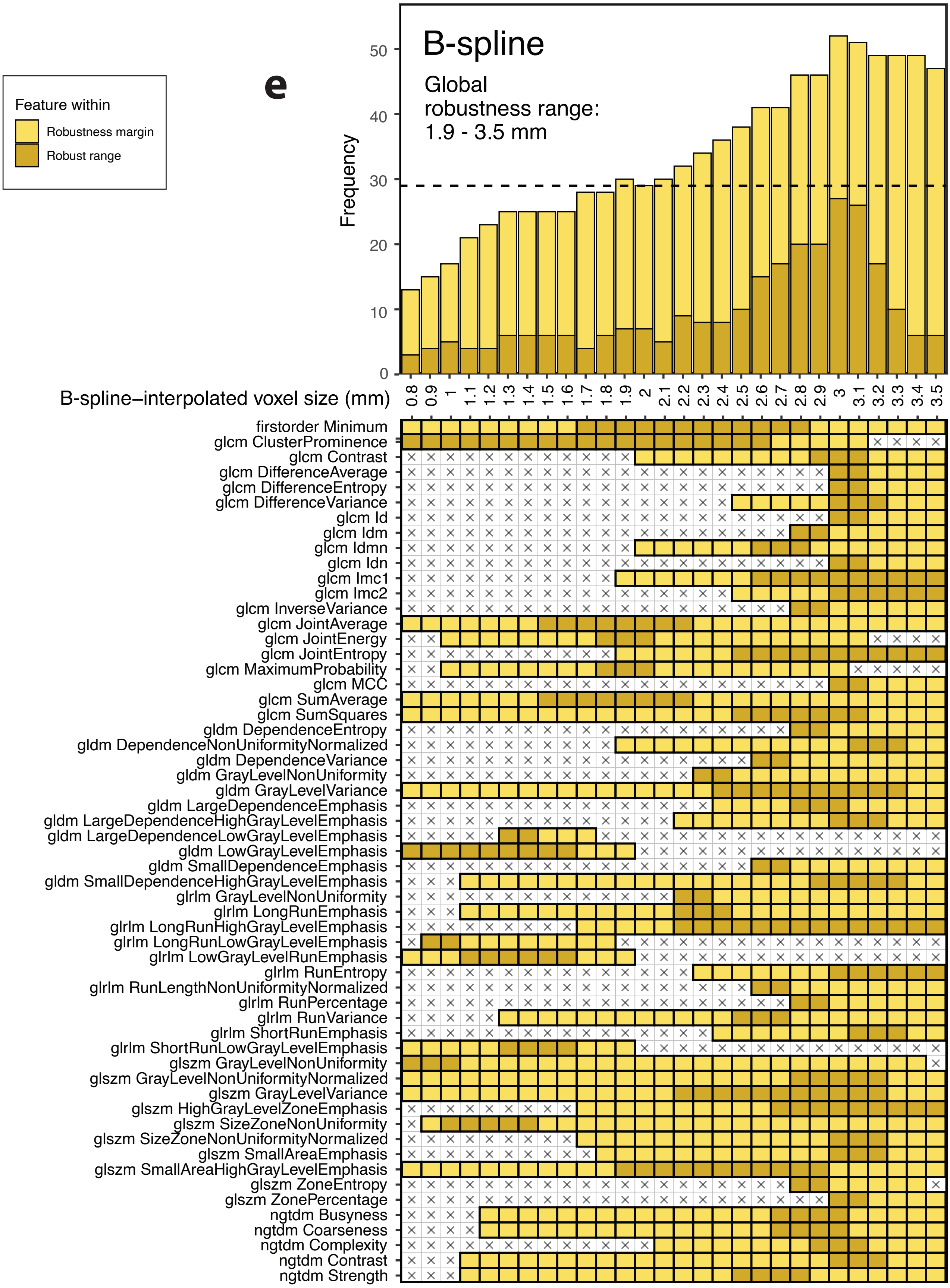
Feature within
Robustness margin
Robust range
e
B-spline
Global robustness range: 1.9 - 3.5 mm
Frequency
B-spline–interpolated voxel size (mm)
firstorder Minimum
glcm ClusterProminence
glcm Contrast
glcm DifferenceAverage
glcm DifferenceEntropy
glcm DifferenceVariance
glcm Id
glcm Idm
glcm Idmn
glcm Idn
glcm Imc1
glcm Imc2
glcm InverseVariance
glcm JointAverage
glcm JointEnergy
glcm JointEntropy
glcm MaximumProbability
glcm MCC
glcm SumAverage
glcm SumSquares
gldm DependenceEntropy
gldm DependenceNonUniformityNormalized
gldm DependenceVariance
gldm GrayLevelNonUniformity
gldm GrayLevelVariance
gldm LargeDependenceEmphasis
gldm LargeDependenceHighGrayLevelEmphasis
gldm LargeDependenceLowGrayLevelEmphasis
gldm LowGrayLevelEmphasis
gldm SmallDependenceEmphasis
gldm SmallDependenceHighGrayLevelEmphasis
glrlm GrayLevelNonUniformity
glrlm LongRunEmphasis
glrlm LongRunHighGrayLevelEmphasis
glrlm LongRunLowGrayLevelEmphasis
glrlm LowGrayLevelRunEmphasis
glrlm RunEntropy
glrlm RunLengthNonUniformityNormalized
glrlm RunPercentage
glrlm RunVariance
glrlm ShortRunEmphasis
glrlm ShortRunLowGrayLevelEmphasis
glszm GrayLevelNonUniformity
glszm GrayLevelNonUniformityNormalized
glszm GrayLevelVariance
glszm HighGrayLevelZoneEmphasis
glszm SizeZoneNonUniformity
glszm SizeZoneNonUniformityNormalized
glszm SmallAreaEmphasis
glszm SmallAreaHighGrayLevelEmphasis
glszm ZoneEntropy
glszm ZonePercentage
ngtdm Busyness
ngtdm Coarseness
ngtdm Complexity
ngtdm Contrast
ngtdm Strength

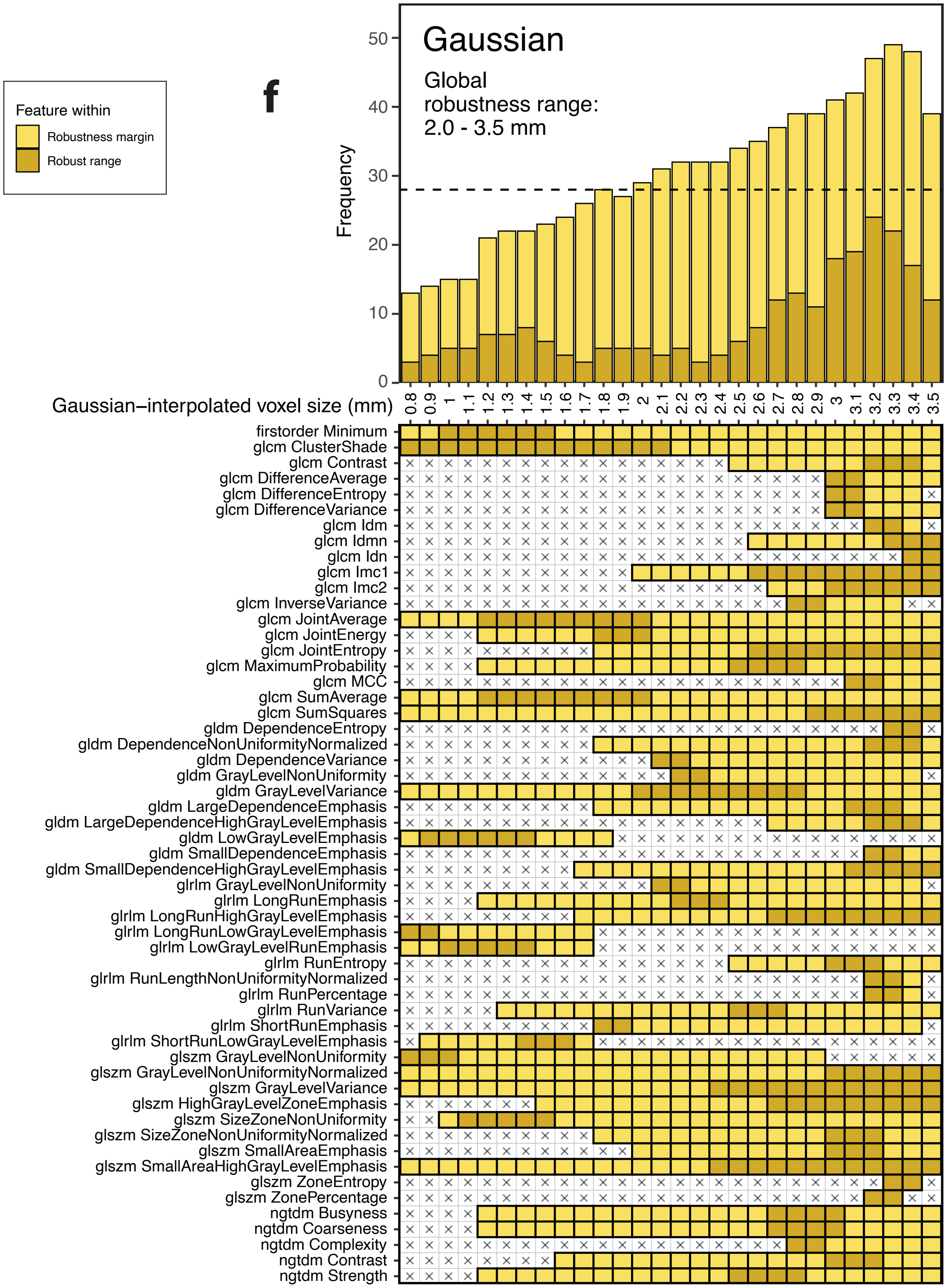

Feature within
Robustness margin
Robust range
f
Gaussian
Global
robustness range:
2.0 - 3.5 mm
Frequency
0
10
20
30
40
50
Gaussian–interpolated voxel size (mm)
0.8 0.9 1 1.1 1.2 1.3 1.4 1.5 1.6 1.7 1.8 1.9 2 2.1 2.2 2.3 2.4 2.5 2.6 2.7 2.8 2.9 3 3.1 3.2 3.3 3.4 3.5
firstorder Minimum
glcm ClusterShade
glcm Contrast
glcm DifferenceAverage
glcm DifferenceEntropy
glcm DifferenceVariance
glcm Idm
glcm Idmn
glcm Idn
glcm Imc1
glcm Imc2
glcm InverseVariance
glcm JointAverage
glcm JointEnergy
glcm JointEntropy
glcm MaximumProbability
glcm MCC
glcm SumAverage
glcm SumSquares
gldm DependenceEntropy
gldm DependenceNonUniformityNormalized
gldm DependenceVariance
gldm GrayLevelNonUniformity
gldm GrayLevelVariance
gldm LargeDependenceEmphasis
gldm LargeDependenceHighGrayLevelEmphasis
gldm LowGrayLevelEmphasis
gldm SmallDependenceEmphasis
gldm SmallDependenceHighGrayLevelEmphasis
glrlm GrayLevelNonUniformity
glrlm LongRunEmphasis
glrlm LongRunHighGrayLevelEmphasis
glrlm LongRunLowGrayLevelEmphasis
glrlm LowGrayLevelRunEmphasis
glrlm RunEntropy
glrlm RunLengthNonUniformityNormalized
glrlm RunPercentage
glrlm RunVariance
glrlm ShortRunEmphasis
glrlm ShortRunLowGrayLevelEmphasis
glszm GrayLevelNonUniformity
glszm GrayLevelNonUniformityNormalized
glszm GrayLevelVariance
glszm HighGrayLevelZoneEmphasis
glszm SizeZoneNonUniformity
glszm SizeZoneNonUniformityNormalized
glszm SmallAreaEmphasis
glszm SmallAreaHighGrayLevelEmphasis
glszm ZoneEntropy
glszm ZonePercentage
ngtdm Busyness
ngtdm Coarseness
ngtdm Complexity
ngtdm Contrast
ngtdm Strength

Supplementary Figure SF3

Global and feature-specific robustness ranges of conditionally robust features in melanoma, for each discretisation (a-b) or interpolation (c-f) algorithm.

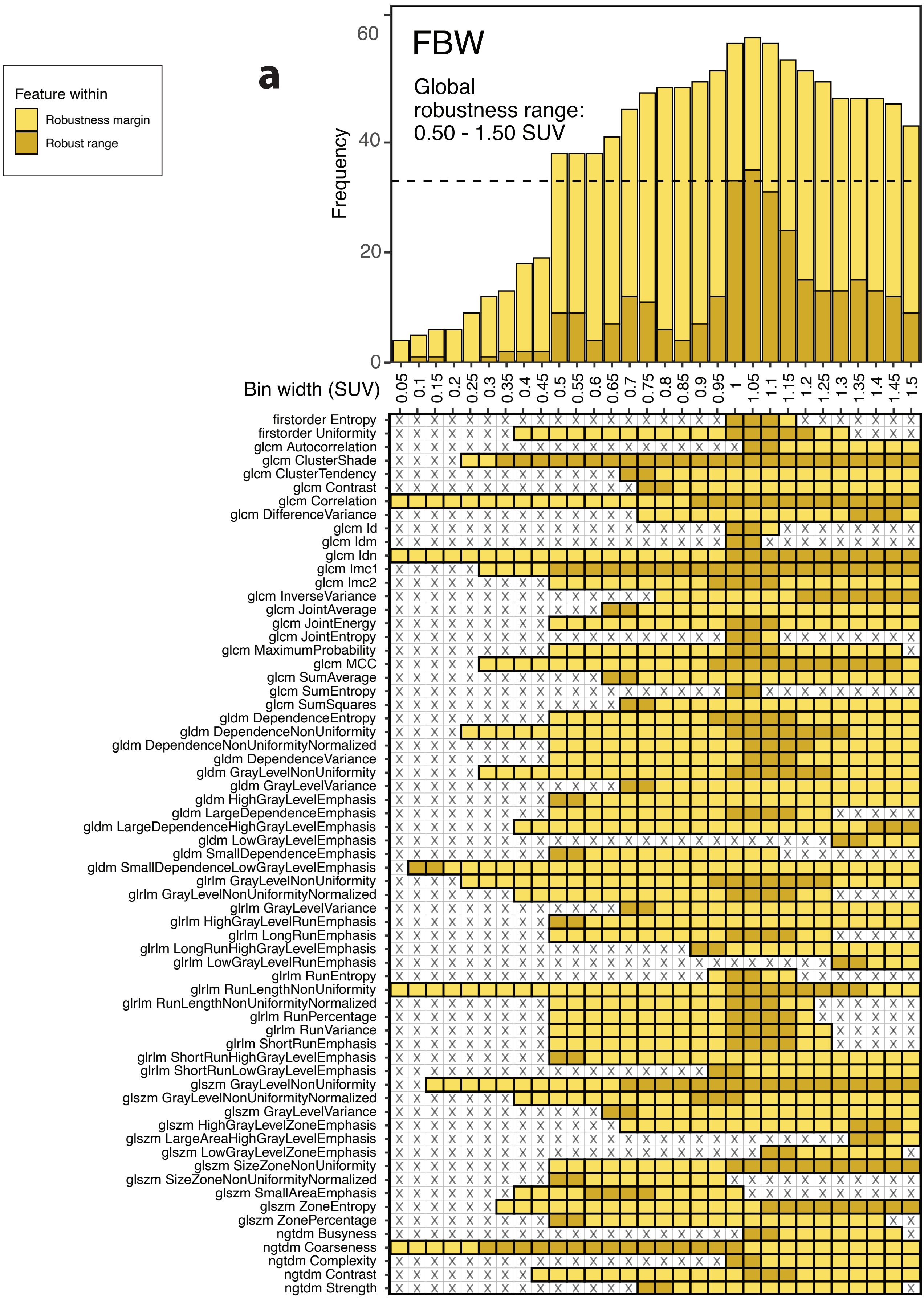

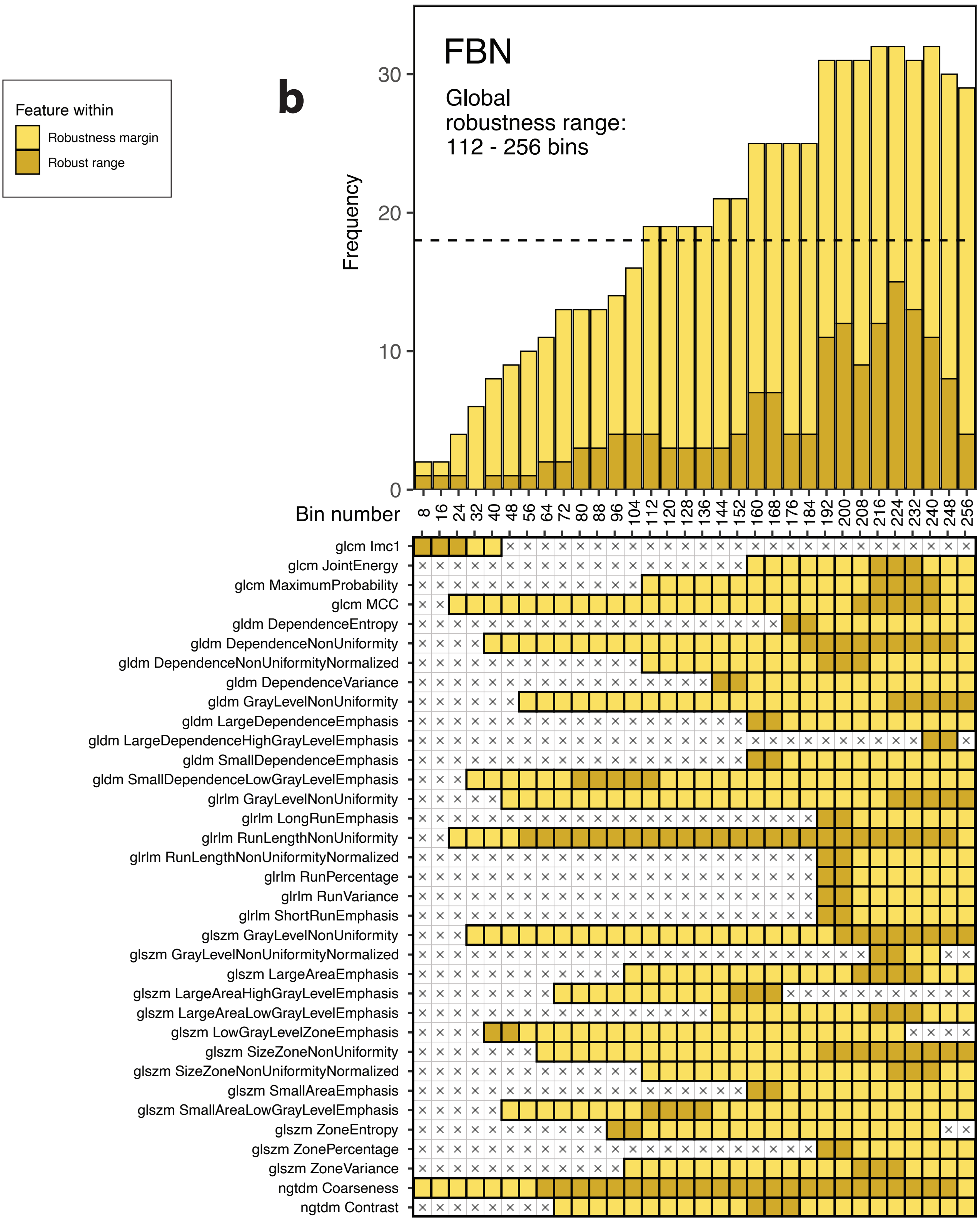
Feature within
Robustness margin
Robust range
b
FBN
Global
robustness range:
112 - 256 bins
Frequency
Bin number
glcm Imc1
glcm JointEnergy
glcm MaximumProbability
glcm MCC
gldm DependenceEntropy
gldm DependenceNonUniformity
gldm DependenceNonUniformityNormalized
gldm DependenceVariance
gldm GrayLevelNonUniformity
gldm LargeDependenceEmphasis
gldm LargeDependenceHighGrayLevelEmphasis
gldm SmallDependenceEmphasis
gldm SmallDependenceLowGrayLevelEmphasis
glrlm GrayLevelNonUniformity
glrlm LongRunEmphasis
glrlm RunLengthNonUniformity
glrlm RunLengthNonUniformityNormalized
glrlm RunPercentage
glrlm RunVariance
glrlm ShortRunEmphasis
glszm GrayLevelNonUniformity
glszm GrayLevelNonUniformityNormalized
glszm LargeAreaEmphasis
glszm LargeAreaHighGrayLevelEmphasis
glszm LargeAreaLowGrayLevelEmphasis
glszm LowGrayLevelZoneEmphasis
glszm SizeZoneNonUniformity
glszm SizeZoneNonUniformityNormalized
glszm SmallAreaEmphasis
glszm SmallAreaLowGrayLevelEmphasis
glszm ZoneEntropy
glszm ZonePercentage
glszm ZoneVariance
ngtdm Coarseness
ngtdm Contrast

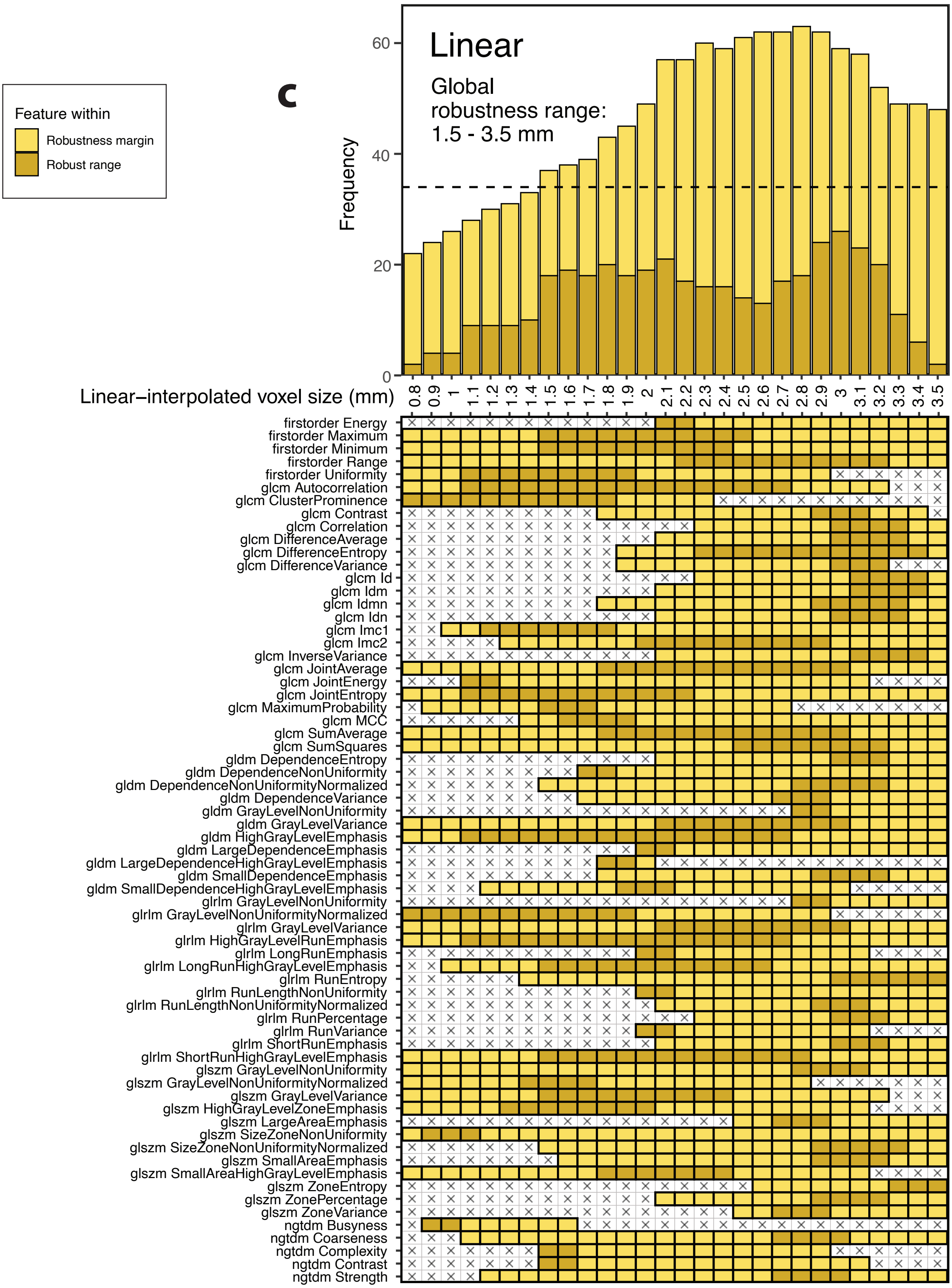
c
Linear
Global
robustness range:
1.5 - 3.5 mm
Feature within
Robustness margin
Robust range
Frequency
0
20
40
60
Linear−interpolated voxel size (mm)
0.8
0.9
1
1.1
1.2
1.3
1.4
1.5
1.6
1.7
1.8
1.9
2
2.1
2.2
2.3
2.4
2.5
2.6
2.7
2.8
2.9
3
3.1
3.2
3.3
3.4
3.5
firstorder Energy
firstorder Maximum
firstorder Minimum
firstorder Range
firstorder Uniformity
glcm Autocorrelation
glcm ClusterProminence
glcm Contrast
glcm Correlation
glcm DifferenceAverage
glcm DifferenceEntropy
glcm DifferenceVariance
glcm Id
glcm Idm
glcm Idmn
glcm Idn
glcm Imc1
glcm Imc2
glcm InverseVariance
glcm JointAverage
glcm JointEnergy
glcm JointEntropy
glcm MaximumProbability
glcm MCC
glcm SumAverage
glcm SumSquares
gldm DependenceEntropy
gldm DependenceNonUniformity
gldm DependenceNonUniformityNormalized
gldm DependenceVariance
gldm GrayLevelNonUniformity
gldm GrayLevelVariance
gldm HighGrayLevelEmphasis
gldm LargeDependenceEmphasis
gldm LargeDependenceHighGrayLevelEmphasis
gldm SmallDependenceEmphasis
gldm SmallDependenceHighGrayLevelEmphasis
glrlm GrayLevelNonUniformity
glrlm GrayLevelNonUniformityNormalized
glrlm GrayLevelVariance
glrlm HighGrayLevelRunEmphasis
glrlm LongRunEmphasis
glrlm LongRunHighGrayLevelEmphasis
glrlm RunEntropy
glrlm RunLengthNonUniformity
glrlm RunLengthNonUniformityNormalized
glrlm RunPercentage
glrlm RunVariance
glrlm ShortRunEmphasis
glrlm ShortRunHighGrayLevelEmphasis
glszm GrayLevelNonUniformity
glszm GrayLevelNonUniformityNormalized
glszm GrayLevelVariance
glszm HighGrayLevelZoneEmphasis
glszm LargeAreaEmphasis
glszm SizeZoneNonUniformity
glszm SizeZoneNonUniformityNormalized
glszm SmallAreaEmphasis
glszm SmallAreaHighGrayLevelEmphasis
glszm ZoneEntropy
glszm ZonePercentage
glszm ZoneVariance
ngtdm Busyness
ngtdm Coarseness
ngtdm Complexity
ngtdm Contrast
ngtdm Strength

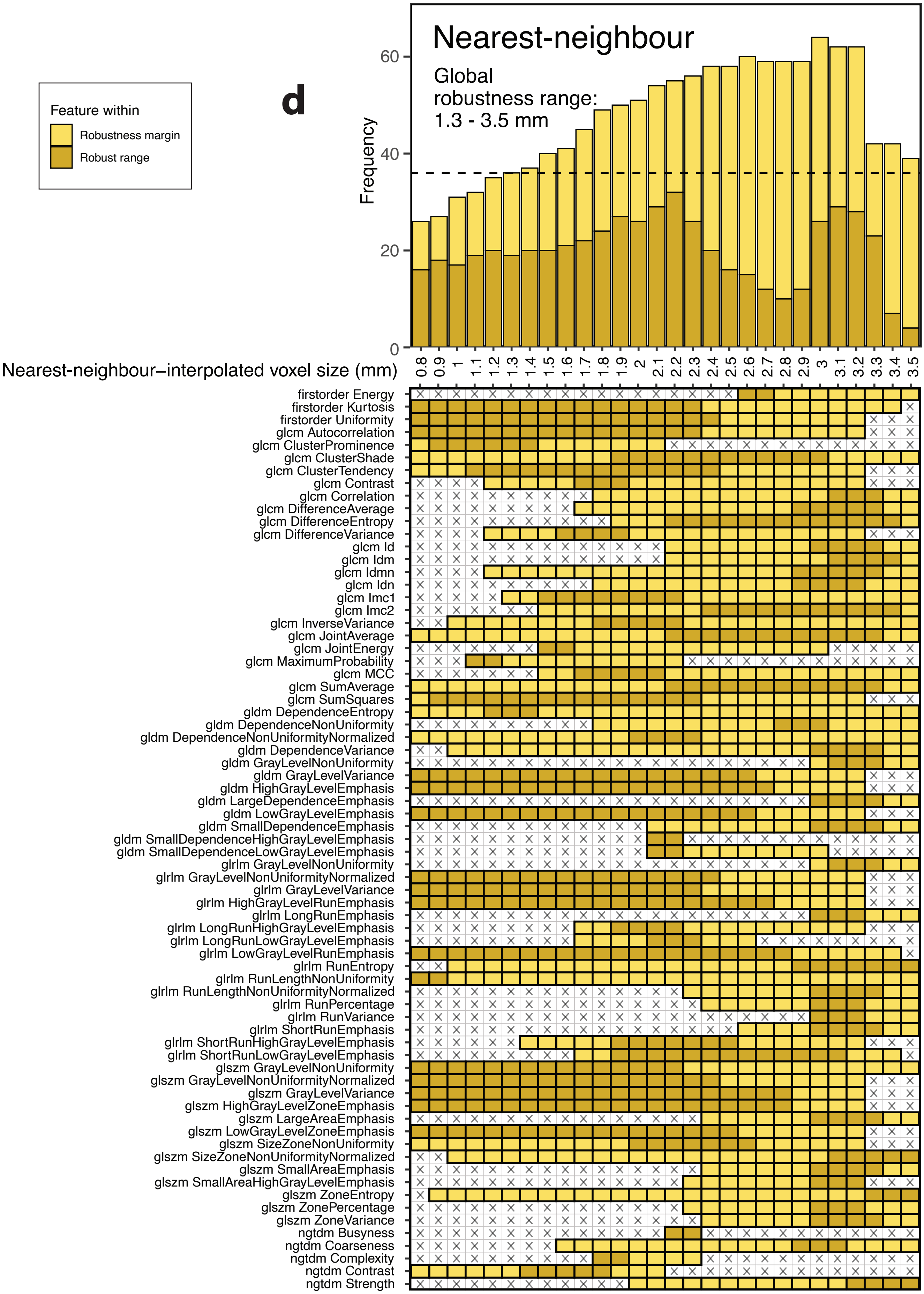
d
Feature within
Robustness margin
Robust range
Nearest-neighbour
Global
robustness range:
1.3 - 3.5 mm
Frequency
0
20
40
60
Nearest-neighbour–interpolated voxel size (mm)
0.8
0.9
1
1.1
1.2
1.3
1.4
1.5
1.6
1.7
1.8
1.9
2
2.1
2.2
2.3
2.4
2.5
2.6
2.7
2.8
2.9
3
3.1
3.2
3.3
3.4
3.5
firstorder Energy
firstorder Kurtosis
firstorder Uniformity
glcm Autocorrelation
glcm ClusterProminence
glcm ClusterShade
glcm ClusterTendency
glcm Contrast
glcm Correlation
glcm DifferenceAverage
glcm DifferenceEntropy
glcm DifferenceVariance
glcm Id
glcm Idm
glcm Idmn
glcm Idn
glcm Imc1
glcm Imc2
glcm InverseVariance
glcm JointAverage
glcm JointEnergy
glcm MaximumProbability
glcm MCC
glcm SumAverage
glcm SumSquares
gldm DependenceEntropy
gldm DependenceNonUniformity
gldm DependenceNonUniformityNormalized
gldm DependenceVariance
gldm GrayLevelNonUniformity
gldm GrayLevelVariance
gldm HighGrayLevelEmphasis
gldm LargeDependenceEmphasis
gldm LowGrayLevelEmphasis
gldm SmallDependenceEmphasis
gldm SmallDependenceHighGrayLevelEmphasis
gldm SmallDependenceLowGrayLevelEmphasis
glrlm GrayLevelNonUniformity
glrlm GrayLevelNonUniformityNormalized
glrlm GrayLevelVariance
glrlm HighGrayLevelRunEmphasis
glrlm LongRunEmphasis
glrlm LongRunHighGrayLevelEmphasis
glrlm LongRunLowGrayLevelEmphasis
glrlm LowGrayLevelRunEmphasis
glrlm RunEntropy
glrlm RunLengthNonUniformity
glrlm RunLengthNonUniformityNormalized
glrlm RunPercentage
glrlm RunVariance
glrlm ShortRunEmphasis
glrlm ShortRunHighGrayLevelEmphasis
glrlm ShortRunLowGrayLevelEmphasis
glszm GrayLevelNonUniformity
glszm GrayLevelNonUniformityNormalized
glszm GrayLevelVariance
glszm HighGrayLevelZoneEmphasis
glszm LargeAreaEmphasis
glszm LowGrayLevelZoneEmphasis
glszm SizeZoneNonUniformity
glszm SizeZoneNonUniformityNormalized
glszm SmallAreaEmphasis
glszm SmallAreaHighGrayLevelEmphasis
glszm ZoneEntropy
glszm ZonePercentage
glszm ZoneVariance
ngtdm Busyness
ngtdm Coarseness
ngtdm Complexity
ngtdm Contrast
ngtdm Strength

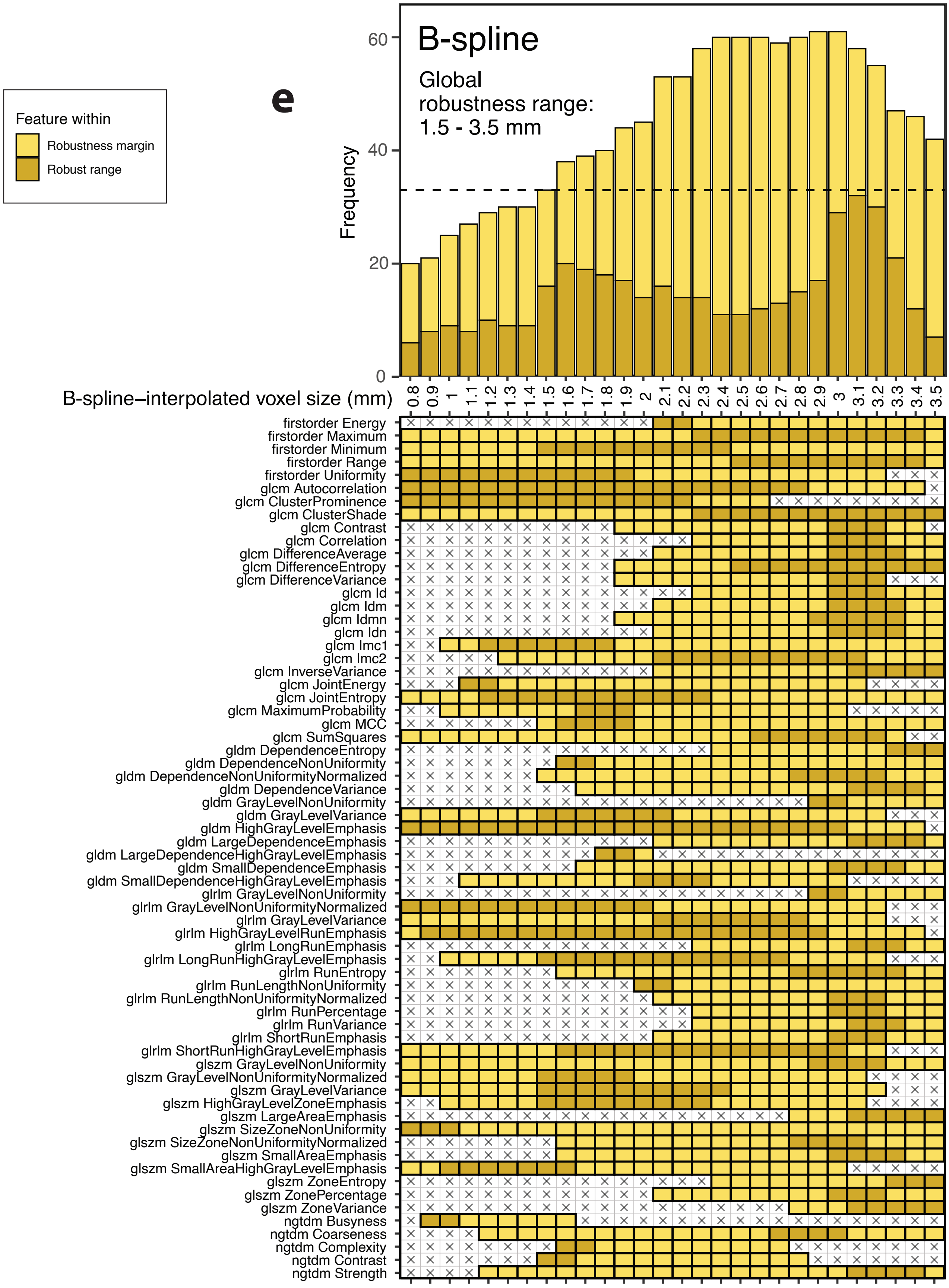
e
Feature within
Robustness margin
Robust range
B-spline
Global robustness range: 1.5 - 3.5 mm
Frequency
0
20
40
60
B-spline−interpolated voxel size (mm)
0.8
0.9
1
1.1
1.2
1.3
1.4
1.5
1.6
1.7
1.8
1.9
2
2.1
2.2
2.3
2.4
2.5
2.6
2.7
2.8
2.9
3
3.1
3.2
3.3
3.4
3.5
firstorder Energy
firstorder Maximum
firstorder Minimum
firstorder Range
firstorder Uniformity
glcm Autocorrelation
glcm ClusterProminence
glcm ClusterShade
glcm Contrast
glcm Correlation
glcm DifferenceAverage
glcm DifferenceEntropy
glcm DifferenceVariance
glcm Id
glcm Idm
glcm Idmn
glcm Idn
glcm Imc1
glcm Imc2
glcm InverseVariance
glcm JointEnergy
glcm JointEntropy
glcm MaximumProbability
glcm MCC
glcm SumSquares
gldm DependenceEntropy
gldm DependenceNonUniformity
gldm DependenceNonUniformityNormalized
gldm DependenceVariance
gldm GrayLevelNonUniformity
gldm GrayLevelVariance
gldm HighGrayLevelEmphasis
gldm LargeDependenceEmphasis
gldm LargeDependenceHighGrayLevelEmphasis
gldm SmallDependenceEmphasis
gldm SmallDependenceHighGrayLevelEmphasis
glrlm GrayLevelNonUniformity
glrlm GrayLevelNonUniformityNormalized
glrlm GrayLevelVariance
glrlm HighGrayLevelRunEmphasis
glrlm LongRunEmphasis
glrlm LongRunHighGrayLevelEmphasis
glrlm RunEntropy
glrlm RunLengthNonUniformity
glrlm RunLengthNonUniformityNormalized
glrlm RunPercentage
glrlm RunVariance
glrlm ShortRunEmphasis
glrlm ShortRunHighGrayLevelEmphasis
glszm GrayLevelNonUniformity
glszm GrayLevelNonUniformityNormalized
glszm GrayLevelVariance
glszm HighGrayLevelZoneEmphasis
glszm LargeAreaEmphasis
glszm SizeZoneNonUniformity
glszm SizeZoneNonUniformityNormalized
glszm SmallAreaEmphasis
glszm SmallAreaHighGrayLevelEmphasis
glszm ZoneEntropy
glszm ZonePercentage
glszm ZoneVariance
ngtdm Busyness
ngtdm Coarseness
ngtdm Complexity
ngtdm Contrast
ngtdm Strength

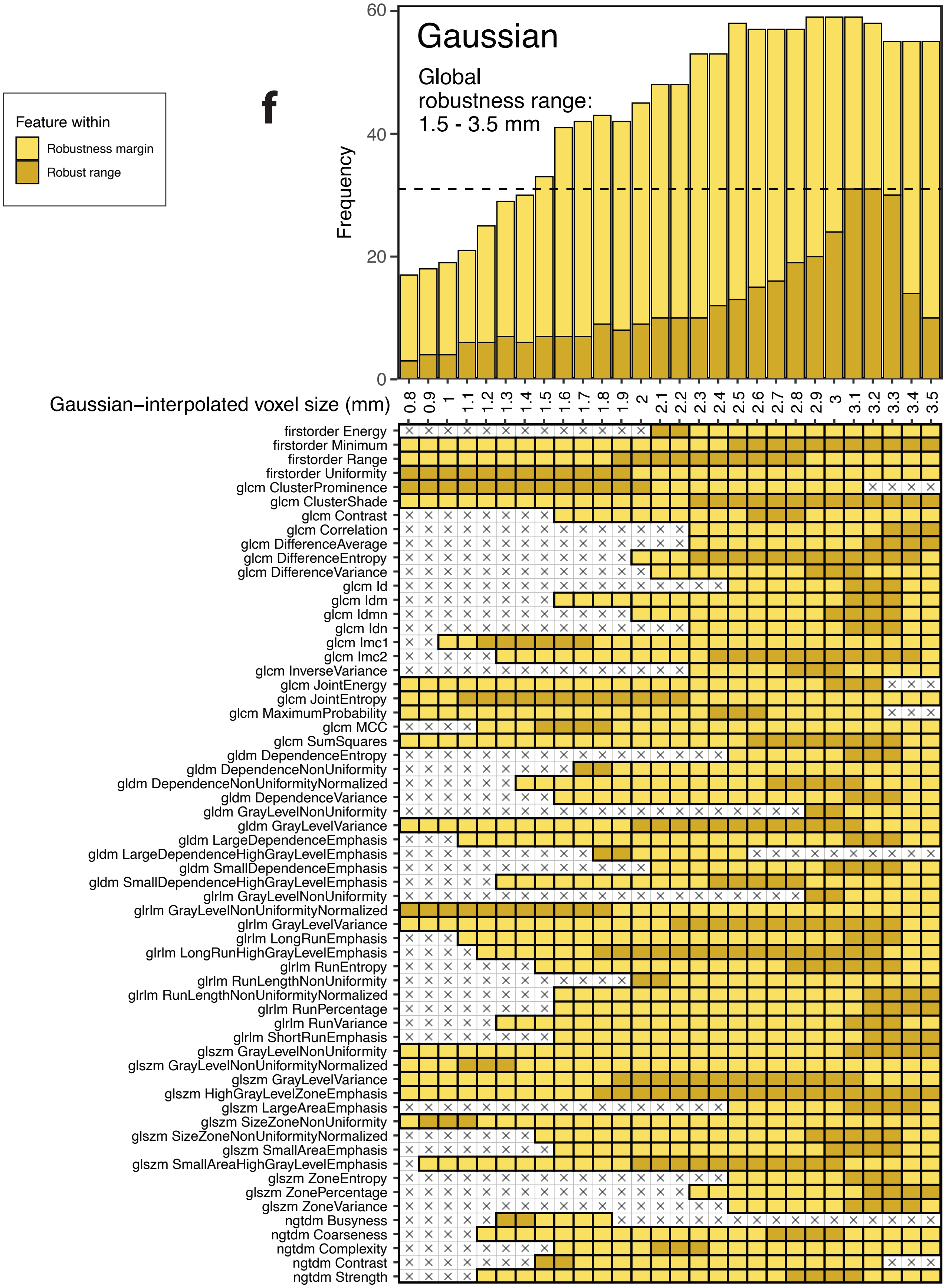
Feature within
Robustness margin
Robust range
f
Gaussian
Global
robustness range:
1.5 - 3.5 mm
Frequency
60
40
20
0
Gaussian−interpolated voxel size (mm)
0.8
0.9
1
1.1
1.2
1.3
1.4
1.5
1.6
1.7
1.8
1.9
2
2.1
2.2
2.3
2.4
2.5
2.6
2.7
2.8
2.9
3
3.1
3.2
3.3
3.4
3.5
firstorder Energy
firstorder Minimum
firstorder Range
firstorder Uniformity
glcm ClusterProminence
glcm ClusterShade
glcm Contrast
glcm Correlation
glcm DifferenceAverage
glcm DifferenceEntropy
glcm DifferenceVariance
glcm Id
glcm Idm
glcm Idmn
glcm Idn
glcm Imc1
glcm Imc2
glcm InverseVariance
glcm JointEnergy
glcm JointEntropy
glcm MaximumProbability
glcm MCC
glcm SumSquares
gldm DependenceEntropy
gldm DependenceNonUniformity
gldm DependenceNonUniformityNormalized
gldm DependenceVariance
gldm GrayLevelNonUniformity
gldm GrayLevelVariance
gldm LargeDependenceEmphasis
gldm LargeDependenceHighGrayLevelEmphasis
gldm SmallDependenceEmphasis
gldm SmallDependenceHighGrayLevelEmphasis
glrlm GrayLevelNonUniformity
glrlm GrayLevelNonUniformityNormalized
glrlm GrayLevelVariance
glrlm LongRunEmphasis
glrlm LongRunHighGrayLevelEmphasis
glrlm RunEntropy
glrlm RunLengthNonUniformity
glrlm RunLengthNonUniformityNormalized
glrlm RunPercentage
glrlm RunVariance
glrlm ShortRunEmphasis
glszm GrayLevelNonUniformity
glszm GrayLevelNonUniformityNormalized
glszm GrayLevelVariance
glszm HighGrayLevelZoneEmphasis
glszm LargeAreaEmphasis
glszm SizeZoneNonUniformity
glszm SizeZoneNonUniformityNormalized
glszm SmallAreaEmphasis
glszm SmallAreaHighGrayLevelEmphasis
glszm ZoneEntropy
glszm ZonePercentage
glszm ZoneVariance
ngtdm Busyness
ngtdm Coarseness
ngtdm Complexity
ngtdm Contrast
ngtdm Strength

Supplementary Figure SF4

Global and feature-specific robustness ranges of conditionally robust features in lymphoma, for each discretisation (a-b) or interpolation (c-f) algorithm.

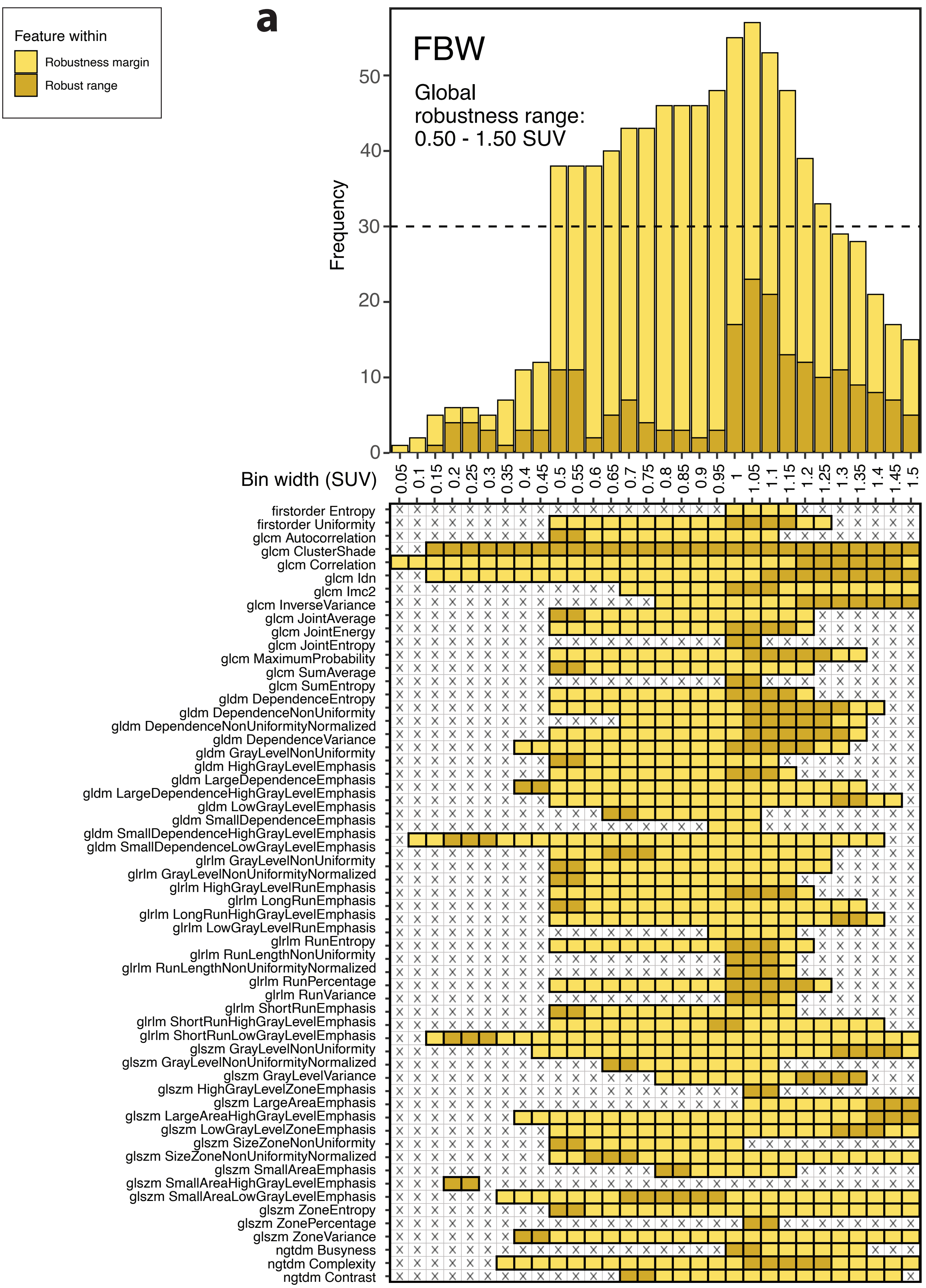

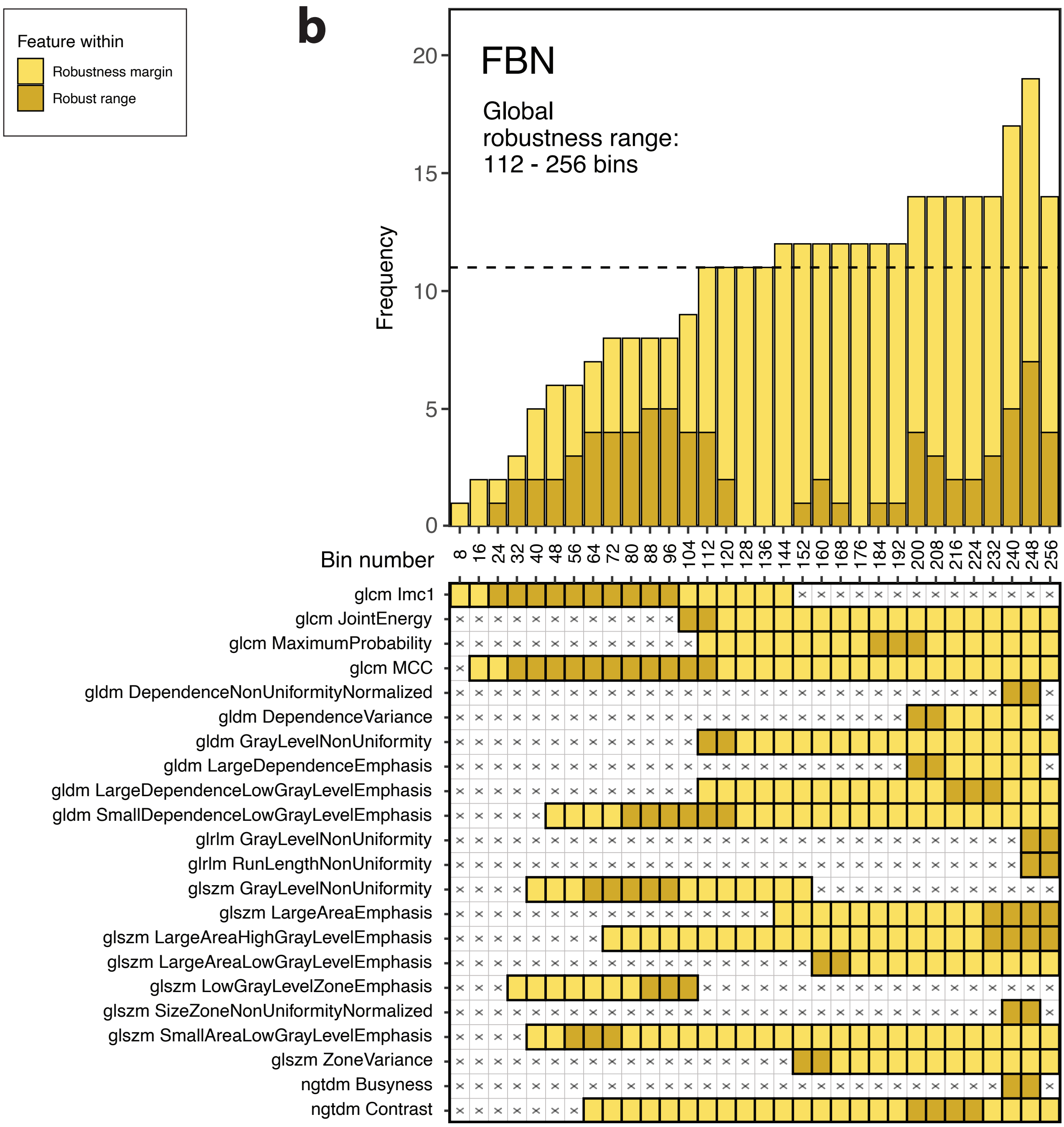

Feature within
Robustness margin
Robust range
b
FBN
Global
robustness range:
112 - 256 bins
Frequency
Bin number
glcm Imc1
glcm JointEnergy
glcm MaximumProbability
glcm MCC
gldm DependenceNonUniformityNormalized
gldm DependenceVariance
gldm GrayLevelNonUniformity
gldm LargeDependenceEmphasis
gldm LargeDependenceLowGrayLevelEmphasis
gldm SmallDependenceLowGrayLevelEmphasis
glrlm GrayLevelNonUniformity
glrlm RunLengthNonUniformity
glszm GrayLevelNonUniformity
glszm LargeAreaEmphasis
glszm LargeAreaHighGrayLevelEmphasis
glszm LargeAreaLowGrayLevelEmphasis
glszm LowGrayLevelZoneEmphasis
glszm SizeZoneNonUniformityNormalized
glszm SmallAreaLowGrayLevelEmphasis
glszm ZoneVariance
ngtdm Busyness
ngtdm Contrast

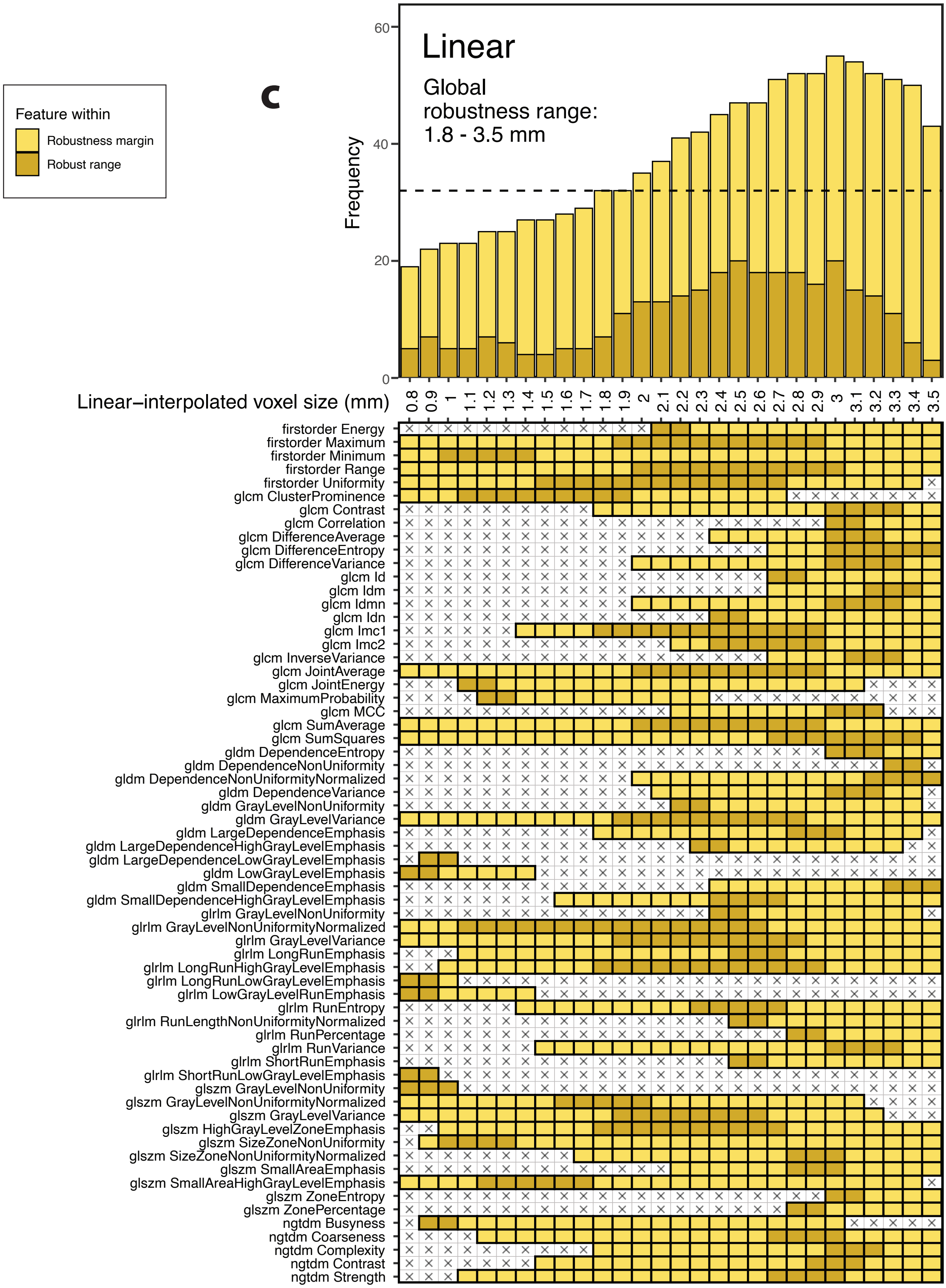

Feature within
Robustness margin
Robust range
c
Linear
Global
robustness range:
1.8 - 3.5 mm
Frequency
0
20
40
60
Linear–interpolated voxel size (mm)
0.8 0.9 1 1.1 1.2 1.3 1.4 1.5 1.6 1.7 1.8 1.9 2 2.1 2.2 2.3 2.4 2.5 2.6 2.7 2.8 2.9 3 3.1 3.2 3.3 3.4 3.5
firstorder Energy
firstorder Maximum
firstorder Minimum
firstorder Range
firstorder Uniformity
glcm ClusterProminence
glcm Contrast
glcm Correlation
glcm DifferenceAverage
glcm DifferenceEntropy
glcm DifferenceVariance
glcm Id
glcm Idm
glcm Idmn
glcm Idn
glcm Imc1
glcm Imc2
glcm InverseVariance
glcm JointAverage
glcm JointEnergy
glcm MaximumProbability
glcm MCC
glcm SumAverage
glcm SumSquares
gldm DependenceEntropy
gldm DependenceNonUniformity
gldm DependenceNonUniformityNormalized
gldm DependenceVariance
gldm GrayLevelNonUniformity
gldm GrayLevelVariance
gldm LargeDependenceEmphasis
gldm LargeDependenceHighGrayLevelEmphasis
gldm LargeDependenceLowGrayLevelEmphasis
gldm LowGrayLevelEmphasis
gldm SmallDependenceEmphasis
gldm SmallDependenceHighGrayLevelEmphasis
glrlm GrayLevelNonUniformity
glrlm GrayLevelNonUniformityNormalized
glrlm GrayLevelVariance
glrlm LongRunEmphasis
glrlm LongRunHighGrayLevelEmphasis
glrlm LongRunLowGrayLevelEmphasis
glrlm LowGrayLevelRunEmphasis
glrlm RunEntropy
glrlm RunLengthNonUniformityNormalized
glrlm RunPercentage
glrlm RunVariance
glrlm ShortRunEmphasis
glrlm ShortRunLowGrayLevelEmphasis
glszm GrayLevelNonUniformity
glszm GrayLevelNonUniformityNormalized
glszm GrayLevelVariance
glszm HighGrayLevelZoneEmphasis
glszm SizeZoneNonUniformity
glszm SizeZoneNonUniformityNormalized
glszm SmallAreaEmphasis
glszm SmallAreaHighGrayLevelEmphasis
glszm ZoneEntropy
glszm ZonePercentage
ngtdm Busyness
ngtdm Coarseness
ngtdm Complexity
ngtdm Contrast
ngtdm Strength

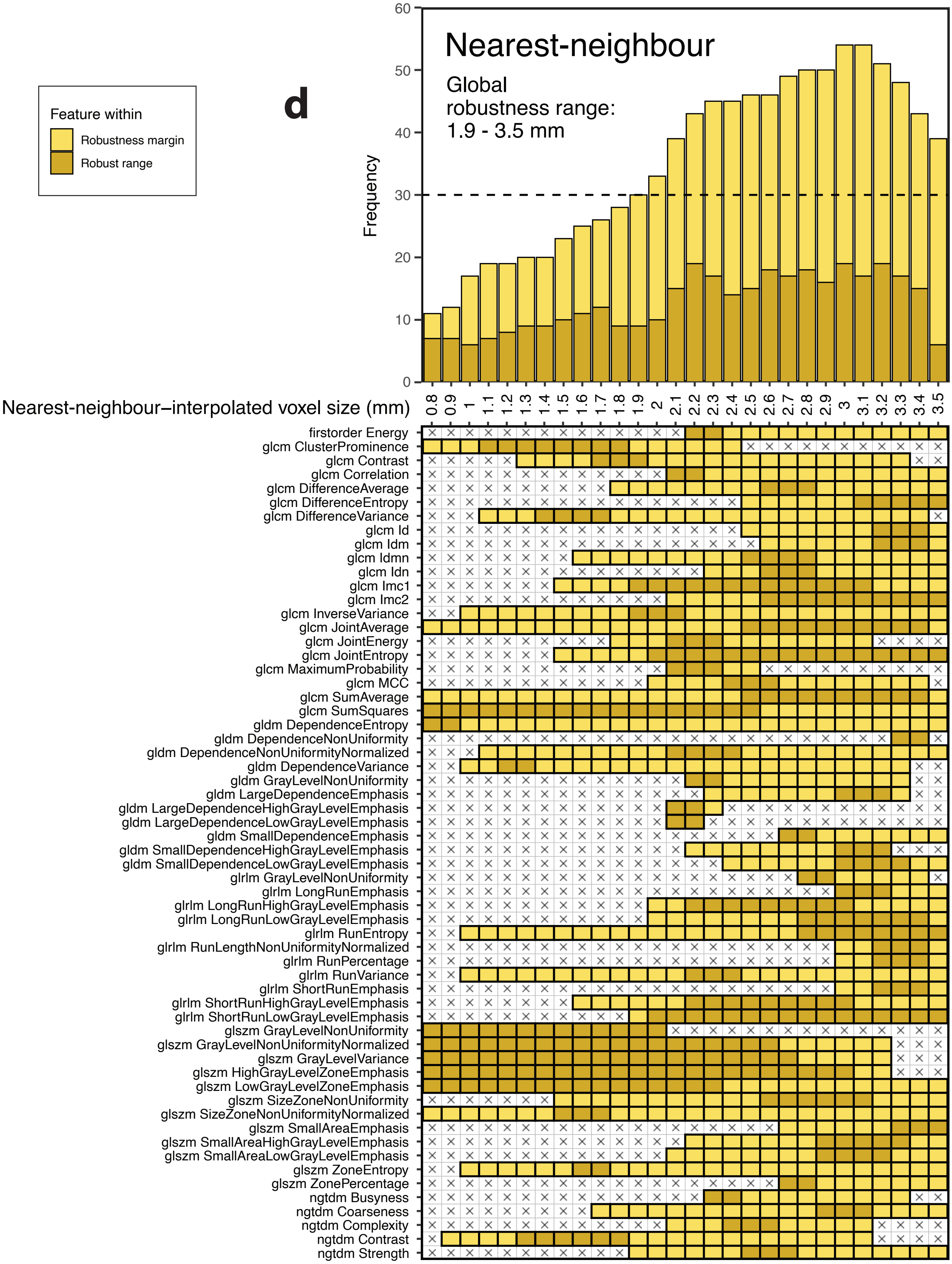
d
Feature within
Robustness margin
Robust range
Nearest-neighbour
Global
robustness range:
1.9 - 3.5 mm
Frequency
0
10
20
30
40
50
60
Nearest-neighbour–interpolated voxel size (mm)
0.8
0.9
1
1.1
1.2
1.3
1.4
1.5
1.6
1.7
1.8
1.9
2
2.1
2.2
2.3
2.4
2.5
2.6
2.7
2.8
2.9
3
3.1
3.2
3.3
3.4
3.5
firstorder Energy
glcm ClusterProminence
glcm Contrast
glcm Correlation
glcm DifferenceAverage
glcm DifferenceEntropy
glcm DifferenceVariance
glcm Id
glcm Idm
glcm Idmn
glcm Idn
glcm Imc1
glcm Imc2
glcm InverseVariance
glcm JointAverage
glcm JointEnergy
glcm JointEntropy
glcm MaximumProbability
glcm MCC
glcm SumAverage
glcm SumSquares
gldm DependenceEntropy
gldm DependenceNonUniformity
gldm DependenceNonUniformityNormalized
gldm DependenceVariance
gldm GrayLevelNonUniformity
gldm LargeDependenceEmphasis
gldm LargeDependenceHighGrayLevelEmphasis
gldm LargeDependenceLowGrayLevelEmphasis
gldm SmallDependenceEmphasis
gldm SmallDependenceHighGrayLevelEmphasis
gldm SmallDependenceLowGrayLevelEmphasis
glrlm GrayLevelNonUniformity
glrlm LongRunEmphasis
glrlm LongRunHighGrayLevelEmphasis
glrlm LongRunLowGrayLevelEmphasis
glrlm RunEntropy
glrlm RunLengthNonUniformityNormalized
glrlm RunPercentage
glrlm RunVariance
glrlm ShortRunEmphasis
glrlm ShortRunHighGrayLevelEmphasis
glrlm ShortRunLowGrayLevelEmphasis
glszm GrayLevelNonUniformity
glszm GrayLevelNonUniformityNormalized
glszm GrayLevelVariance
glszm HighGrayLevelZoneEmphasis
glszm LowGrayLevelZoneEmphasis
glszm SizeZoneNonUniformity
glszm SizeZoneNonUniformityNormalized
glszm SmallAreaEmphasis
glszm SmallAreaHighGrayLevelEmphasis
glszm SmallAreaLowGrayLevelEmphasis
glszm ZoneEntropy
glszm ZonePercentage
ngtdm Busyness
ngtdm Coarseness
ngtdm Complexity
ngtdm Contrast
ngtdm Strength

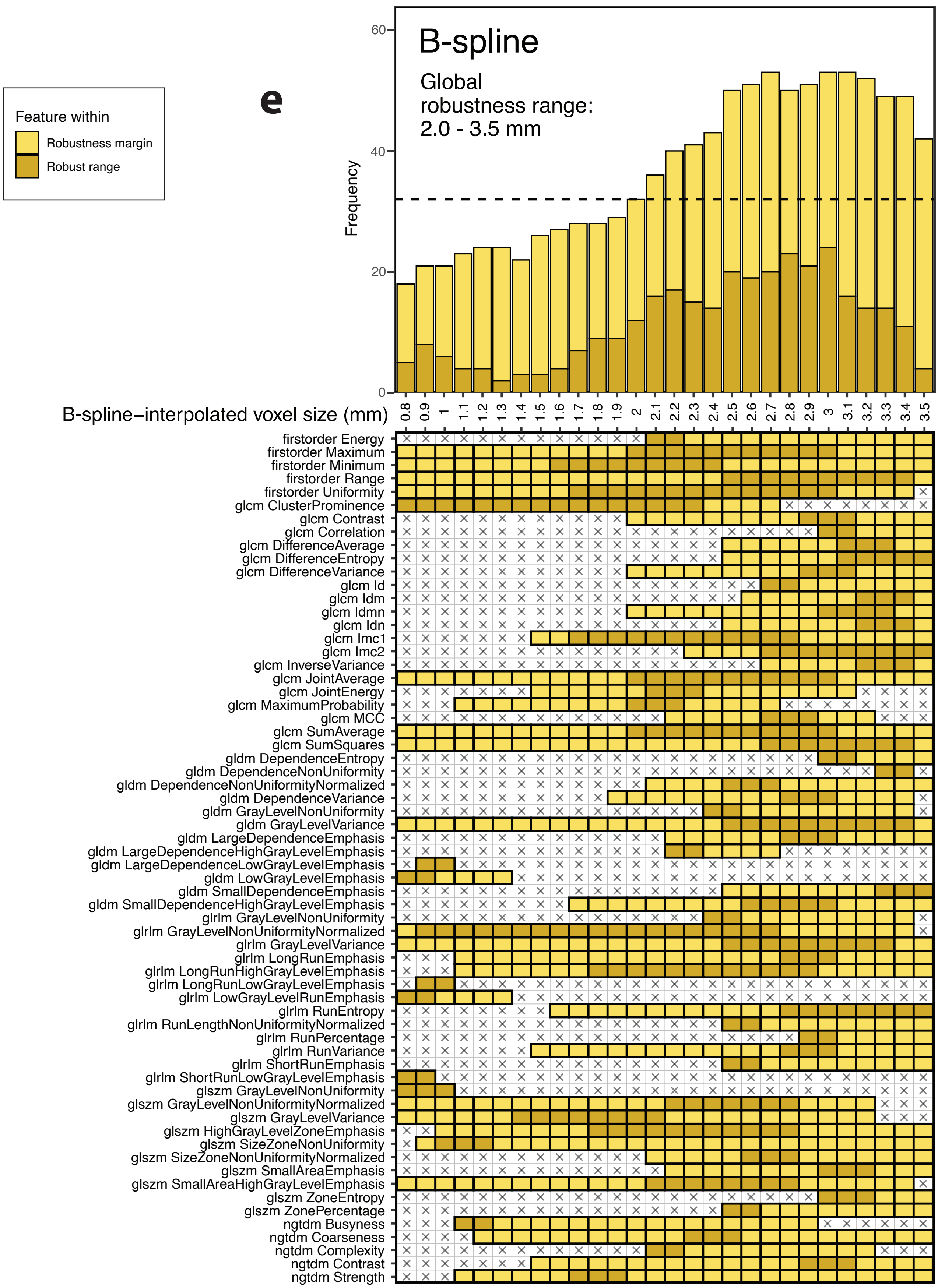
e
Feature within
Robustness margin
Robust range
B-spline
Global robustness range: 2.0 - 3.5 mm
Frequency
B-spline–interpolated voxel size (mm)
firstorder Energy
firstorder Maximum
firstorder Minimum
firstorder Range
firstorder Uniformity
glcm ClusterProminence
glcm Contrast
glcm Correlation
glcm DifferenceAverage
glcm DifferenceEntropy
glcm DifferenceVariance
glcm Id
glcm Idm
glcm Idmn
glcm Idn
glcm Imc1
glcm Imc2
glcm InverseVariance
glcm JointAverage
glcm JointEnergy
glcm MaximumProbability
glcm MCC
glcm SumAverage
glcm SumSquares
gldm DependenceEntropy
gldm DependenceNonUniformity
gldm DependenceNonUniformityNormalized
gldm DependenceVariance
gldm GrayLevelNonUniformity
gldm GrayLevelVariance
gldm LargeDependenceEmphasis
gldm LargeDependenceHighGrayLevelEmphasis
gldm LargeDependenceLowGrayLevelEmphasis
gldm LowGrayLevelEmphasis
gldm SmallDependenceEmphasis
gldm SmallDependenceHighGrayLevelEmphasis
glrlm GrayLevelNonUniformity
glrlm GrayLevelNonUniformityNormalized
glrlm GrayLevelVariance
glrlm LongRunEmphasis
glrlm LongRunHighGrayLevelEmphasis
glrlm LongRunLowGrayLevelEmphasis
glrlm LowGrayLevelRunEmphasis
glrlm RunEntropy
glrlm RunLengthNonUniformityNormalized
glrlm RunPercentage
glrlm RunVariance
glrlm ShortRunEmphasis
glrlm ShortRunLowGrayLevelEmphasis
glszm GrayLevelNonUniformity
glszm GrayLevelNonUniformityNormalized
glszm GrayLevelVariance
glszm HighGrayLevelZoneEmphasis
glszm SizeZoneNonUniformity
glszm SizeZoneNonUniformityNormalized
glszm SmallAreaEmphasis
glszm SmallAreaHighGrayLevelEmphasis
glszm ZoneEntropy
glszm ZonePercentage
ngtdm Busyness
ngtdm Coarseness
ngtdm Complexity
ngtdm Contrast
ngtdm Strength

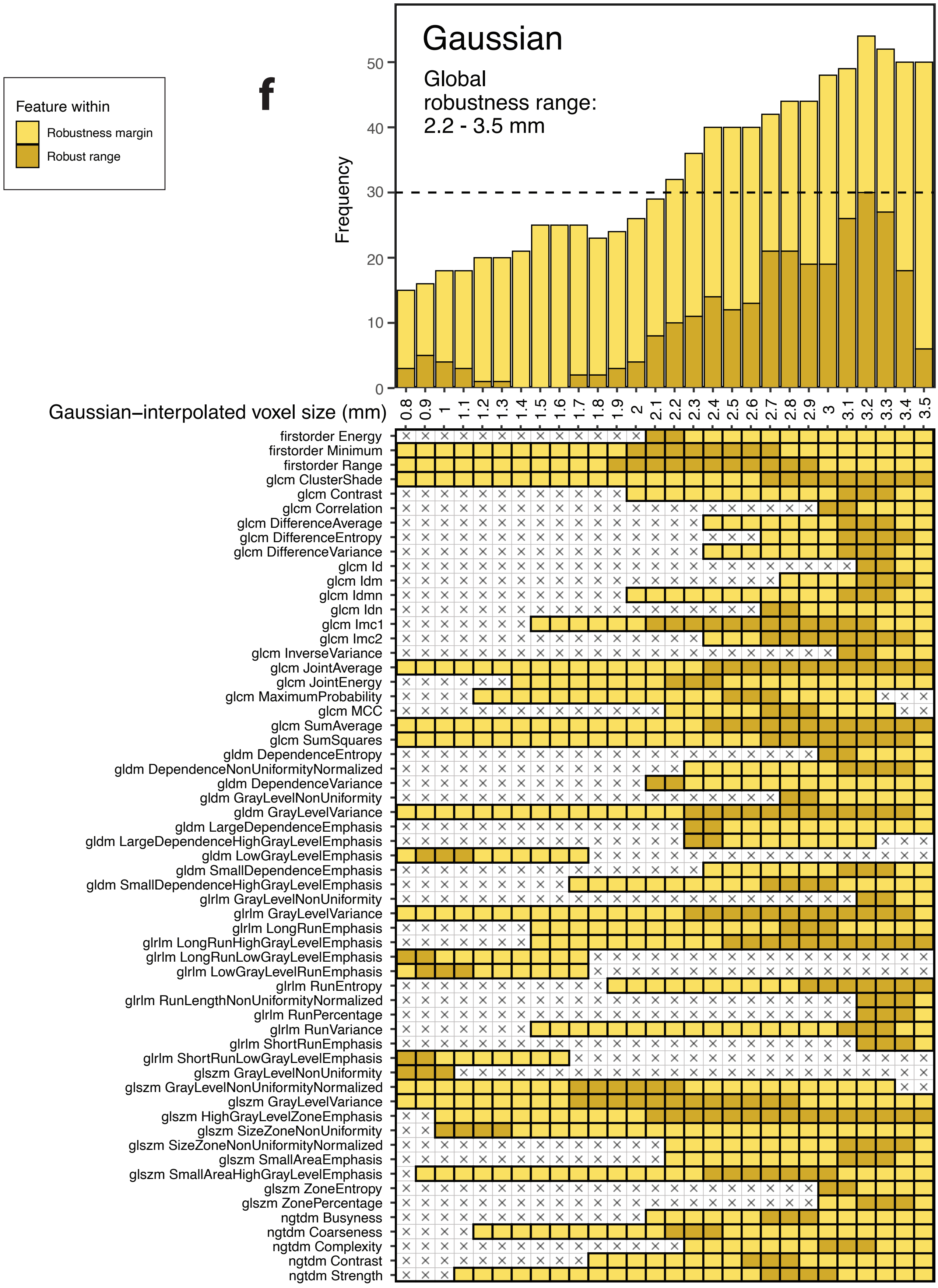
f
Feature within
Robustness margin
Robust range
Gaussian
Global
robustness range:
2.2 - 3.5 mm
Frequency
0
10
20
30
40
50
Gaussian−interpolated voxel size (mm)
0.8 0.9 1 1.1 1.2 1.3 1.4 1.5 1.6 1.7 1.8 1.9 2 2.1 2.2 2.3 2.4 2.5 2.6 2.7 2.8 2.9 3 3.1 3.2 3.3 3.4 3.5
firstorder Energy
firstorder Minimum
firstorder Range
glcm ClusterShade
glcm Contrast
glcm Correlation
glcm DifferenceAverage
glcm DifferenceEntropy
glcm DifferenceVariance
glcm Id
glcm Idm
glcm Idmn
glcm Idn
glcm Imc1
glcm Imc2
glcm InverseVariance
glcm JointAverage
glcm JointEnergy
glcm MaximumProbability
glcm MCC
glcm SumAverage
glcm SumSquares
gldm DependenceEntropy
gldm DependenceNonUniformityNormalized
gldm DependenceVariance
gldm GrayLevelNonUniformity
gldm GrayLevelVariance
gldm LargeDependenceEmphasis
gldm LargeDependenceHighGrayLevelEmphasis
gldm LowGrayLevelEmphasis
gldm SmallDependenceEmphasis
gldm SmallDependenceHighGrayLevelEmphasis
glrlm GrayLevelNonUniformity
glrlm GrayLevelVariance
glrlm LongRunEmphasis
glrlm LongRunHighGrayLevelEmphasis
glrlm LongRunLowGrayLevelEmphasis
glrlm LowGrayLevelRunEmphasis
glrlm RunEntropy
glrlm RunLengthNonUniformityNormalized
glrlm RunPercentage
glrlm RunVariance
glrlm ShortRunEmphasis
glrlm ShortRunLowGrayLevelEmphasis
glszm GrayLevelNonUniformity
glszm GrayLevelNonUniformityNormalized
glszm GrayLevelVariance
glszm HighGrayLevelZoneEmphasis
glszm SizeZoneNonUniformity
glszm SizeZoneNonUniformityNormalized
glszm SmallAreaEmphasis
glszm SmallAreaHighGrayLevelEmphasis
glszm ZoneEntropy
glszm ZonePercentage
ngtdm Busyness
ngtdm Coarseness
ngtdm Complexity
ngtdm Contrast
ngtdm Strength

Supplementary Figure SF5

Correctability categorisations mosaic for radiomic features not meeting the robust criteria, stratified by image processing algorithm and cancer type.

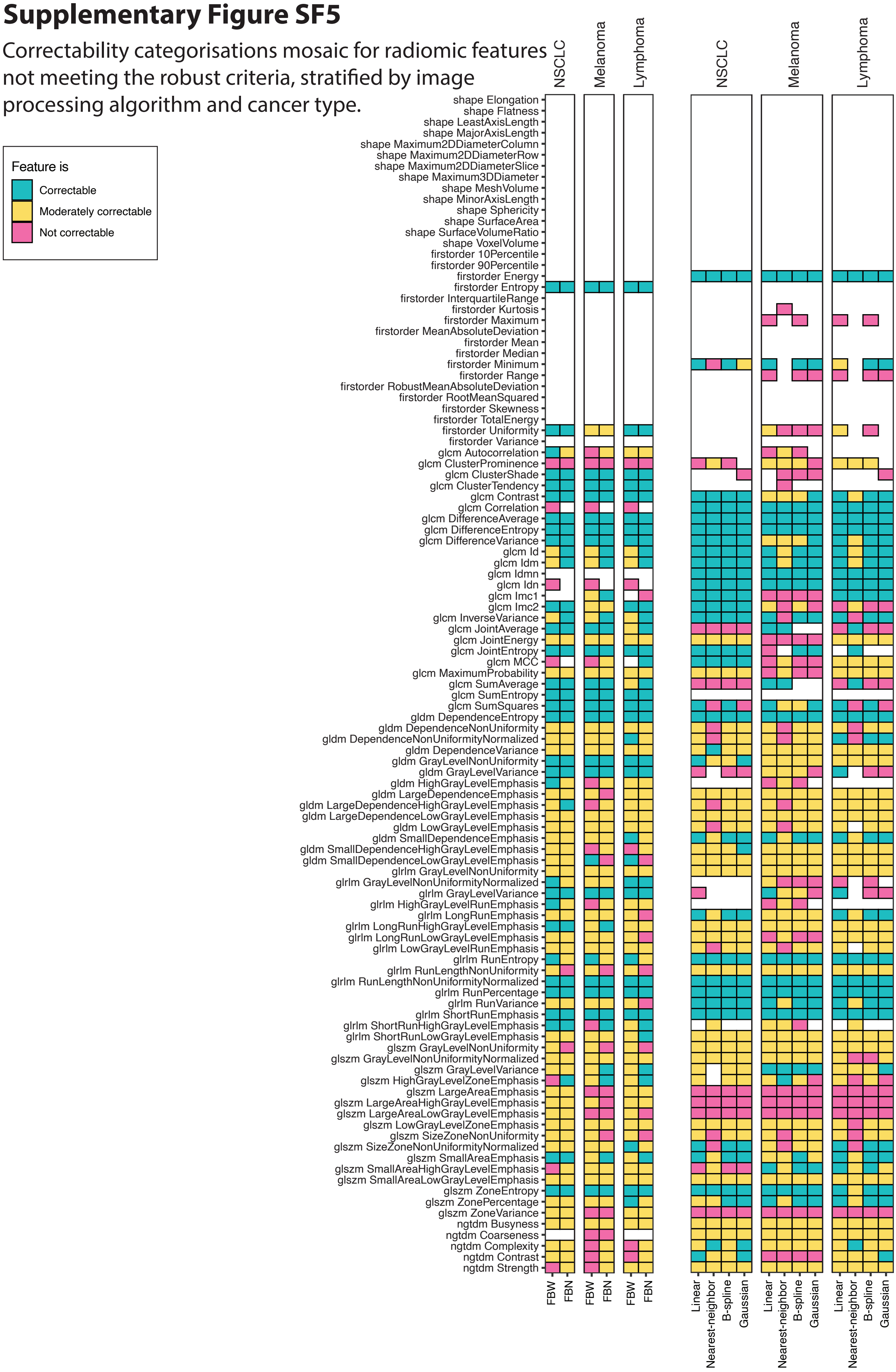

Supplementary Figure SF6

Best-fit equations used for correction of radiomic features not meeting the robust criteria, stratified by image processing algorithm and cancer type.

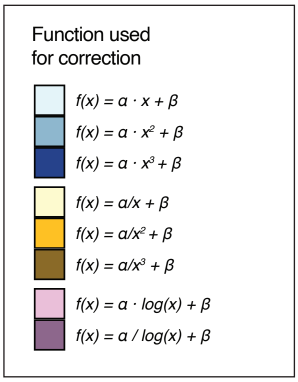

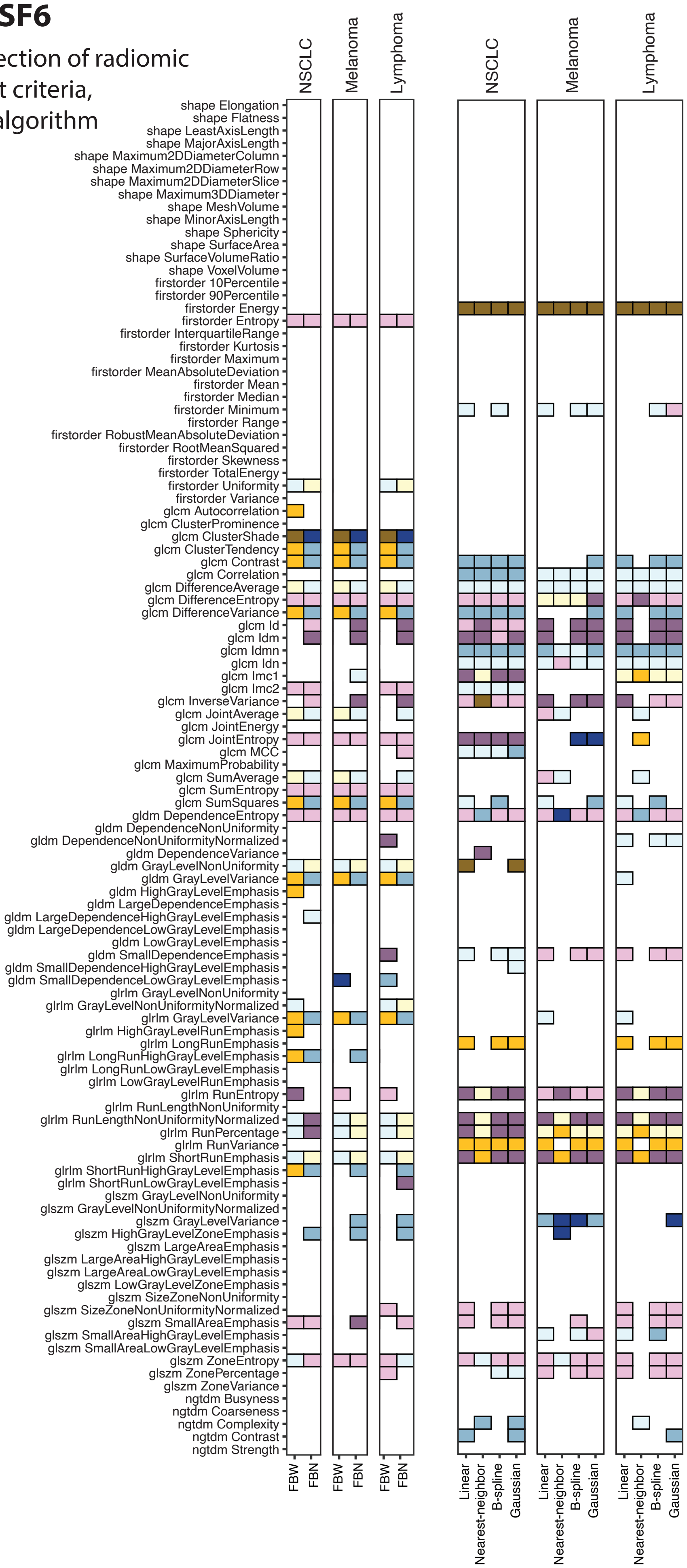

Supplementary Figure SF7

Dumbbell plots showing the change in CV_{mean} and $|r_{rm}|$ for correctable features in NSCLC, when considering the different discretisation (a-b) and interpolation (c-f) algorithms tested. Features are ranked by the change in feature variability and correlation upon correction.

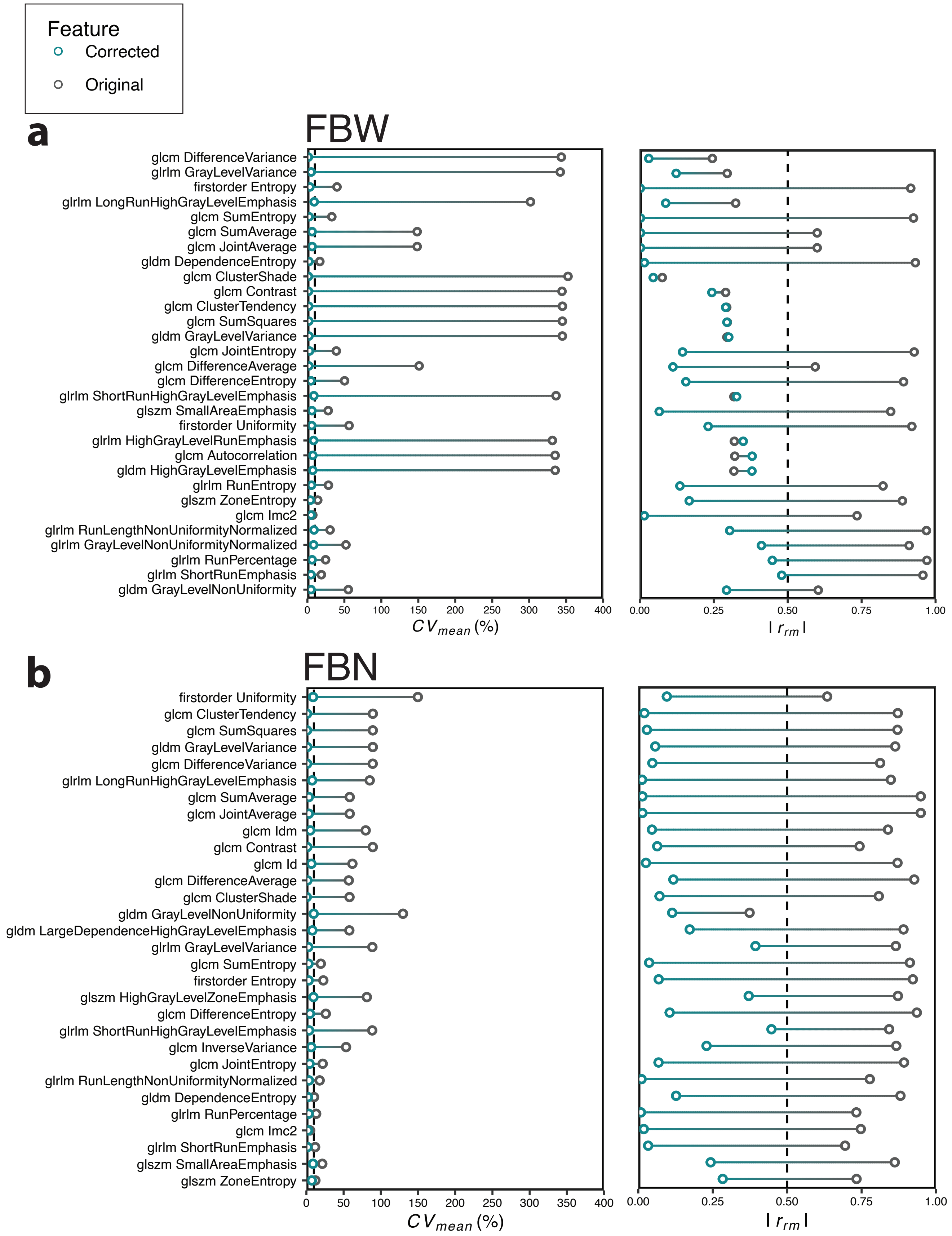

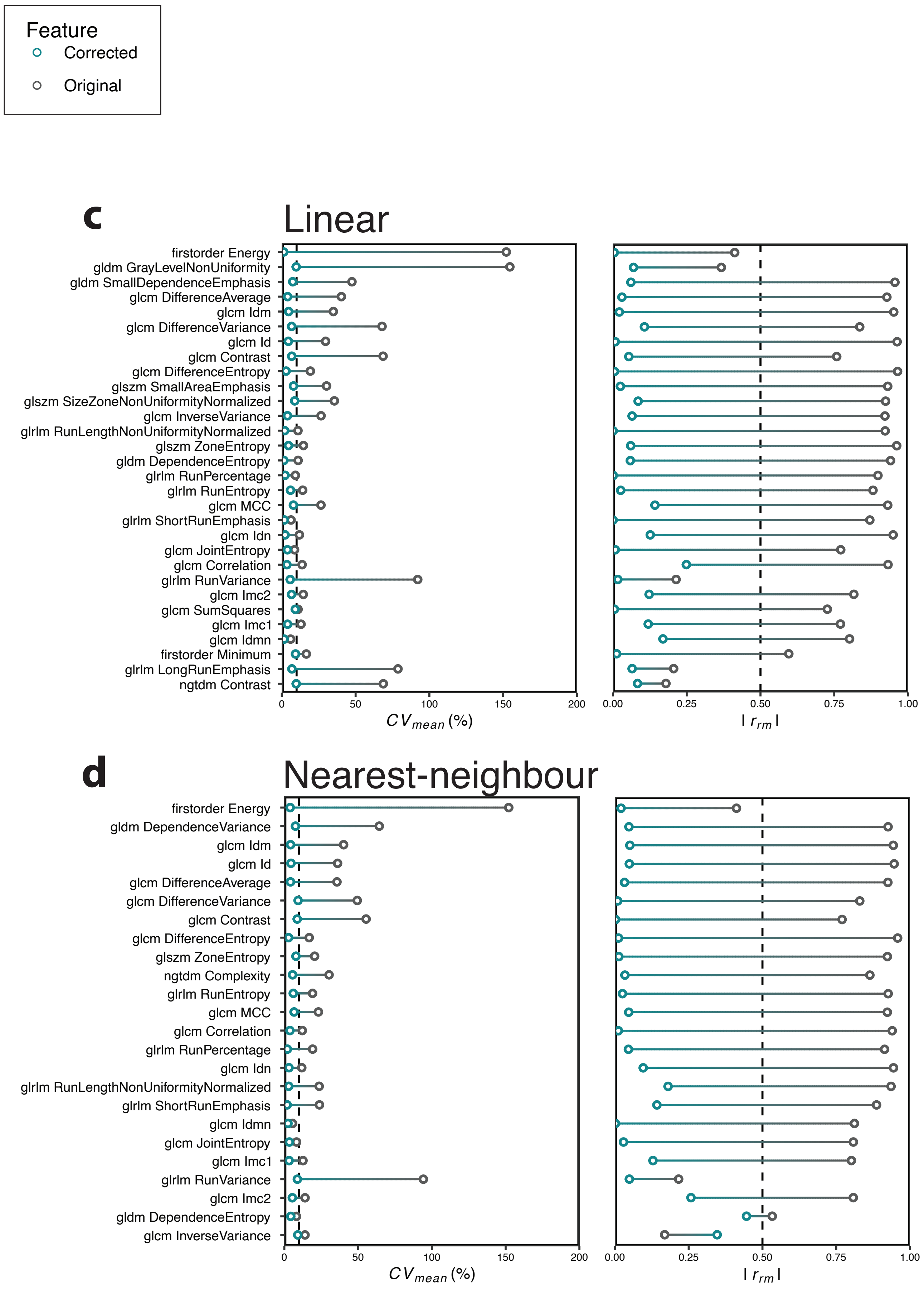
Feature
Corrected
Original
c
Linear
firstorder Energy
gldm GrayLevelNonUniformity
gldm SmallDependenceEmphasis
glcm DifferenceAverage
glcm Idm
glcm DifferenceVariance
glcm Id
glcm Contrast
glcm DifferenceEntropy
glszm SmallAreaEmphasis
glszm SizeZoneNonUniformityNormalized
glcm InverseVariance
glrlm RunLengthNonUniformityNormalized
glszm ZoneEntropy
gldm DependenceEntropy
glrlm RunPercentage
glrlm RunEntropy
glcm MCC
glrlm ShortRunEmphasis
glcm Idn
glcm JointEntropy
glcm Correlation
glrlm RunVariance
glcm Imc2
glcm SumSquares
glcm Imc1
glcm Idmn
firstorder Minimum
glrlm LongRunEmphasis
ngtdm Contrast
0 50 100 150 200
CV_{mean} (%)
0.00 0.25 0.50 0.75 1.00
$|r_{rm}|$
d
Nearest-neighbour
firstorder Energy
gldm DependenceVariance
glcm Idm
glcm Id
glcm DifferenceAverage
glcm DifferenceVariance
glcm Contrast
glcm DifferenceEntropy
glszm ZoneEntropy
ngtdm Complexity
glrlm RunEntropy
glcm MCC
glcm Correlation
glrlm RunPercentage
glcm Idn
glrlm RunLengthNonUniformityNormalized
glrlm ShortRunEmphasis
glcm Idmn
glcm JointEntropy
glcm Imc1
glrlm RunVariance
glcm Imc2
gldm DependenceEntropy
glcm InverseVariance
0 50 100 150 200
CV_{mean} (%)
0.00 0.25 0.50 0.75 1.00
$|r_{rm}|$

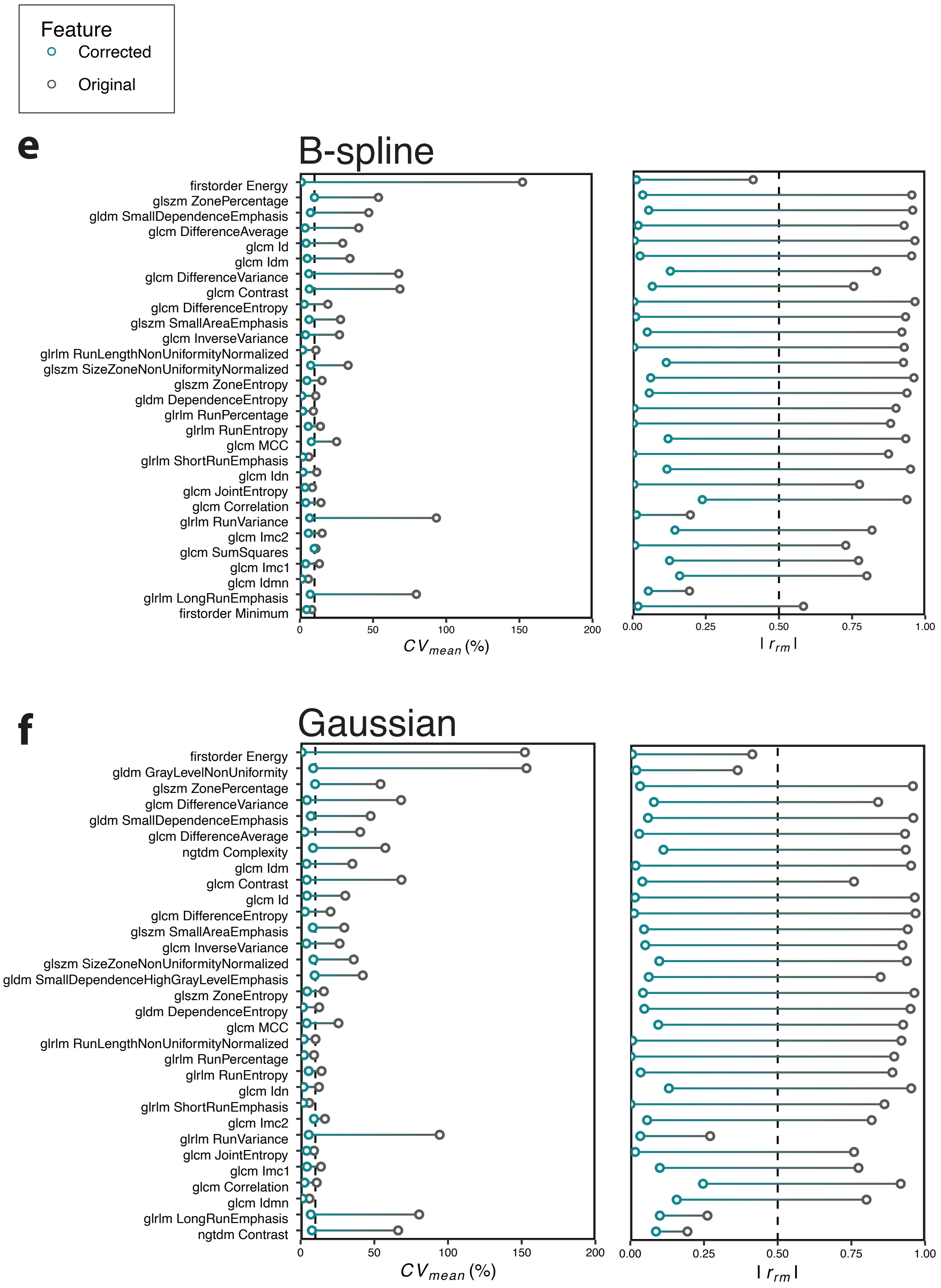

Feature
Corrected
Original
e
B-spline
firstorder Energy
glszm ZonePercentage
gldm SmallDependenceEmphasis
glcm DifferenceAverage
glcm Id
glcm Idm
glcm DifferenceVariance
glcm Contrast
glcm DifferenceEntropy
glszm SmallAreaEmphasis
glcm InverseVariance
glrlm RunLengthNonUniformityNormalized
glszm SizeZoneNonUniformityNormalized
glszm ZoneEntropy
gldm DependenceEntropy
glrlm RunPercentage
glrlm RunEntropy
glcm MCC
glrlm ShortRunEmphasis
glcm Idn
glcm JointEntropy
glcm Correlation
glrlm RunVariance
glcm Imc2
glcm SumSquares
glcm Imc1
glcm Idmn
glrlm LongRunEmphasis
firstorder Minimum
0 50 100 150 200
CV_{mean} (%)
0.00 0.25 0.50 0.75 1.00
$|r_{rm}|$
f
Gaussian
firstorder Energy
gldm GrayLevelNonUniformity
glszm ZonePercentage
glcm DifferenceVariance
gldm SmallDependenceEmphasis
glcm DifferenceAverage
ngtdm Complexity
glcm Idm
glcm Contrast
glcm Id
glcm DifferenceEntropy
glszm SmallAreaEmphasis
glcm InverseVariance
glszm SizeZoneNonUniformityNormalized
gldm SmallDependenceHighGrayLevelEmphasis
glszm ZoneEntropy
gldm DependenceEntropy
glcm MCC
glrlm RunLengthNonUniformityNormalized
glrlm RunPercentage
glrlm RunEntropy
glcm Idn
glrlm ShortRunEmphasis
glcm Imc2
glrlm RunVariance
glcm JointEntropy
glcm Imc1
glcm Correlation
glcm Idmn
glrlm LongRunEmphasis
ngtdm Contrast
0 50 100 150 200
CV_{mean} (%)
0.00 0.25 0.50 0.75 1.00
$|r_{rm}|$

Supplementary Figure SF8

Dumbbell plots showing the change in CV_{mean} and $|r_{rm}|$ for correctable features in melanoma, when considering the different discretisation (a-b) and interpolation (c-f) algorithms tested. Features are ranked by the change in feature variability and correlation upon correction.

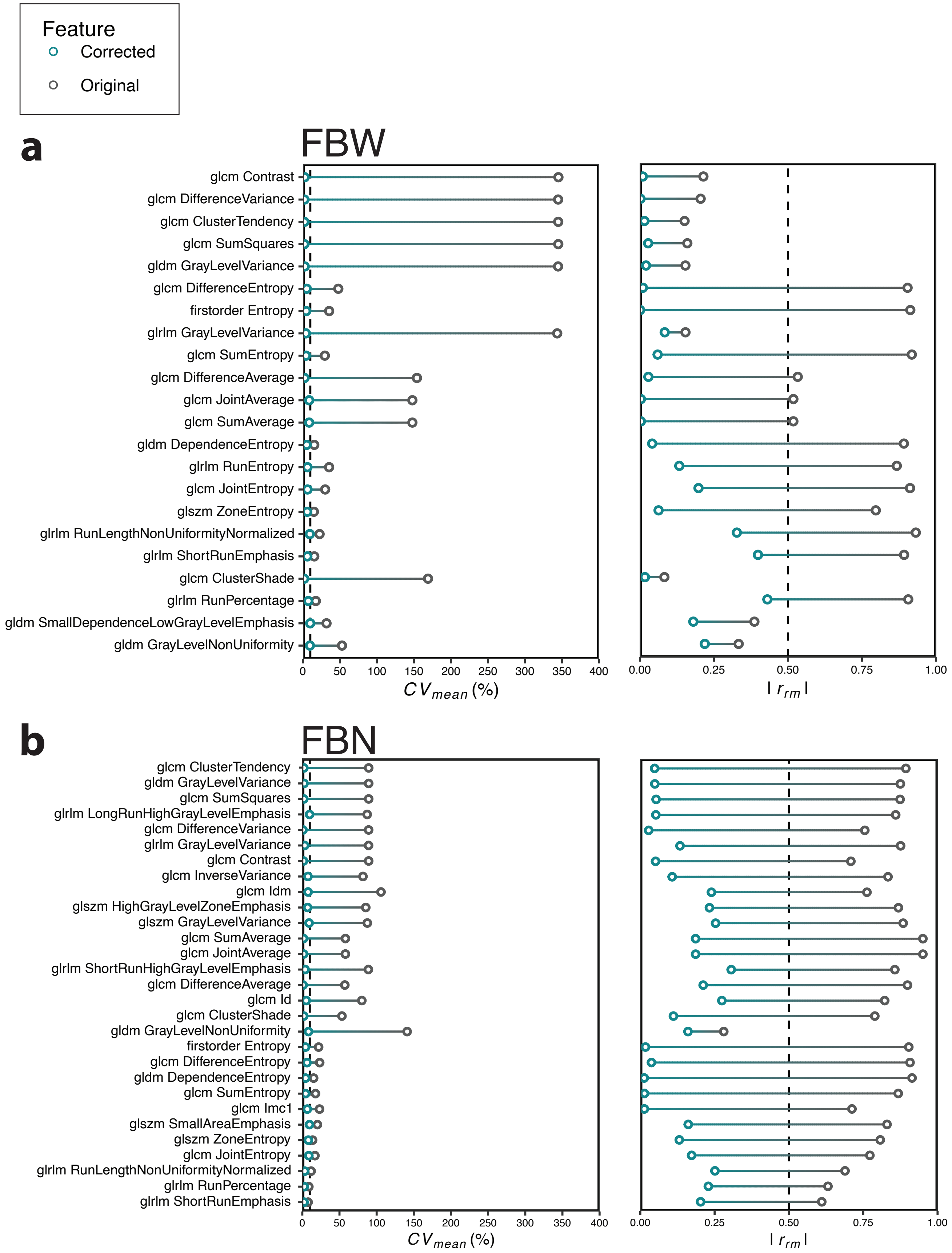

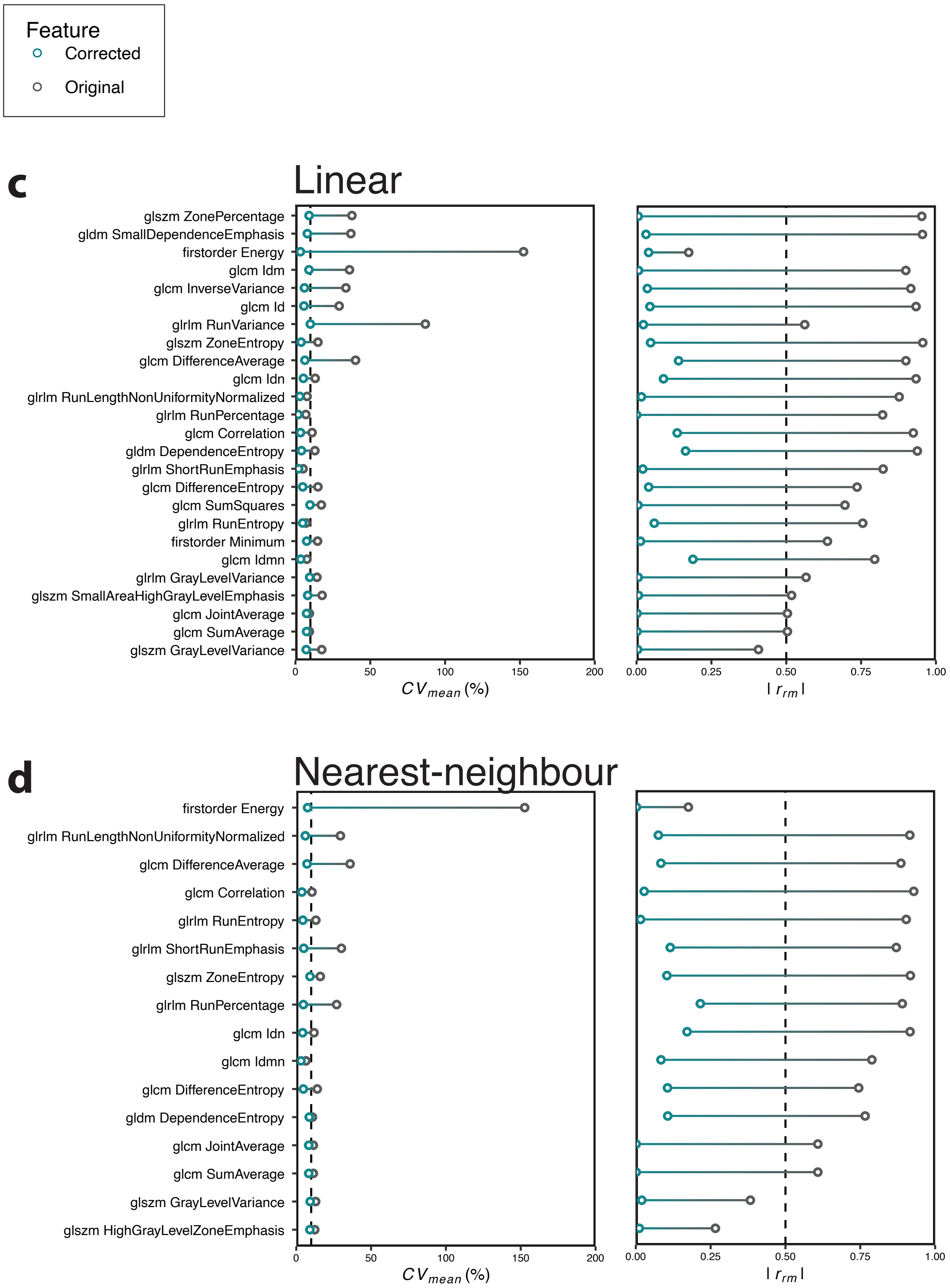
Feature
Corrected
Original
c
Linear
glszm ZonePercentage
gldm SmallDependenceEmphasis
firstorder Energy
glcm Idm
glcm InverseVariance
glcm Id
glrlm RunVariance
glszm ZoneEntropy
glcm DifferenceAverage
glcm Idn
glrlm RunLengthNonUniformityNormalized
glrlm RunPercentage
glcm Correlation
gldm DependenceEntropy
glrlm ShortRunEmphasis
glcm DifferenceEntropy
glcm SumSquares
glrlm RunEntropy
firstorder Minimum
glcm Idmn
glrlm GrayLevelVariance
glszm SmallAreaHighGrayLevelEmphasis
glcm JointAverage
glcm SumAverage
glszm GrayLevelVariance
0
50
100
150
200
CV_{mean} (%)
0.00
0.25
0.50
0.75
1.00
$|r_{rm}|$
d
Nearest-neighbour
firstorder Energy
glrlm RunLengthNonUniformityNormalized
glcm DifferenceAverage
glcm Correlation
glrlm RunEntropy
glrlm ShortRunEmphasis
glszm ZoneEntropy
glrlm RunPercentage
glcm Idn
glcm Idmn
glcm DifferenceEntropy
gldm DependenceEntropy
glcm JointAverage
glcm SumAverage
glszm GrayLevelVariance
glszm HighGrayLevelZoneEmphasis
0
50
100
150
200
CV_{mean} (%)
0.00
0.25
0.50
0.75
1.00
$|r_{rm}|$

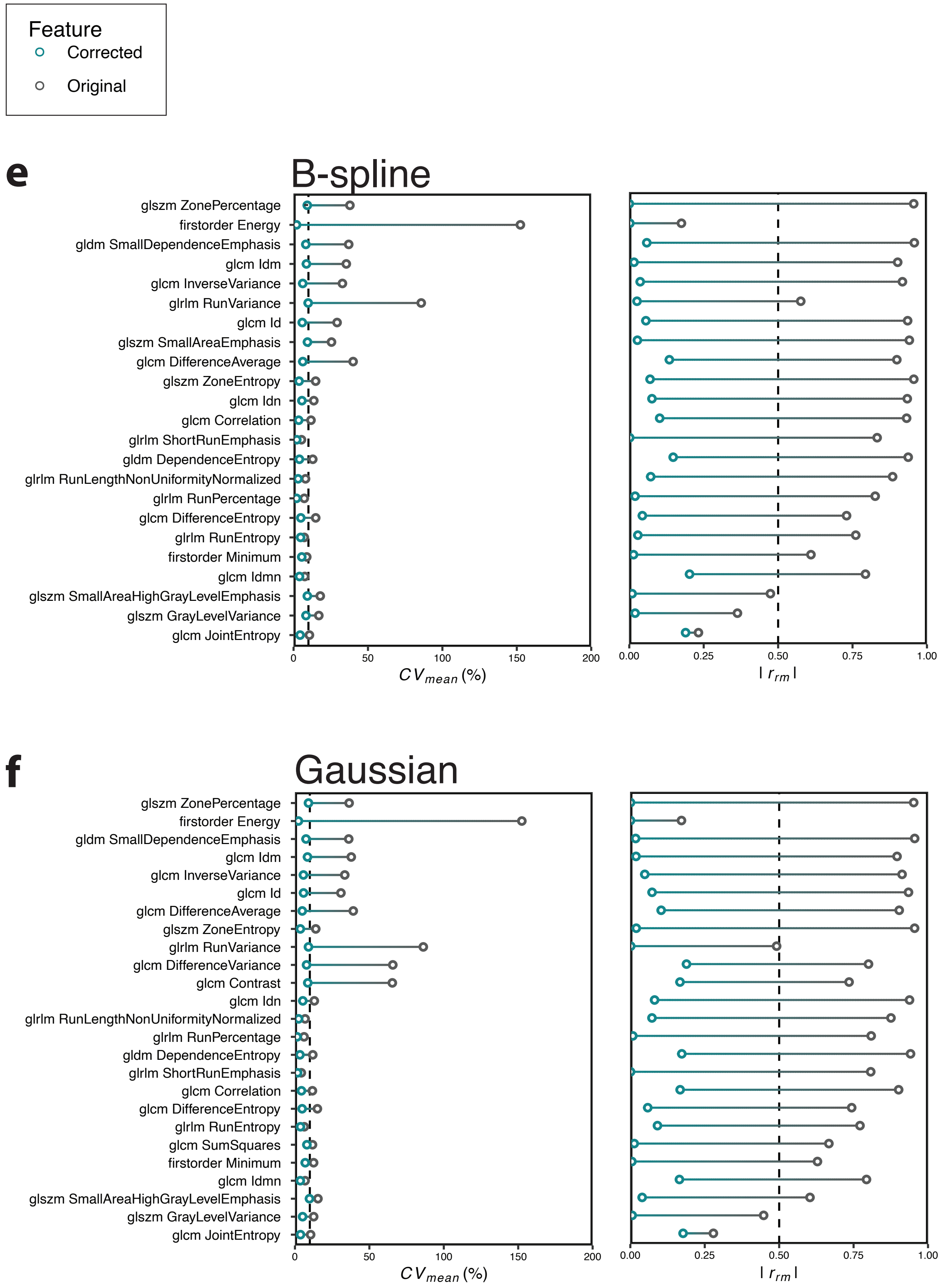
Feature
Corrected
Original
e
B-spline
glszm ZonePercentage
firstorder Energy
gldm SmallDependenceEmphasis
glcm Idm
glcm InverseVariance
glrlm RunVariance
glcm Id
glszm SmallAreaEmphasis
glcm DifferenceAverage
glszm ZoneEntropy
glcm Idn
glcm Correlation
glrlm ShortRunEmphasis
gldm DependenceEntropy
glrlm RunLengthNonUniformityNormalized
glrlm RunPercentage
glcm DifferenceEntropy
glrlm RunEntropy
firstorder Minimum
glcm Idmn
glszm SmallAreaHighGrayLevelEmphasis
glszm GrayLevelVariance
glcm JointEntropy
0 50 100 150 200
CV_{mean} (%)
0.00 0.25 0.50 0.75 1.00
$|r_{rm}|$
f
Gaussian
glszm ZonePercentage
firstorder Energy
gldm SmallDependenceEmphasis
glcm Idm
glcm InverseVariance
glcm Id
glcm DifferenceAverage
glszm ZoneEntropy
glrlm RunVariance
glcm DifferenceVariance
glcm Contrast
glcm Idn
glrlm RunLengthNonUniformityNormalized
glrlm RunPercentage
gldm DependenceEntropy
glrlm ShortRunEmphasis
glcm Correlation
glcm DifferenceEntropy
glrlm RunEntropy
glcm SumSquares
firstorder Minimum
glcm Idmn
glszm SmallAreaHighGrayLevelEmphasis
glszm GrayLevelVariance
glcm JointEntropy
0 50 100 150 200
CV_{mean} (%)
0.00 0.25 0.50 0.75 1.00
$|r_{rm}|$

Supplementary Figure SF9

Dumbbell plots showing the change in CV_{mean} and $|r_{rm}|$ for correctable features in lymphoma, when considering the different discretisation (a-b) and interpolation (c-f) algorithms tested. Features are ranked by the change in feature variability and correlation upon correction.

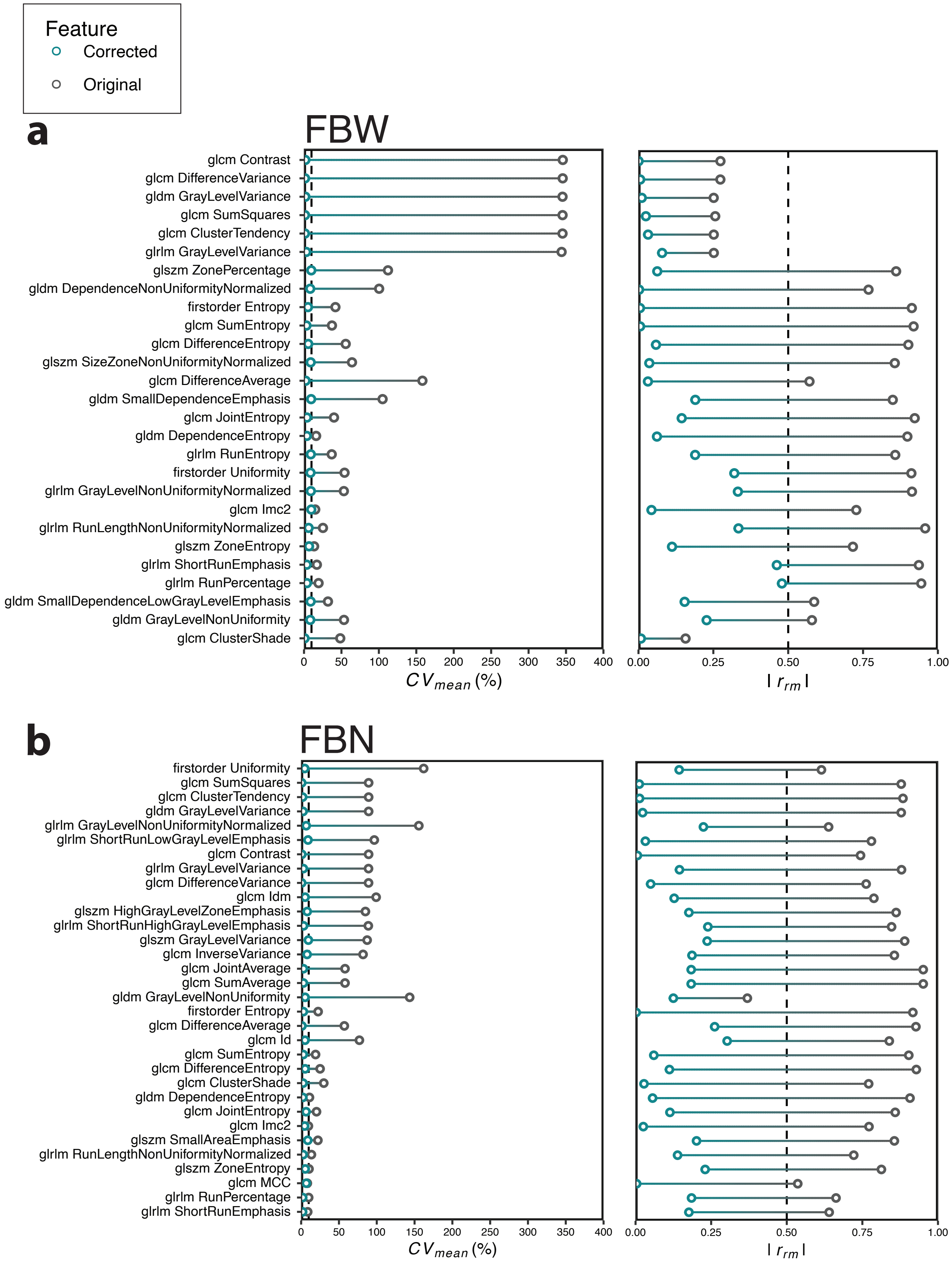

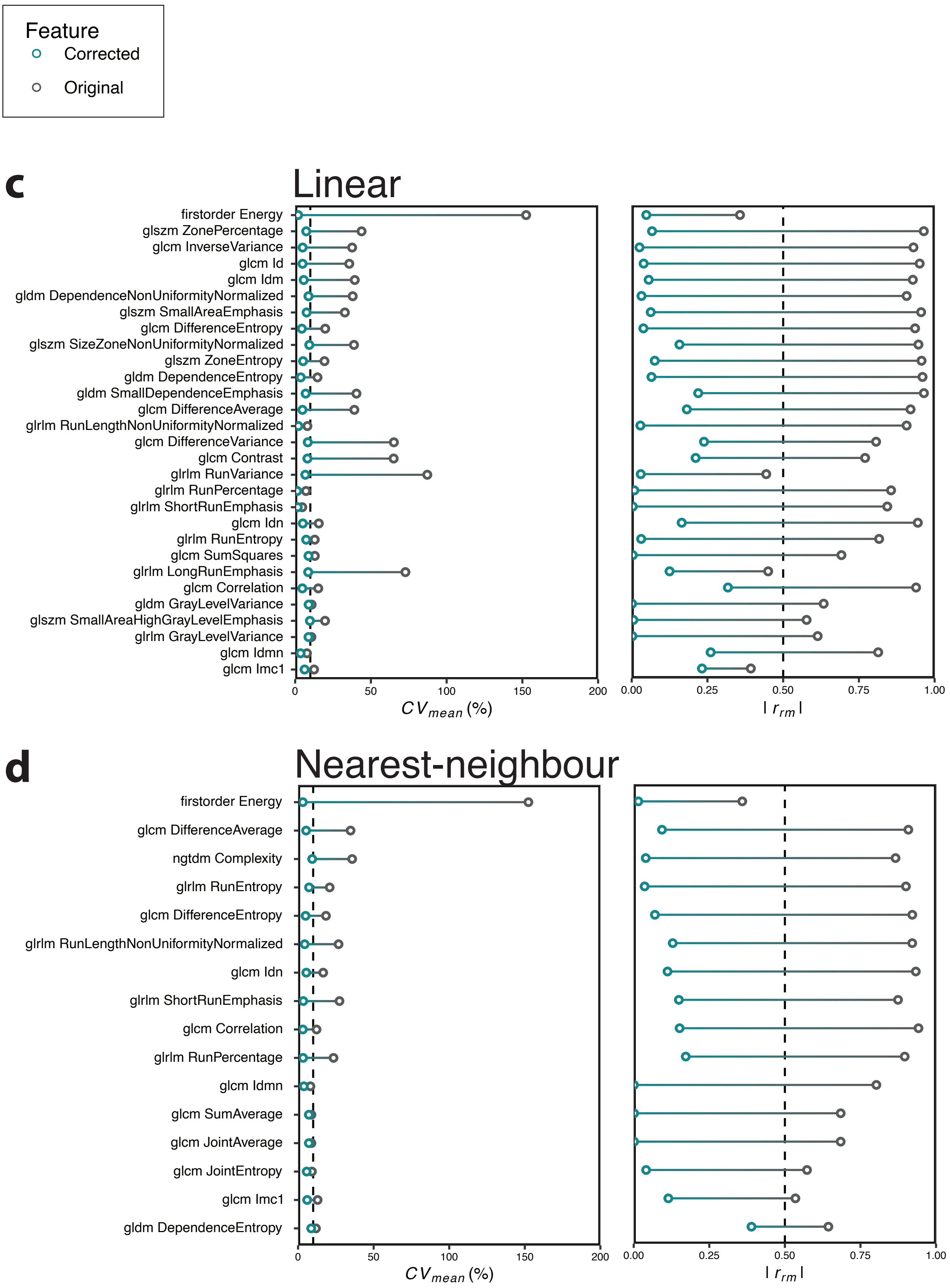

Feature
Corrected
Original
c
Linear
firstorder Energy
glszm ZonePercentage
glcm InverseVariance
glcm Id
glcm Idm
gldm DependenceNonUniformityNormalized
glszm SmallAreaEmphasis
glcm DifferenceEntropy
glszm SizeZoneNonUniformityNormalized
glszm ZoneEntropy
gldm DependenceEntropy
gldm SmallDependenceEmphasis
glcm DifferenceAverage
glrlm RunLengthNonUniformityNormalized
glcm DifferenceVariance
glcm Contrast
glrlm RunVariance
glrlm RunPercentage
glrlm ShortRunEmphasis
glcm Idn
glrlm RunEntropy
glcm SumSquares
glrlm LongRunEmphasis
glcm Correlation
gldm GrayLevelVariance
glszm SmallAreaHighGrayLevelEmphasis
glrlm GrayLevelVariance
glcm Idmn
glcm Imc1
0
50
100
150
200
CV_{mean} (%)
0.00
0.25
0.50
0.75
1.00
$|r_{rm}|$
d
Nearest-neighbour
firstorder Energy
glcm DifferenceAverage
ngtdm Complexity
glrlm RunEntropy
glcm DifferenceEntropy
glrlm RunLengthNonUniformityNormalized
glcm Idn
glrlm ShortRunEmphasis
glcm Correlation
glrlm RunPercentage
glcm Idmn
glcm SumAverage
glcm JointAverage
glcm JointEntropy
glcm Imc1
gldm DependenceEntropy
0
50
100
150
200
CV_{mean} (%)
0.00
0.25
0.50
0.75
1.00
$|r_{rm}|$

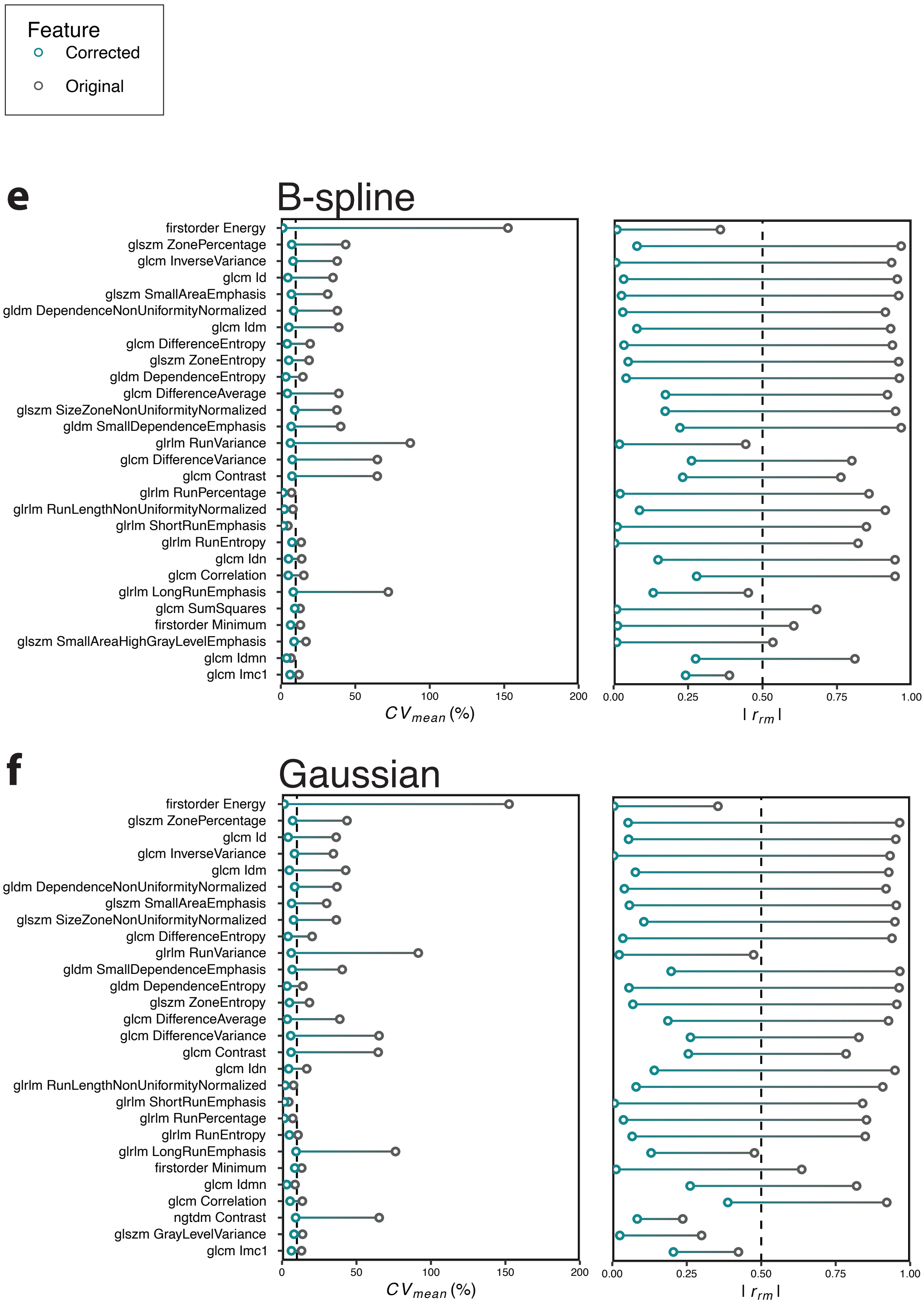
Feature
Corrected
Original
e
B-spline
firstorder Energy
glszm ZonePercentage
glcm InverseVariance
glcm Id
glszm SmallAreaEmphasis
gldm DependenceNonUniformityNormalized
glcm Idm
glcm DifferenceEntropy
glszm ZoneEntropy
gldm DependenceEntropy
glcm DifferenceAverage
glszm SizeZoneNonUniformityNormalized
gldm SmallDependenceEmphasis
glrlm RunVariance
glcm DifferenceVariance
glcm Contrast
glrlm RunPercentage
glrlm RunLengthNonUniformityNormalized
glrlm ShortRunEmphasis
glrlm RunEntropy
glcm Idn
glcm Correlation
glrlm LongRunEmphasis
glcm SumSquares
firstorder Minimum
glszm SmallAreaHighGrayLevelEmphasis
glcm Idmn
glcm Imc1
0
50
100
150
200
CV_{mean} (%)
0.00
0.25
0.50
0.75
1.00
$| r_{rm} |$
f
Gaussian
firstorder Energy
glszm ZonePercentage
glcm Id
glcm InverseVariance
glcm Idm
gldm DependenceNonUniformityNormalized
glszm SmallAreaEmphasis
glszm SizeZoneNonUniformityNormalized
glcm DifferenceEntropy
glrlm RunVariance
gldm SmallDependenceEmphasis
gldm DependenceEntropy
glszm ZoneEntropy
glcm DifferenceAverage
glcm DifferenceVariance
glcm Contrast
glcm Idn
glrlm RunLengthNonUniformityNormalized
glrlm ShortRunEmphasis
glrlm RunPercentage
glrlm RunEntropy
glrlm LongRunEmphasis
firstorder Minimum
glcm Idmn
glcm Correlation
ngtdm Contrast
glszm GrayLevelVariance
glcm Imc1
0
50
100
150
200
CV_{mean} (%)
0.00
0.25
0.50
0.75
1.00
$| r_{rm} |$

Supplementary Figure SF10

Density plots quantifying the overall effect of correction on feature variability and correlation for correctable features, stratified by image processing scheme and cancer type.

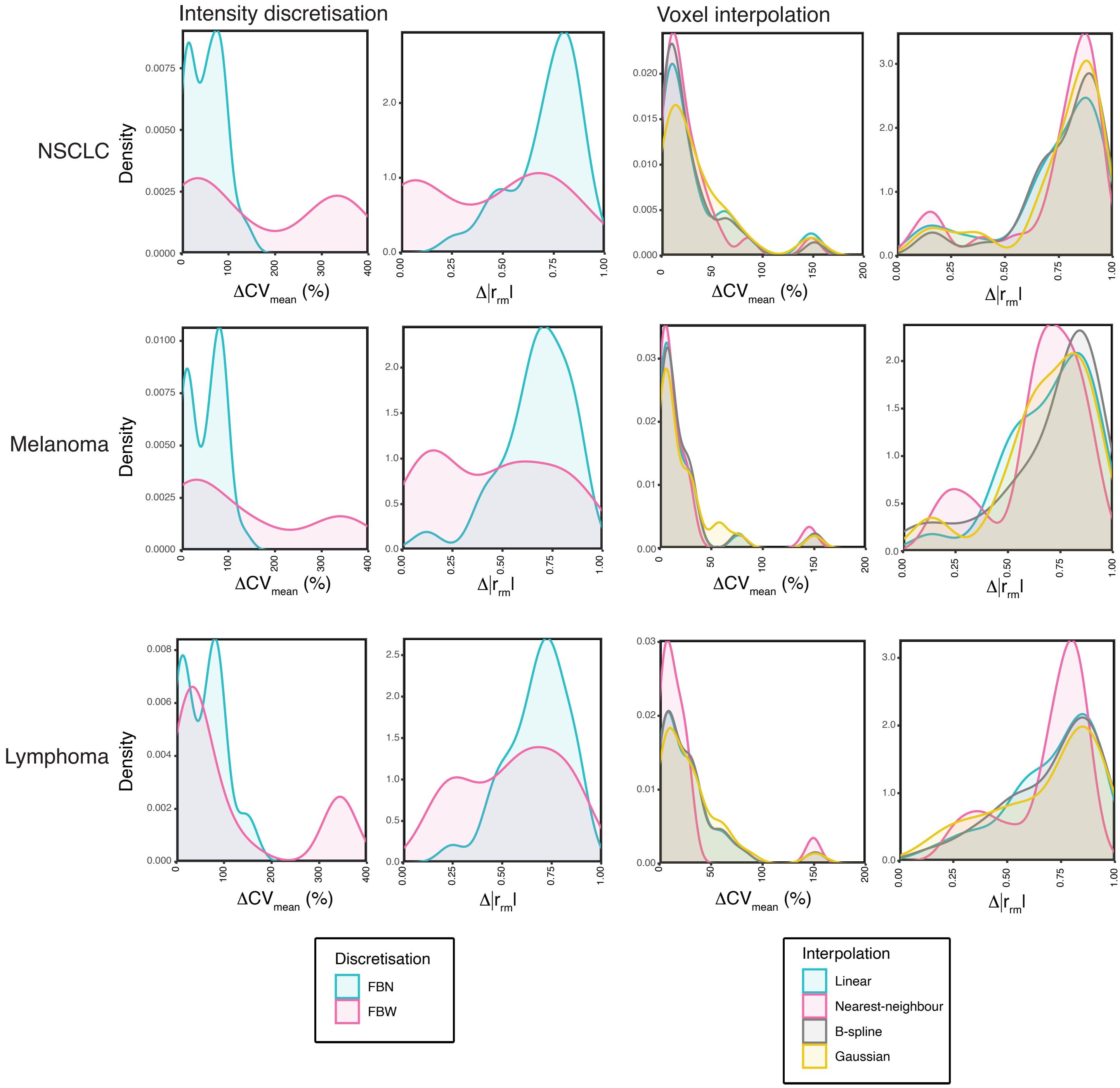

Supplement: Supplementary file 1 — Supplementary Information. [file 41598_2024_67239_MOESM1_ESM.pdf]
